# Comparison of histone-like HU protein DNA-binding properties and HU/IHF protein sequence alignment

**DOI:** 10.1371/journal.pone.0188037

**Published:** 2017-11-13

**Authors:** Dmitri Kamashev, Yulia Agapova, Sergey Rastorguev, Anna A. Talyzina, Konstantin M. Boyko, Dmitry A. Korzhenevskiy, Anna Vlaskina, Raif Vasilov, Vladimir I. Timofeev, Tatiana V. Rakitina

**Affiliations:** 1 Kurchatov Complex of NBICS-Technologies, National Research Center «Kurchatov Institute», Moscow, Russian Federation; 2 Moscow Institute of Physics and Technology, Dolgoprudny, Moscow Region, Russian Federation; 3 Bach Institute of Biochemistry, Research Center of Biotechnology of the Russian Academy of Sciences, Moscow, Russian Federation; 4 Federal Scientific Research Center “Crystallography and Photonics”, RAS, Moscow, Russian Federation; 5 Shemyakin&Ovchinnikov Institute of Bioorganic Chemistry, RAS, Moscow, Russian Federation; Indian Institute of Science, INDIA

## Abstract

**Background:**

The structure and function of bacterial nucleoid are controlled by histone-like proteins of HU/IHF family, omnipresent in bacteria and also founding archaea and some eukaryotes.HU protein binds dsDNA without sequence specificity and avidly binds DNA structures with propensity to be inclined such as forks, three/four-way junctions, nicks, overhangs and DNA bulges. Sequence comparison of thousands of known histone-like proteins from diverse bacteria phyla reveals relation between HU/IHF sequence, DNA–binding properties and other protein features.

**Methodology and principal findings:**

Performed alignment and clusterization of the protein sequences show that HU/IHF family proteins can be unambiguously divided into three groups, HU proteins, IHF_A and IHF_B proteins. HU proteins, IHF_A and IHF_B proteins are further partitioned into several clades for IHF and HU; such a subdivision is in good agreement with bacterial taxonomy. We also analyzed a hundred of 3D fold comparative models built for HU sequences from all revealed HU clades. It appears that HU fold remains similar in spite of the HU sequence variations. We studied DNA–binding properties of HU from *N*. *gonorrhoeae*, which sequence is similar to one of *E*.*coli* HU, and HU from *M*. *gallisepticum* and *S*. *melliferum* which sequences are distant from *E*.*coli* protein. We found that in respect to dsDNA binding, only *S*. *melliferum* HU essentially differs from *E*.*coli* HU. In respect to binding of distorted DNA structures, *S*. *melliferum* HU and *E*.*coli* HU have similar properties but essentially different from *M*. *gallisepticum* HU and *N*. *gonorrhea* HU. We found that in respect to dsDNA binding, only *S*. *melliferum* HU binds DNA in non-cooperative manner and both mycoplasma HU bend dsDNA stronger than *E*.*coli* and *N*. *gonorrhoeae*. In respect to binding to distorted DNA structures, each HU protein has its individual profile of affinities to various DNA-structures with the increased specificity to DNA junction.

**Conclusions and significance:**

HU/IHF family proteins sequence alignment and classification are updated. Comparative modeling demonstrates that HU protein 3D folding’s even more conservative than HU sequence. For the first time, DNA binding characteristics of HU from *N*. *gonorrhoeae*, *M*. *gallisepticum* and *S*. *melliferum* are studied. Here we provide detailed analysis of the similarity and variability of DNA-recognizing and bending of four HU proteins from closely and distantly related HU clades.

## Materials and methods

### Sequence identification and analysis

Members of HU/IHF family proteins were identified by InterPro ID IPR000119, which represents bacterial histone-like proteins. Multiple sequence alignment (MSA) was performed by the rate matrix of residue substitution search using an algorithm described in supporting materials (S1 File) and the Clustal program from UniProt Consortium tools [1]. All HU/IHF sequences were aligned with only exception of Bacteroidetes phylum, where just a half of HU/IHF family proteins sequences gives good MSA [2]. These proteins were further analyzed while phylum Bacteroidetes HU/IHF family proteins which have poor MSA were excluded from further analysis.

### Principal component analysis

Aligned sequences were loaded into seqinr [3] package of the R software environment for statistical computing (https://www.R-project.org). The protein-protein distances were estimated by the dist.alignment function of the seqinr package with Fitch matrix as parameter[4] for similarity estimating. The matrix of pairwise distances was used for principal component analysis (PCA) by built-in R functions. Visualization of the PCA data was done by rgl R package tools[5].

### Purification of recombinant HU proteins

Expression and purification of recombinant HU proteins from S. *melliferum* and *M*. *gallisepticum* have been described in previous works [6,7,8]. Cloning, expression and purification of recombinant HU proteins from N. *gonorrhoeae* and *E*. *coli* were performed similarly. Briefly, the corresponding genes were amplified by a polymerase chain reaction with the oligonucleotides listed in S1 Table using *N*. *gonorrhoeae* genomic DNA and plasmid pBAD-*hupA*, kindly provided by J. Oberto [9], as templates, respectively. PCR products were then digested with restriction endonucleases NdeI and EcoRI and inserted downstream of the T7 polymerase promoter on the His6TEV-HUSpm expressing plasmid [10]. These expressing constructs were transformed into BL21(DE3)RIPL (Stratagene) strain and expression of the target genes fused at the N-termini with 6xHisTEV–tag was induced using 0.4 mM IPTG. After incubation for 18 h at 25°C, *E*. *coli* cultures producing either Spiroplasma, Mycoplasma, Neisseria or Escherichia HU were harvested by centrifugation and the recombinant proteins were purified by Ni–NTA affinity chromatography and digested with TEV-protease. 6xHisTev-tag was removed by the second run of Ni–NTA affinity chromatography and recombinant HU proteins were subjected to final purification and buffer exchange by size-exclusion chromatography.

### Comparative modeling

Modeler software was used for homology modeling using the known structure of HU protein from *Anabaena* sp. (PDB ID 1P71) as a template [11]. Obtained molecular structures were optimized using MolProbity validation tool by adding hydrogen atoms and allowing Asn/Gln/His side chains to flip 180°. The best models were then chosen using MolProbity and ProSa tools. The qualities of homology models have been validated using z-score for overall model quality and analysis of energy distribution along the protein sequence (local model quality). All models and the results of its validation are presented in supporting material (S2–S4 Files).

### DNA sequences

The series of dsDNA fragments with the lengths from 20 to 48 bp were obtained by 3’- truncation of the sequence ‘D-48’: AGTCTAGAGT GCAGTTGAGT CCTTGCTACG ACGGATCCCT TAGGTCAG[12]. 5’-Hex labeled oligonucleotides were annealed with complementary oligonucleotides. Oligonucleotides were synthesized by Evrogen (Moscow, Russia). To obtain DNA junctions, and other distorted DNA structures, Hex-5 labeled oligonucleotide D-48 was annealed with appropriate oligonucleotide/s, which sequences are listed in S1 Table. For example, 3’ DNA overhang was obtained by annealing of D-48 and oligonucleotide ‘J24.rgt’ ACTCAACTGCACTCTAGACT. DNAs were annealed by incubating the oligonucleotides (3−12 μM) for 3 min at 90°C in 20 mM Tris-HCl (pH 8.0) and 200 mM NaCl and then allowing them to cool slowly (∼4 h) to 40°C.

### DNA protein binding

Binding of HU proteins to DNA was tested by electrophoretic mobility shift assays (EMSA) similarly to described earlier[13]. A bound protein retards migration of DNA through the non-denaturing gel, which gives a shifted band of labeled DNA. Fluorescently-labeled oligonucleotide duplexes, from 24 to 48 bp in length (200 nM) or DNA of various structures (10.5 nM) were prepared from non-labeled oligonucleotides and 5’-HEX-labeled oligonucleotides (listed in S1 Table). Varying amounts of HU protein were incubated with DNA for 15 min in 10 ul of the binding buffer, 20mM Tris–HCl (pH 8.0), 7% glycerol, and indicated concentration of NaCl (40 mM or 150 mM). Samples were loaded onto prerun (125 V, 30 min) 8% polyacrylamide gels (29:1) buffered with 50 mM Tris–borate for 40 mM NaCl samples or 100 mM Tris–borate for 150mM NaCl samples and electrophoresed (125 V, 90 min for DNA-binding specificity study or 45 min for binding site size determination). Gels were scanned for visualization using BIO RAD Faros FX Molecular Imager (532 nm EX, 605 nm BP) and quantification was performed using Quantity One software. The binding constants were calculated as described earlier [13,14]. Cooperativity coefficient was estimated as described in [15,16]. The figures show representative results of 3−5 independent assays with each DNA construct.

## Introduction

In bacteria, the proper assembly of higher-order genome structures and DNA topology maintenance require accessory proteins. Nucleoid-associated proteins (NAPs), including LRP, FIS, H-NS, IHF, and HU [17,18,19], are involved in DNA supercoiling and the regulation of gene expression [20,21]. They modulate vital DNA functions such as replication, recombination, repair, and transcription [20,21]. Each species is characterized by a specific set of NAPs, with only HU-like proteins being ubiquitous among bacteria. HU, heat-stable, positively charged protein, is omnipresent in bacteria, and also found in plastid bearing Eukaryota, Euryarchaeota, Thaumarchaeota and some viruses [22]. Majority of bacterial species have homodimeric HU comprised by 10 kDa monomers. In *E*. *coli*, a heterodimeric HU is comprised by the subunits HUα and Huβ [23,24]. Numerous structural studies demonstrated that each HU-dimer has canonical structure comprising alpha-helical compact body and two protruded beta-stranded arms which are required for DNA binding [25,26].

Functions and DNA binding properties of HU-like proteins may vary depending on the specific NAP content and the living conditions of the host[22].Deletion of HU from the *E*. *coli* genome is not lethal unless IHF and H-NS are deleted as well[27]. In contrast, the absence of HU is lethal for organisms in which it is the only NAP available [28,29]. Integration host factor (IHF) is a small heterodimeric protein that binds and bends DNA sequence specifically[30,31,32].HU binds dsDNA in a non-sequence specific manner[33].HU binds DNA in a structure specific manner: it prefers to bind distorted DNA structures such as forks, three- or four-way junctions, nicks, and overhangs[12,16,34,35].The repertoires of preferred substrates vary greatly between the bacterial species [22].

The HU/IHF family of proteins (also referred to Type II DNA-binding proteins) consists of orthologs that share significant sequence homology [31]. Protein sequence alignment and clusterization as well as phylogenetic analysis of thousands HU/IHF proteins, which sequences are available, were performed to identify their taxonomic position, evolutionary connections with other protein families and functionally important structural motives [2]. Search for key amino acids that determine DNA binding properties of HU/IHF proteins is also attractive: HU and IHF sequence comparison suggests key residues for the IHF DNA recognition [2,36]. Small and conservative HU proteins provide useful model for study on the structural basis of thermostability. A number of amino-acid residues were proved to be determinants of thermostability of HUs from *T*. *maritima*, *B*. *stearothermophilus*, *T*. *volcanium* and T. thermophiles [37]; another nature of thermostability was demonstrated for *S*. *melliferum* HU [38].

Here, we perform HU/IHF proteins alignment and clusterization to find HU sequences that represents the most variable HU groups. HU/IHF family is divided into three major groups, IHF_A, IHF_B and HU proteins. These three groups can be further divided into several clades some of which are in good agreement with bacterial taxonomy. Using comparative modeling we built 3D models for a hundred of HU proteins that represent revealed HU clades. Obtained results demonstrated that HU fold remains conservative for HU from both closely and distantly related clades and taxonomic groups.

Finally, we compared DNA–binding properties of either closely or distantly related HU proteins. For this study we choose well characterized *E*. *coli* HUα [24] and non-characterized HU representatives from *N*. *gonorrhoeae* which sequence is similar to *E*. *coli* HU as well as HU proteins from two Mollicutes species: *M*. *gallisepticum* and *S*. *melliferum* that belong to remote branch of HUs. Mollicutes are the smallest known microorganisms which characterized by the absence of the cell wall and very small genome sizes (from 0.58 to 1.4 Mb) [39]. High genome plasticity of Mollicutes [40] results in high diversity of their protein sequences, including those of HU proteins. *M*. *gallisepticum* HU has multiple amino acid substitutions in the most conservative regions [8,41], while *S*. *melliferum* HU has increased number of phenylalanine residues and enhanced hydrophobic interactions in its dimeric interface [38]. We found that in respect to dsDNA binding, only *S*. *melliferum* HU binds DNA in non-cooperative manner and both mycoplasma HU bend dsDNA stronger than *E*.*coli* and *N*. *gonorrhoeae*. In respect to binding of distorted DNA structures, each HU protein has its individual profile of affinities to various DNA-structures with the increased specificity to DNA junction.

## Results and discussion

### Alignment of HU/IHF family protein sequences

HU/IHF family protein sequences and annotation were acquired from InterPro ID IPR000119, which represents bacterial histone-like proteins. Results of multiple sequence alignment (MSA) of HU/IHF family proteins sequences are presented in (S3 Table). This table includes all HU/IHF family protein sequences from InterPro ID IPR000119 with indicated position of consensus sequence and position of possible insertion/deletion. We also attributed a taxonomy classification of the species [NCBI Taxonomy. https://www.ncbi.nlm.nih.gov/taxonomy]; totally 2000 species (25 phyla) with annotated HU/IHF family protein are listed. We hope that this easy-to-use table will help researchers to itemize HU/IHF of interest.

Each aligned protein sequence belongs to one of three major groups: HU proteins, IHF_A or IHF_B proteins. Supporting S3 Table indicates for each aligned sequence its attribution to these major groups as well as InterPro annotation of the protein [EMBL-EBI https://www.ebi.ac.uk/interpro/]. Comparison of our alignment and clustering results with InterPro annotation shows that actual annotation of HU/IHF proteins is rarely inaccurate:2% of HU sequences are erroneously annotated as IHF, and 1% of IHF_A or HF_Bare annotated as HU. Although, one third of HU/IHF proteins are annotated as “DNA-binding protein”; without further attributing the proteins to HU, IHF_A or IHF_B. We show here that any HU/IHF protein can be unambiguously attributed to HU, IHF_A or IHF_B: for each sequence its scores are significantly different for these three groups. This result is well correlated with phylogenetic analysis performed previously [2]. In this study comprehensive evolutionary and structural analysis was applied to understand the differences between DNA-binding mode of HU and IHF proteins. Here, we perform further subdivisions of the three major clades and detailed analysis of high variability of HU proteins sequences and DNA-binding features.

**Fig 1:**
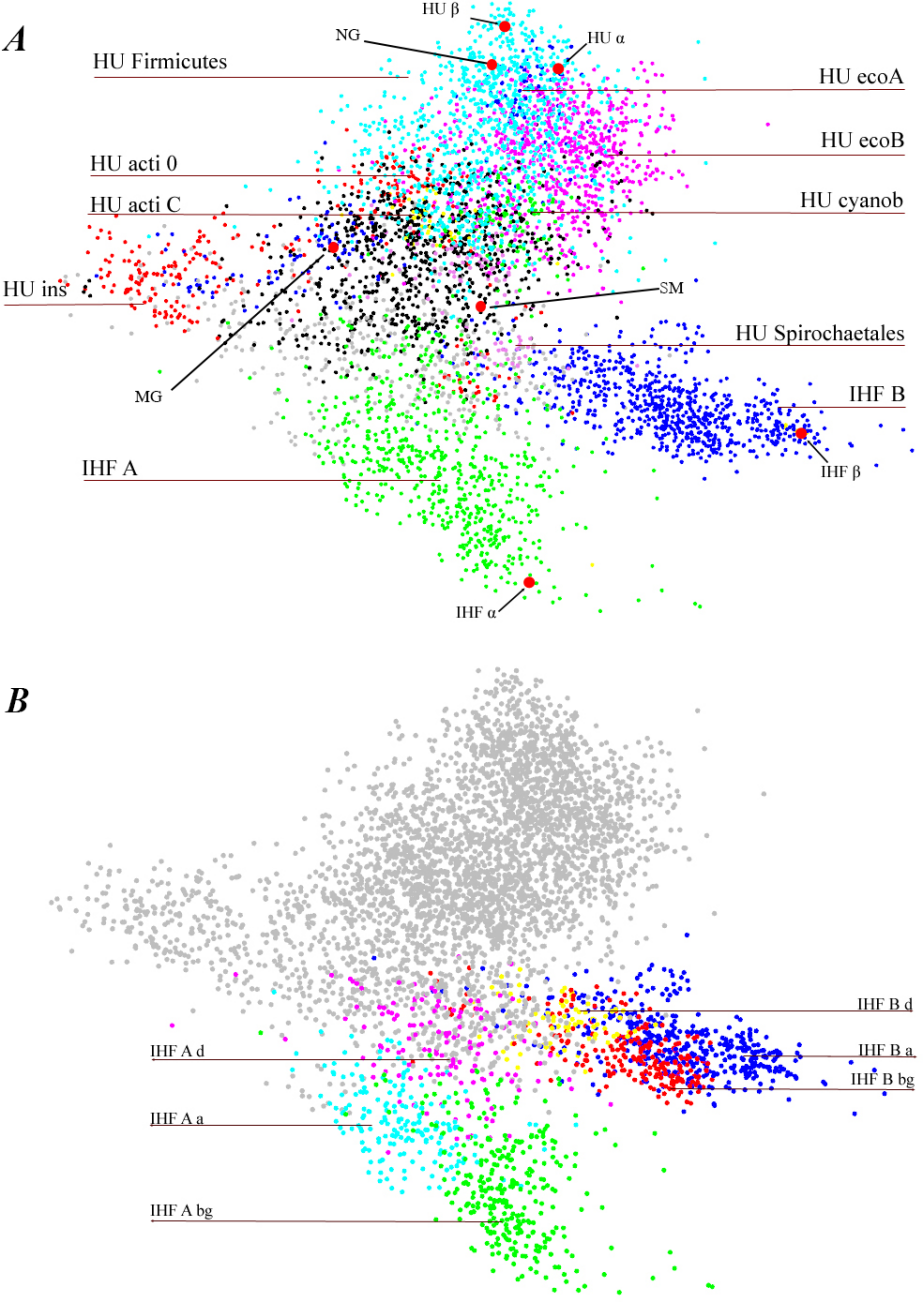
PCA plot of the location of aligned sequences of HU/IHF proteins on three axes. Three main groups of proteins, HU, IHF_A and IHF_B, are indicated as well as results of further subdivision of the protein sequences (HU and IHF clades).**A.** Most populated HU clades: clade HU_Firmicutes (mainly originated from HU of Firmicutes species) and clades HU_ecoA and HU_ecoB (mainly from proteobacteria) are shown in magenta, bleu and cyan, respectively. Other apparent HU/IHF clades are indicated. Position HUs and IHFs proteins of *E*. *coli* are shown in red, as well as positions of HU of *N*. *gonorrhoeae* (NG), *M*. *gallisepticum* (MG), and *S*. *melliferum* (SM). *E*. *coli* HUα and Huβ are very close to each other (62 identities in 90 amino acid core sequence), and IHFα and IHFβ are far from each other (24 identities of 90). We believe that it is the reason why HU separation onto two groups, one close to HUα, and another close to HUβ, is ambiguous. **B.** IHF clades. Result of two major IHF group subdivisions shown in color: IHF_A magenta, cyan and green, IHF_B red, yellow, and blue. HU sequences are shown in grey.

For visualization of the results of HU/IHF family MSA and clusterization we employed principal component analysis (PCA), a powerful method for the dimensional reduction and analysis of large data sets (see [42] and references within). PCA uses a vectorial representation of each protein sequence as a point in a multidimensional space and allows reducing of the number of dimension. Distances between aligned sequences were calculated according to amino acid identities at all 90 positions of the protein sequence and PCA was performed (Fig 1). Sequences of IHF_A and IHF_B are well separated and essentially differ from HU proteins.

At present, HU/IHF family sequences are classified as HU_A, HU_B, IHF_A and IHF_B or as DNA–binding proteins when sequence was not attributed to one of the four groups. Alternatively, a similar classification system is used—hup1, hup2, IHF_A, IHF_B. HU protein subdivision onto hup1 and hup2 (or HU_A and HU_B) is ambiguous as reference sequences, which historically were *E*. *coli* HUα and Huβ proteins, are too close to each other (62 identities of 90 HU positions) to serve as a basis for the whole family HU/IHF sequences cauterization (Fig 1A). We believe that in actual InterProt HU annotation about a half of HU sequence annotations must be changed from HU_A/HU_B or hup1/hup2 to HU.

### IHF proteins

We found that IHF proteins are specific exclusively for proteobacteria; see also [2,32]. Out of proteobacteria phylum, we detected only a few bacterial species that contain IHF sequences as well as few Eukaryota species (see S3 Table). IHF proteins are well–separated into two groups, IHF_A and IHF_B (Fig 1A). *E*. *coli* IHF sequences are, by chance, well placed to entitle IHF proteins: *E*. *coli* IHFα and IHFβ have only 24 identities of 90 amino acid residues within the core sequence in contrast to *E*. *coli* HUα and HUβ.IHF_A sequences are further subdivided to three clades (Fig 1B), closely related to proteobacteria taxonomy. IHF_A_bg clade is populated at 99% by IHF_A sequences from beta-proteobacteria and gamma-proteobacteria classes (beta/gamma proteobacteria, Gram-negative). IHF_A_aclade coincides with IHF_A of alpha-proteobacteria. IHF_A_d is mainly populated by IHF_A of delta-proteobacteria and acidithiobacillia (see also[32]).

Similarly, IHF_B sequences can be subdivided to three clades that correspond to proteobacteria classes. IHF_B_bg clade coincides with IHF_B sequences from beta-proteobacteria and gamma-proteobacteria classes as well as acidithiobacillia class. IHF_B_a coincides with IHF_B sequences from alpha-proteobacteria. IHF_B_d coincides with IHF_B sequences from delta-proteobacteria. IHF_A and IHF_B classifications are similar, only acidithiobacillia IHF_A is close to IHF_A of delta-proteobacteria while acidithiobacillia IHF_B is closer to IHF_B of beta-proteobacteria and gamma-proteobacteria.

Note some interesting exceptions when IHF can be found outside of proteobacteria. *Nitrospinagracilis*is belongs to *Nitrospinae* phylum. *Nitrospinagracilis* contains five HU/IHF family proteins: HUs and both IHFs, IHF_A_d and IHF_B_a. Other exeption is *Thermodesulfovibrioyellowstonii* (Nitrospirae phylum), it contains three HU/IHF family proteins, one HU and two IHFs, IHF_A_dand IHF_A_d,both characteristic for delta-proteobacteria. Among Eukaryotes containing IHF proteins we note *Capitellateleta* and *Castor bean*; see S3 Table for few other examples of IHF out of proteobacteria.

### HU proteins

**Fig 2:**
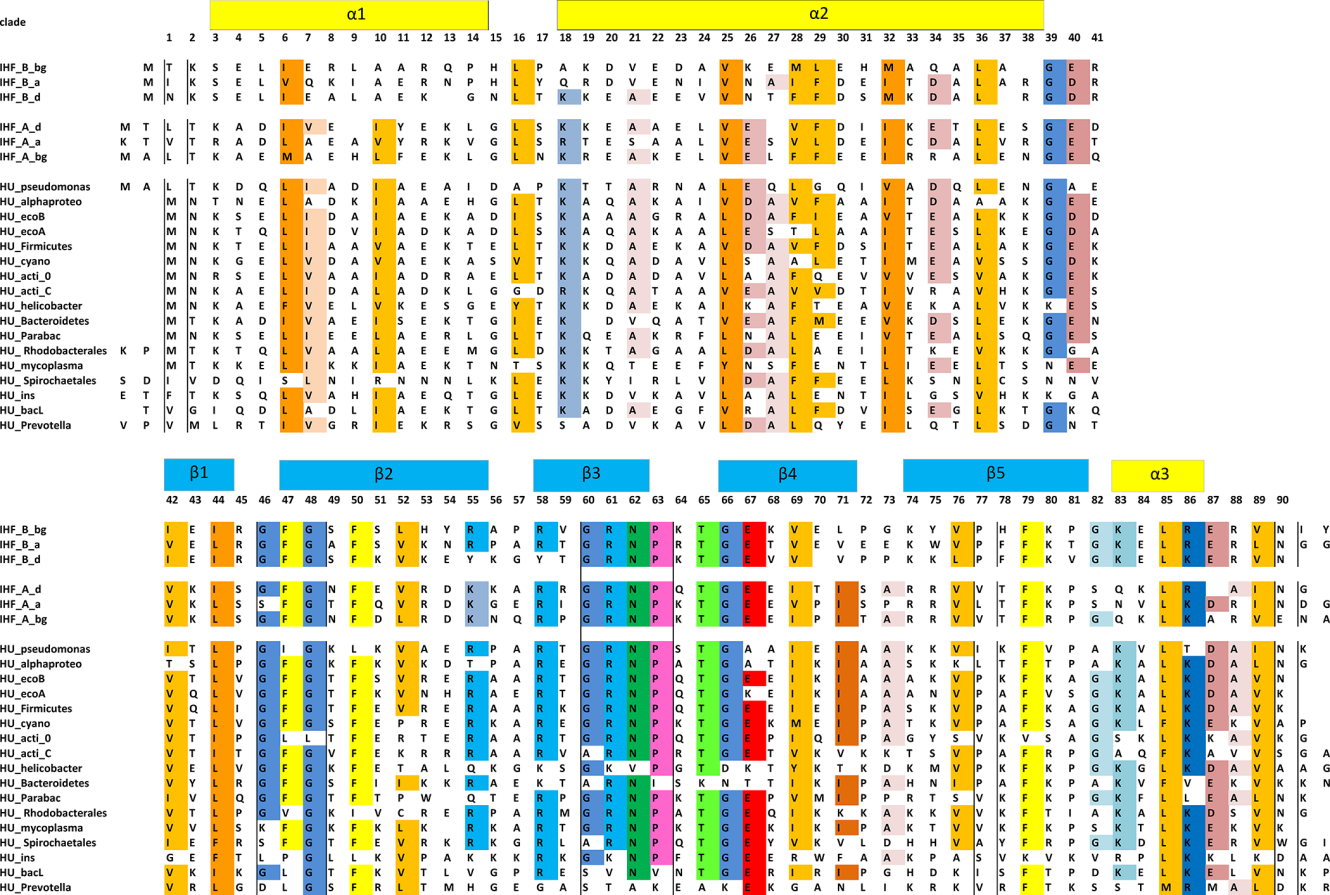
Consensus sequences of three IHF_A, three IHF_B, and several HU clades revealed in this study. Conservative residues are highlighted. Alpha helixes and beta sheets are indicated with yellow and blue blocks, respectively.

Subdivision of HU sequences is not as unambiguous as it is for IHF where clusterization is in very good agreement with taxonomy data. Nevertheless, we present results of clusterization 1) to demonstrate HU variability and 2) to describe the most obvious HU clades which identity is apparent. HU clade represents a totality of HU sequences which are similar to each other and, hence, to the core consensus motif. Consensus sequences that represent revealed HU and IHF clades are shown in Fig 2. Attribution of all HU/IHF sequences to these clades is indicated in S3 Table.

Actinobacteria phylum is a perfect example of HU clusterization. All HU proteins consist of HU 90 amino acid core, some HU have N- and C- terminal extensions out of core sequence. Mycobacterium genera HU proteins consist of HU core with characteristic consensus motif “HU_acti_C” (Fig 2) and a long C-terminal extension. Among HU sequences of Corynebacterialesorder (that includes *Mycobacterium*, *Gordonia* and other genera) we could not observe any HU homologues which do not belong to HU_acti_C clade.All of them contain also C-terminal extension of 36–158 amino acids. Obviously, the only form of HU protein of Corynebacterialesorderis HU that belongs to the HU_acti_C clade. Properties of Mycobacterium HU C-termini are described in details [2,22]. In *Streptomyces* genus this HU from HU_acti_C clade, also with C-terminal extension (88–150 long), is expressed during spore maturation [21,24]. These bacteria possess another HU paralog that belongs to another HU clade “HU_acti_0” (Fig 2); it has no C-terminal extension. This HU paralog is expressed during the growth phase [43]. HU_acti_0 sequence is specific exclusively for Actinobacteria phylum (few exceptions include *Bacillus phage SP01* and *Cellvibriogilvus)*. Similarly, HU proteins of HU_acti_C clade were found exclusively in Actinobacteria phylum, even if only core sequence (without C-terminal extension) is taken into account. Thus, Actinobacteria HU/IHF sequences classification on two HU clades is exemplary classification of the proteins: each Actinobacteria HU/IHF is unambiguously attributed to HU_acti_C or to HU_acti_0clade and all HUs from these clades belong to Actinobacteria.

We analyzed how Actinobacteria HU variants are distributed among Actinobacteria species. Three situations were observed:

1. Bacteria of orders: Bifidobacteriales, Actinomycetales, Geodermatophilales, Acidimicrobiia, Coriobacteriia, contain only HU proteins of clade HU_acti_0.
2. Bacteria of orders: Acidothermales, Catenulisporales, Corynebacteriales (including Mycobacteria), Glycomycetales, Micromonosporales, Nakamurella, and Pseudonocardiales contain only HU proteins of clade of HU_acti_C with long C-terminal extension. Interesting that HU_acti_C HU protein can be functional without C-terminal extension: Frankie’s contain only one HU/IHF polypeptide, HU_acti_C, while their HU C-terminal extension has just 3–4 residues. Similarly, the only one HU/IHF polypeptide observed in Streptosporangiales is HU_acti_C with a short C-terminal extension.
3. In orders: Kineosporiales, Micrococcales, Propionibacteriales, and Streptomycetales we found HU polypeptides of both clades, HU_acti_0 and HU_acti_C.

### HU classification

We entitle HU clades according to the taxonomy group which is the most present in this clade. Two large HU clades are HU_Firmicutesand HU_ecoB (see Fig 1A). Clade HU_Firmicutesis populated at 95% by Firmicutes, clade HU_ecoB is populated at 94% by Proteobacteria. These clades are not matchexactly with taxonomic groups: Proteobacteria as well as Firmicute species have HU sequences of other clades (see S3 Table).

Bacteria of Pseudomonas genera (class gamma-proteobacteria)carry four HU/IHF proteins, IHF-A, IHF-B and two HU proteins, one HU belongs to HU_ecoB clade and is similar to HU from many other classes, another belongs to HU_pseudomonas clade and is specific for Pseudomonas. Only one such protein was found elsewhere (in Fungi). Consensus sequence of HU_pseudomonas clade is presented in Fig 2.

**Fig 3:**
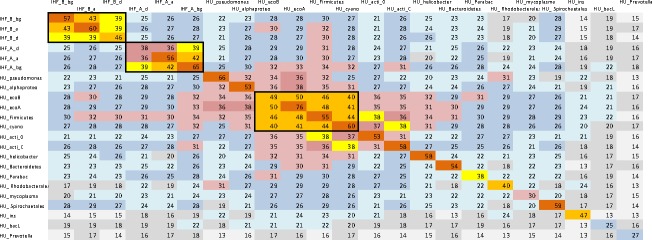
Average number of identic amino acid residues in protein sequences of HU/IHF clades. Number of identic amino acid residues in protein sequences was calculated for each pair of HU/IHF proteins. Each cell represents an average number of identic amino acids for sequences from two clades. Only residues within 90 amino acid long core of HU/IHF were taken into account; non-equivalent insertion or deletion in protein sequence was calculated as one non-identic residue.

Spirochaetales order (mainly Borrelia) carries HU of HU_Spirochaetales clade as a single HU/IHF protein specific exclusively for this order. HU_Spirochaetales clade members distances to other HU are similar to their distances to IHFs. See Fig 3, which shows average number of identic amino acid per 90 residues long core of the protein.

Species from Mollicutes class have only one HU polypeptide that belongs to HU_mycoplasma clade; HU of this clade are observed exclusively in Mollicutes. Of note, one order among Mollicutes, Entomoplasmatales, which includes Spiroplasmas, possess more common HU from HU_ecoB clade. The comparative analysis of DNA—binding properties of *Spiroplasma melliferum* HU protein (HU_ecoB clade) and *Mycoplasma gallisepticum* HU protein (HU_mycoplasma clade) is presented below.

Species of class Cyanobacteria have only one HU/IHF protein, HU_cyano that essentially differs from other HU and IHF. Vice versa, HU_cyano motif is found exclusively in Cyanobacteria. Two interesting exceptions are eukaryotes *Paulinellachromatophora* (Cercozoa) and *Rhodomonassalina* (Cryptomonads).

*Helicobacter* and all Helicobacteraceae family species possess only one HU/IHF polypeptide. All these proteins belong to HU_helicobacter clade. This clade is specific for Helicobacteraceae family. Other Campylobacterales bacteria possess HUs that does not belong to HU_helicobacter clade.

Rhodobacterales (class alpha-proteobacteria) possess both IHF subunits, IHF_A_a and IHF_B_a, and only one HU sequence, mainly it has HU_ Rhodobacterales motif that essentially differs from other HU and IHF (Fig 3). Interesting that some Rhodobacter phages have the same HU motif, while usually phages encode for HU sequence that are essentially different from HU of the host bacterium. HU_ Rhodobacterales clade is specific for Rhodobacterales and Spirochaetes (mainly, Leptospirales) as well as for eukaryotes of class Dinoflagellate (Alveolata). Unlike to Rhodobacterales, Dinoflagellate contains only one HU/IHF polypeptide, HU_ Rhodobacterales, and no other HU/IHF members, including IHF. It is surprising that the same HU_ Rhodobacterales HU motif is able to play a role of HU and IHF in Dinoflagellate, while it must collaborate with IHF in Rhodobacterales.

HU_insclade is populated by alpha-, beta-, and gamma-proteobacteria, mainly Rhizobiales, Burkholderiales, and Xanthomonadales. Its core sequence is the most remote from all other HU (Figs 1 and 3).

HU/IHF family proteins from phylum Bacteroidetes gives good MSA only in 60% cases. Further analysis of these sequences, reveals some clades, HU_Parabac, HU_Prevotella, and HU_bacL, specific only for Bacteroidetes (S3 Table and Fig 2).

### N- and C- terminal extensions of HU

The most described HU/IHF terminal extension is С-termini of Actinobacteria, clade HU_acti_C. In average, it contains 111 residues, including 29 lysines and 5 arginines, and only 0.4 and 0.7 aspartic and glutamic acids, respectively. HU_acti_CС-termini are associated with a lysine rich ‘‘PAKKA” repeat, this repeat is implicated in protection of DNA from adverse conditions [2,44]. This PAKKA repeat is also present in two genera from another phylum, Variovorax and Thiovulum (S3 Table).We found PAKKA sequence at the N-termini of Burkholderiales (clade HU_ins). This repeat is presented also in Deinococci [21]. PAKKA motif (not repetitive) exists within the core HU sequence of clade HU_insand in Fusobacteria, where the intercalating proline’s at the HU arms tips are included in PAKKA.

Besides Actinobacteria, only Bacteroideteshave C-termini longer than 48 residues (with rare exceptions including Anabaena, Deinococcus and IHF-beta of Ralstonia). Bacteroidetes HU core sequence is essentially different from HU_acti_C. Most of Bacteroidetes HUs with long C-terminal extension constitute a clade HU_bacL characterized by long, 238 residues in average, C-termini with very high content of charged residues: 19 lysine sand 9 arginines, as well as 13 aspartic and 31 glutamic acids. Often it contains proline’s and two consecutive lysine’s that make them similar to PAKKA motif.

HU_bacL HU proteins have extended N-termini, which are 1 to 4 residues or around 30 residues long. Most of HU_insclade proteins possess long N-terminal extension, 40 residues in average; with high lysine content (in average, 10 lysine’s per terminus). Other HU proteins with long N-termini are found among Bacteroidetes, Mollicutes and Deinococci. IHF_A of Burkholderiales and Rhizobiales also possess long N-termini. Usually long IHF/HU N-termini have several negatively and positively charged residues.

Charged residues, especially positively charged, at C- and N- terminal extensions of the HU/IHF proteins are able to modulate protein-DNA interactions [13].

### Insertions and deletions

**Fig 4:**
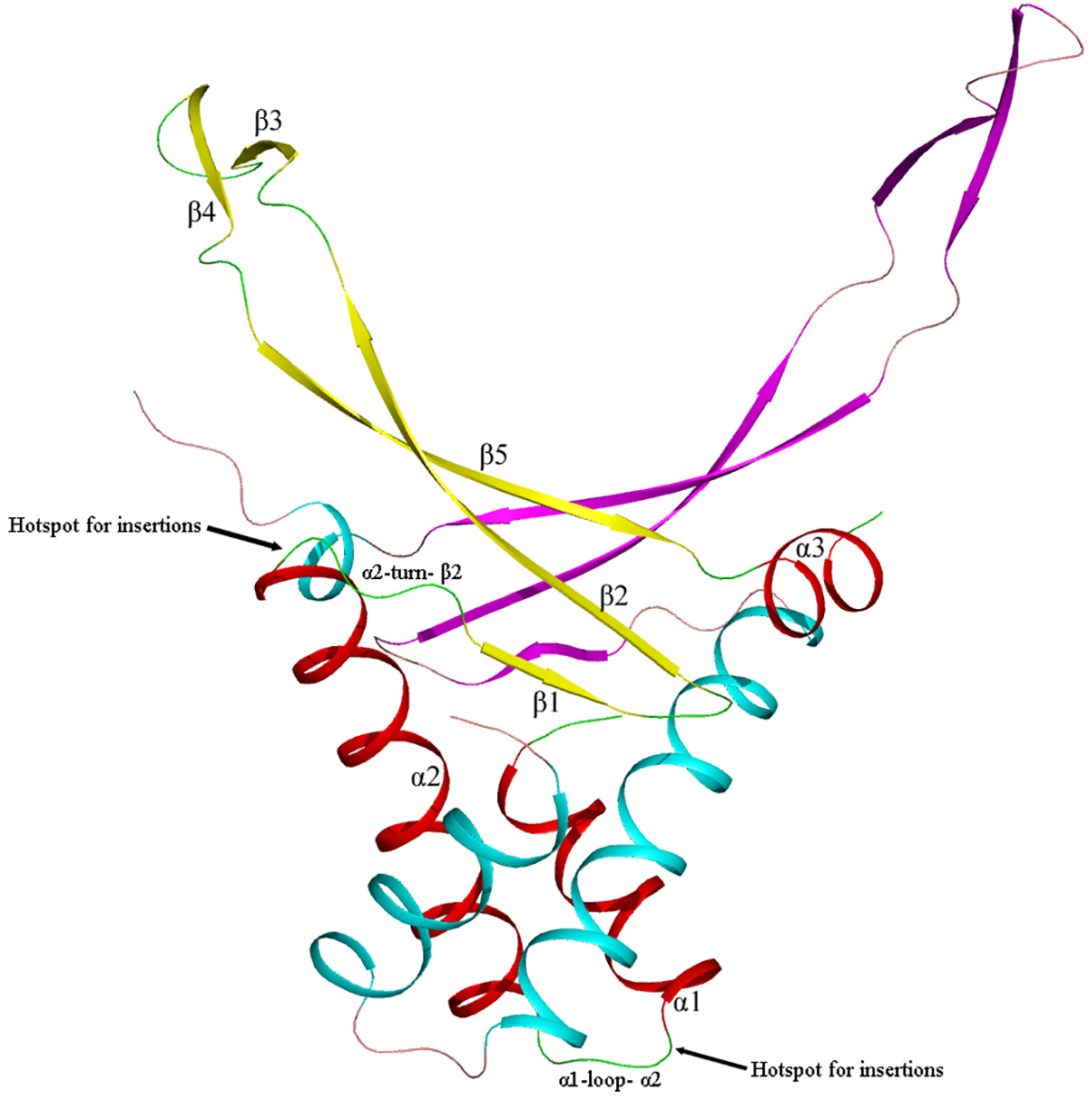
Model of *E*.*coli* HUα dimer with hotspots for amino acid insertions and deletions. Each HU monomer contains three alpha helixes and five beta strands. HU body (helixes 1 and 2) is responsible for dimer stabilization. HU arms are responsible for DNA binding. Hotspots for amino acid insertions and deletions in HU are shown.

HU/IHF sequences can contain amino acid inserts and deletions compared to consensus sequences. About 10% of HU proteins have an insert, usually of one amino acid (80% of inserts). Insert position distribution along the HU sequence is far not uniform. The most frequent position for insertion is a loop between alpha helixes 1 and 2 (34%). Turn between helix 2 and beta strand 1 (18%) as well as DNA–interacting tip (15%) of HU are also hotspots for amino acid insertions (Fig 4).

Most frequent position for insertion is a loop between alpha helixes 1 and 2 (α1-loop-α2 in the bottom of figure). Turn between helix 2 and beta strand 1 is the second hotspot for insertions. We believe that indels at these positions does not change essentially HU fold.

Though average insertion rate is low, all HU sequences of several clades have amino acid insertions: in majority of phytoplasma HU one amino acid residue is inserted between beta strands 1 and 2. All sequences of HU_Spirochaetales clade contain one amino acid insertion in beta-strand 3. Most Dinoflagellata (Eukaryota) HU sequences (clade HU_Rhodobacterales) also contain one amino acid insert in beta-strand 3. All sequences of HU_insclade HU contain 5 or 3 amino acid insert in beta strand 2.

About 3.4% of HU proteins have a deletion of one amino acid; longer deletions are rare. Majority of deletions (70%) are localized within the loop between alpha helixes 1 and 2. Turn between helixes 2 and beta strand 1 also can contain deletions (7%).About a half of HU sequences of clade HU_acti_Chave an amino acid deletion within the loop between helixes 1 and 2, they are responsible for 67% of deletions observed in HU proteins.

IHF_A and IHF_B also contain insertions, insertion rates are 3,8% and 2.4%, respectively. Again, most insertions are localized in the loop between helixes 1 and 2. Deletions in IHF_A are rare (0.5%), all within the loop between helix 1 and 2; among IHF_B proteins only IHFs of the clade IHF_B_d contain deletions (6% sequences), all are within the loop between helix 1 and 2.

### HU models

To estimate how the sequence differences between HU proteins influence their 3D folding we performed comparative modeling (CM) using the known structure of HU protein from Anabaena sp. (PDB ID 1P71) as a template. Because of broad spectrum of HU sequences we built and analyzed 103 models in overall (at least four models for each of the 14 HU clades). All models and their validations are available from supporting materials (S3 and S4 Files). To estimate accuracy of our CM-evaluation, some models were compared with the experimentally solved X-ray structures. For example, for *S*. *melliferum* HU protein, RMSD between model and crystal structure was 0.1348 nm.

**Fig 5:**
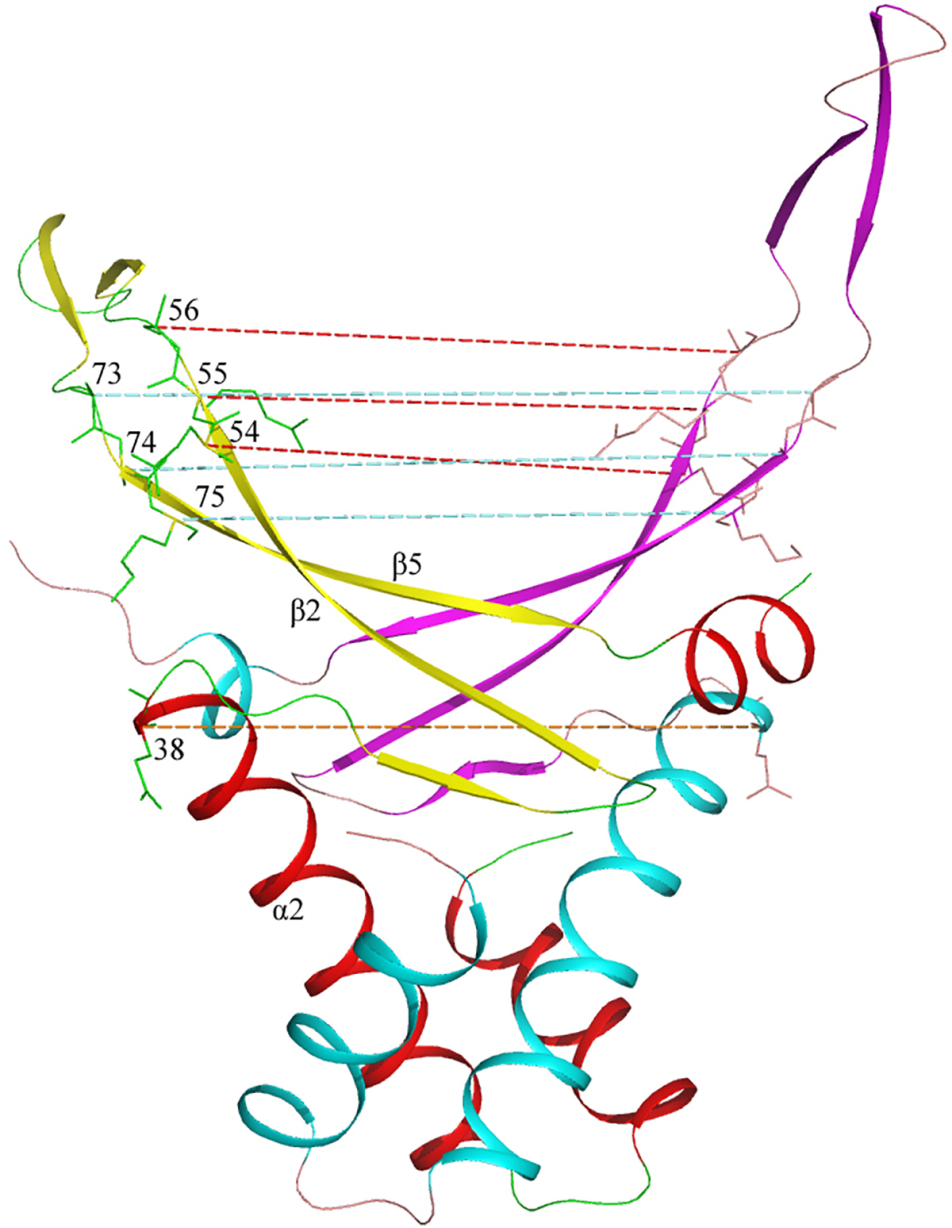
Model of *E*.*coli* HUα dimer with distances between C-alpha atoms that were measured. Positions of amino acid residues corresponding to the C-termini of alpha helixes 2 are indicated and distance between corresponding C-alpha atoms of both monomers is shown with khaki dotted line. Positions of the amino acid residues at the C-termini of beta strands 2 and N-termini of beta strands 5 as well as their neighboring residues are indicated and the corresponding distances between C-alpha atoms of both monomers are shown with rose and blue dotted line, respectively.

Comparative analysis of the models was aimed to determine several parameters of both alpha-helical body and beta-stranded DNA-binding arms of HU dimer. We measured angles between either alpha helixes 1 and 2 or 2 and 3 of the same monomer as well as an angle between alpha helixes 2 of opposite monomers. We also measured distances between C-terminal ends of alpha helixes 2, C-terminal ends of beta strands 2 and N-terminal ends of beta strands 5 of opposite monomers. See Fig 5 for illustration.

Alpha helixes 1 and 2 (12 and 21 amino acid residues long, respectively) include more than two thirds of HU monomer residues; angle between alpha helix 1 and 2 determine the architecture of the HU protein body. The angle between the long alpha helixes 2 of two HU monomers that form a dimer determines reciprocal orientation of HU subunits.

**Fig 6:**
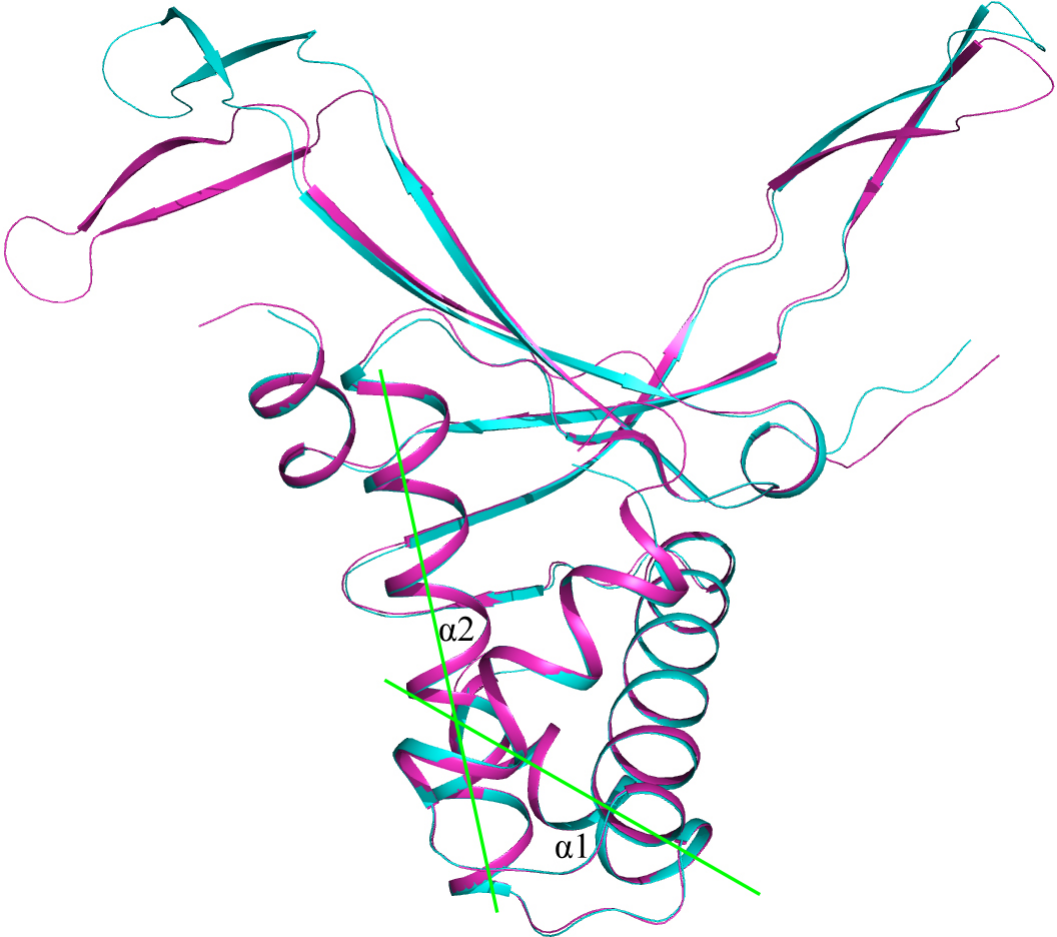
Model of *Pseudomonas syringae* HU (magenta) superimposed with *E*. *coli* HUα model (cyan). Angle between alpha helix 1 and alpha helix 2 is shown in green.

Angle between alpha helix 1 and alpha helix 2 constitutes 61.5 to 63.6 degrees (62.7 degrees in average) and is equal for all the HU models analyzed with few exceptions (Fig 6). In HU proteins of majority of Pseudomonas (clade HU_Pseudomonas) this angle is significantly less than in other HU proteins and constitutes from 55.8 to 56.7 degrees (56,1 degrees in average). Similarly, in HU of majority of Rhodobacterales (clade HU_Rhodobacterales) this angle constitutes from 59.0 to 61.1 degrees (59.5 degrees in average).

**Fig 7:**
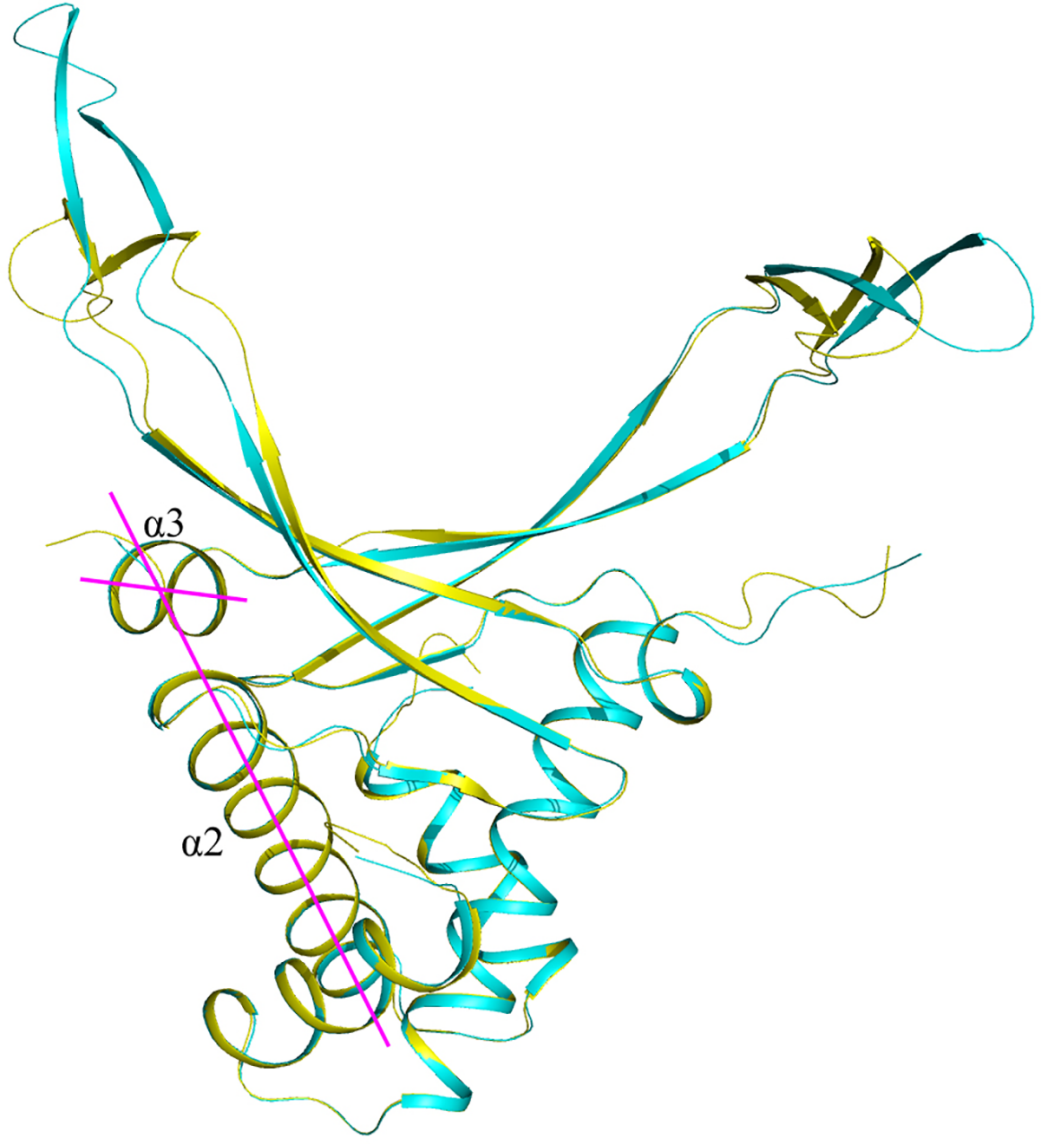
Model of HU *Bifidobacterium longum* from Actinobacteria (clade HU_acti_0), superimposed with *E*. *coli* HUα model. *B*. *longum* HU is shown in yellow, *E*. *coli* HUα—in cyan. Angle between alpha helix 2 and alpha helix 3 is shown in magenta.

Angle between alpha helix 2 and alpha helix 3 of the same monomer constitutes from 61.8 to 64.4degrees (63.7 degrees in average) and is equal for all the HU clades analyzed with one exception (Fig 7). An important exception is HU_acti_0 (Actinobacteria), where this angle constitutes from 65.0 to 66.8 degrees (65.8 degrees in average).

Angle between alpha helixes 2 of two HU subunits that form a homodimer constitutes from 82.0 to 85.0 degrees (83.5 degrees in average) and is equal for all the HU clades analyzed without exceptions.

Distance between C-terminal amino acid residue of alpha helix 2 and corresponding amino acid of the second HU monomer within a homodimer (Fig 5) can be used as an estimation of the HU dimer alpha-helical core size. It constitutes from 3.28 to 3.39nm (3.33 nm in average) for all the 3D-models built, without any exception.

HU arms are flexible and are able to adopt DNA minor grove independently of DNA sequence. HU has a capacity to bind DNA not only in B-form, but also dsRNA and RNA-DNA hybrids in A-form [14]. HU arms are mainly composed from anti-parallel beta-sheets. Both distances between the ends of beta strands 2 of opposite monomers and the starts of beta strands 5 of opposite monomers define the width of DNA–adopting platform. For each model we calculated distances between residues corresponding two C-termini of beta strands 2 of opposite monomers and between their neighboring residues as well as distances between the residues corresponding the N-termini of beta strands 5 of opposite monomers and between their neighboring residues (Fig 5).

For the beta strands 2 ends, the distances between three corresponding C-alpha atoms of consecutive residues 54, 55, 56 (numeration is according to Fig 2) are 2.48, 2.9, and 2.64 nm in average. For the beta strands 5 starts, the distances between three corresponding C-alpha atoms of consecutive residues 73, 74, 75 are 3.12, 2.67, and 3.42 nm, respectively, in average. For all modeled HU these distances are essentially same, with deviations less than 0.15 nm.

Although, among a hundred of HU models built we found only several sequences with deviations in DNA binding platform parameters of HU. For example, for HU from *Propionibacterium acnes*, distance between C-alpha atoms of beta strands 2 C-termini of HU from *Propionibacterium* acnes constitutes 2.63 nm in average and distances between the subsequent residues are also deviated to 3.21 nm in average.

**Fig 8:**
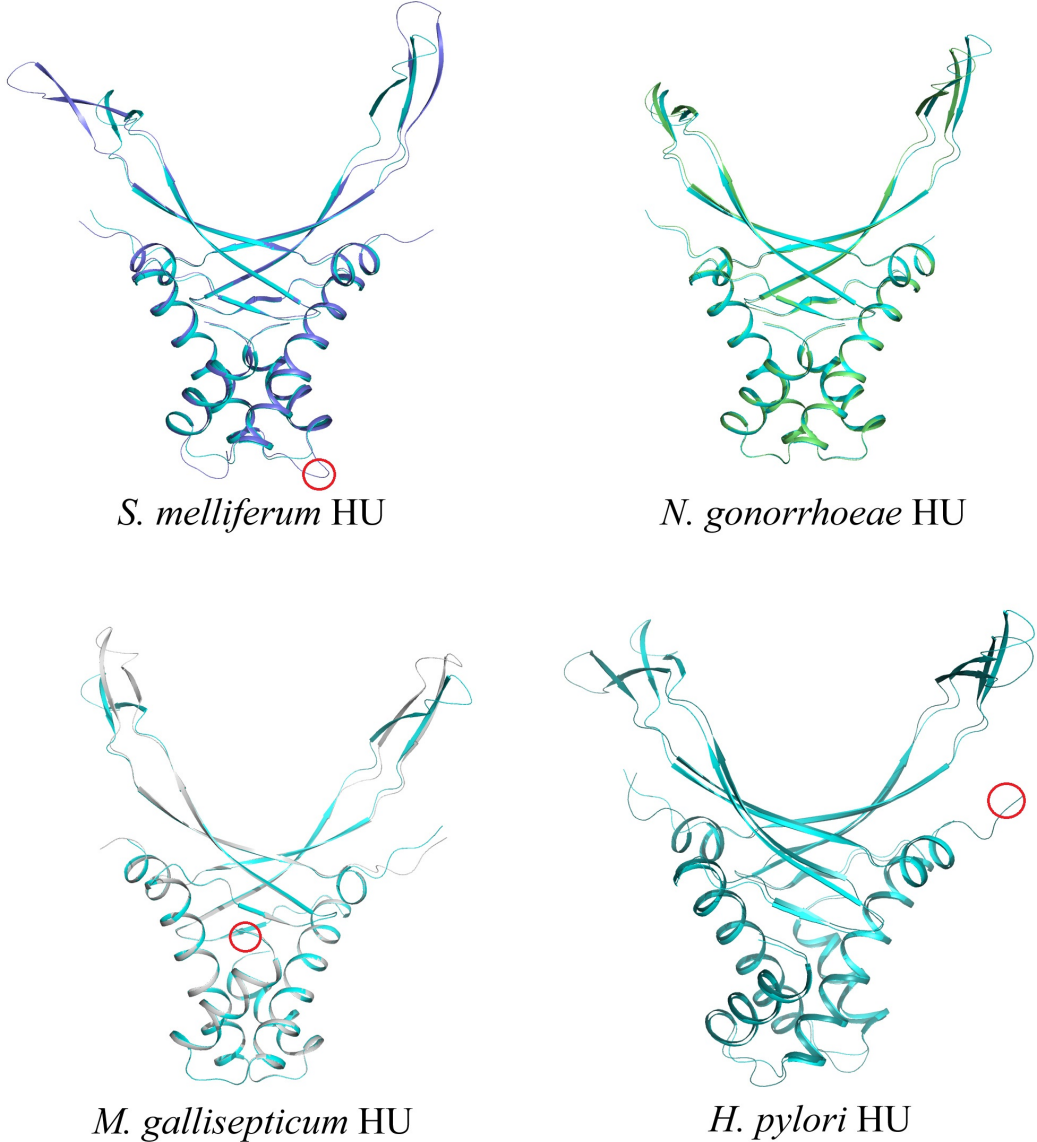
Models of *S*. *melliferum* (blue), *N*. *gonorrhoeae* (green), *M*. *gallisepticum* (grey), *and H*. *pylori* HU (deep teal) superimposed with *E*. *coli* HUα model (cyan). Two-residue insertion between alpha helix 1 and 2 in *S*. *melliferum* HU; six amino acid long extension at the N-termini of *M*. *gallisepticum* HU and four amino acid long extension at the C-termini of *H*. *pylori* HU are indicated with red circles.

Thus, differences in folding of both DNA–binding platform and HU alpha-helical body remains insignificant comparing to essentially different HU sequences. We conclude that HU fold parameters are even more conservative than HU protein sequence. It is interesting, that amino acid insertions and deletions in 90 amino acid HU core sequence as well extensions at the N- and C-termini of the protein do not cause significant deviations of 3D structure of HU dimers. Fig 8 shows how models of four HU proteins (three with either insertion or short N- or C- extensions of core sequence) superimposed with *E*. *coli* HUα model; DNA-binding properties of these proteins are described below.

### HU dsDNA binding site size on the dsDNA

Finally, we compared DNA-binding features of four HU representatives from three different HU clades related to each other either closely or distantly. For the comparison, we choose already well-known *E*.*coli* HUα that belongs to HU clade HU_ecoA and three HU proteins with previously unknown DNA-binding properties including one protein from *N*. *gonorrhoeae* and two mycoplasma HU, *S*. *melliferum* and *M*. *gallisepticum*.

Most populated clades of proteobacteria, HU_ecoA and HU_ecoB, contain similar proteins(Figs 1A and 3). *E*.*coli* HUβ entitles the HU_ecoB clade. Majority of the HU_ecoB proteins are homodimers, while *E*.*coli* HUβ makes heterodimer with HUα protein; *E*.*coli* HUβ-HUβ homodimer is unstable at low HU concentration [24,45]. *N*. *gonorrhoeae* HU belongs to HU_ecoB clade; being the only HU in *N*. *gonorrhoeae* it functions as a homodimer. *N*. *gonorrhoeae* HU has 51 and 61 identities with *E*.*coli* HUα and HUβ, respectively. Thus, *N*. *gonorrhoeae* HU protein was taken as a typical member of HU_ecoB clade to study its DNA-binding properties.

High genome plasticity of Mollicutes [40] leads to high diversity of protein sequences, including HU: *S*. *melliferum* HU belongs to the most populated HU_ecoB clade (S3 Table). It has only 38 identities with both *E*.*coli* HUα and HUβ, and 39 identities with *N*. *gonorrhoeae* HU. *S*. *melliferum* HU has increased number of phenylalanine residues, which enhances hydrophobic interactions in its dimeric interface [38], and a two-residues insertion within the loop between alpha helixes 1 and 2 (Fig 8). *M*. *gallisepticum* HU has from 21 to 23 identical residues with both E.coli HUs, *N*. *gonorrhoeae* HU and *S*. *melliferum* HU. In addition to the lowest similarity in the 90 amino acid HU core, it has six amino acid residues long N-terminal extension and amino acid substitutions in the most conservative regions (e.g. dimerization signal (DS) and DNA-recognizing tips) [8,41].

**Fig 9:**
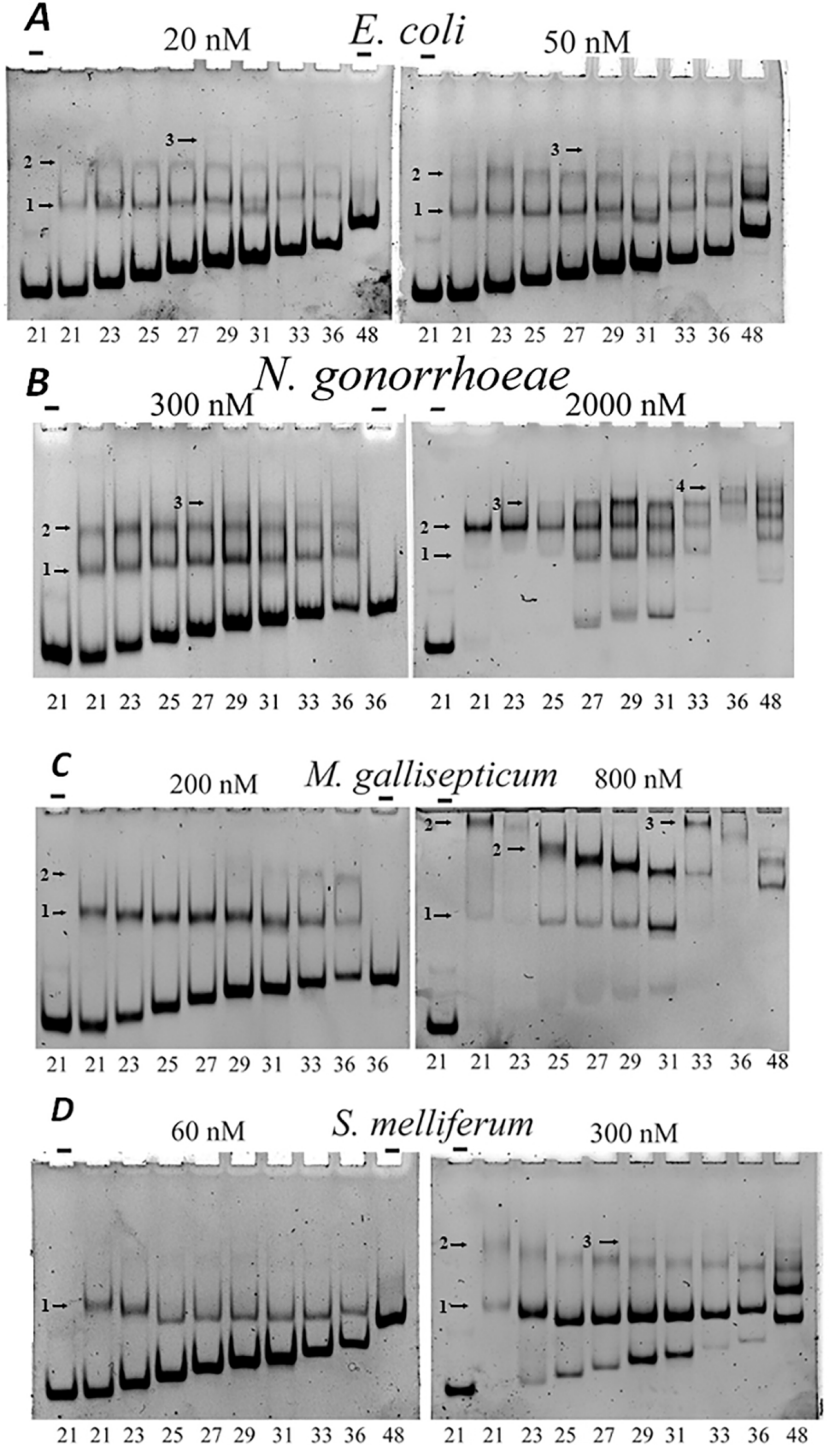
HU binding to dsDNA of various lengths. Binding of labeled DNA to HU proteins was analyzed by polyacrylamide gel electrophoresis. The gel was buffered with 50 mM Tris–borate; binding mixture contains 40 mM NaCl. DNA samples were: dsDNA of sequence ‘D’ with the length varying from 21 to 48 bp (indicated at the bottom). HU origin and concentration is indicated at the top (“-“, no HU was added). Bands corresponding to HU-DNA complexes are marked with arrows, the number of HU dimers in each complex is indicated on the left of the arrow. Panels correspond to HU proteins of various bacteria, protein concentrations are indicated.

The size of the HU binding site was estimated by measuring its binding to dsDNA substrates of various lengths. Binding of HU proteins to canonical dsDNA is considered nonspecific because it is weak at physiological and high salt concentrations [12,14,33]. Therefore, the gel mobility shift assay with HU−dsDNA complexes was conducted at low salt concentrations (40 mM NaCl and 50 mM Tris-borate buffered gel). The results are shown in the Fig 9. *E*. *coli* HUα binding to dsDNA is noticeable for all tested DNA lengths, from 21 to 36 bp (Fig 9A). Two protein-DNA complexes can be detected for these DNA lengths, and a third complex is visible for DNA lengths from 29 bp, especially at higher HU concentration. Therefore, DNA length increase at 9–10 bp allows one more HU complex to be added to DNA molecule. We conclude that dsDNA binding site size of *E*.*coli* HUα is 9–10 bp in agreement with previous estimates [33].

Similarly, for *N*. *gonorrhoeae* HU, two protein-DNA complexes can be detected for DNA lengths from 21 to 48 bp, and a third complex is visible for DNA lengths from 27 to 48 bp, at higher HU concentration; four complexes can be detected for 36 and 48 bp dsDNA (Fig 9B). So, one HU complex is added when DNA length increases at 9 bp. We conclude that dsDNA binding site size of *N*. *gonorrhoeae* HU is 9 bp.

*M*. *gallisepticum* HU forms two protein-DNA complexes when DNA is 21 bp or longer (Fig 9C, 800 nM of protein). Complex 2 has abnormally low mobility for shorter DNA (see below). Third complex is visible for DNA lengths of 33 and 36 bp at higher HU concentration (again, with abnormally low mobility) (Fig 9C). So, one HU complex is added when DNA length increases at 11–12 bp. Taken together, these results suggest that dsDNA binding site size of *M*. *gallisepticum* HU is 11 bp.

*S*. *melliferum* HU forms two protein-DNA complexes when DNA is 21 bp or longer. Third complex is visible for DNA lengths starting at 29 bp (Fig 9D). We conclude that dsDNA binding site size of *S*. *melliferum* HU is 9–10 bp.

### HU binding cooperativity

It is clearly seen in the Fig 9D that for higher HU concentration complex 1 formed by *S*. *melliferum* HU with dsDNA is more presented than complex 2, and free DNA (see corresponding band intensities in Fig 9D).

It suggests that binding of the second HU dimer to DNA molecule is decreased when one HU dimer is already bound to this DNA molecule. Cooperativity parameter(ω) specifying the relative affinity of the second bound HU dimer for a contiguous site versus an isolated binding site, ω, can be calculated from the cooperative McGhee–von Hippel equation. Cooperativity parameter for *S*. *melliferum* HU was estimated as ω = 0.25±0.05. Thus, binding of the second HU *S*. *melliferum* dimer is four times weaker than binding of the first dimer. In contrast, *E*.*coli* HUα binds dsDNA in moderately cooperative manner: binding of the second HU dimer to DNA fragment is 10 folds stronger than the binding of the first dimer (Fig 9A). This result is in agreement with previous findings that *E*. *coli* HUα is characterized by cooperative multimer binding on dsDNA longer than the minimal binding site [33]. Similar binding cooperativity (ω = 12±3) was obtained for *N*. *gonorrhoeae* (Fig 9B). *M*. *gallisepticum* HU also binds dsDNA in a cooperative manner–binding of the second *M*. *gallisepticum* HU dimer to dsDNA fragment is 20 fold stronger than the binding of the first dimer (Fig 9C). Coperative binding to DNA was detected also for *H*. *pylori* HU from HU_Helicobacter clade (ω = 60 [46]). Non-cooperative binding of *S*. *melliferum* HU to DNA is similar *to A*. *laidlawii* HU (clade HU_Firmicutes) binding [47]. Non-cooperative binding of HU could lead to more uniform distribution of HU along bacterial chromosome while cooperative binding may cause the formation of DNA stretches covered by HU.

### Comparison of DNA-protein complex gel mobility

Free DNA mobility in the gel decreases with DNA length (Fig 9). HU-DNA complex mobility also decreases with DNA length for *N*. *gonorrhoeae* and *E*. *coli* HUs (Fig 9A and 9B). It is not the case for *M*. *gallisepticum* and *S*. *melliferum* -HU complexes with dsDNA (Fig 9C and 9D). Explanation of this abnormal mobility of HU-DNA complexes: HU bends dsDNA and complex with longer DNA fragment is more compact than with the shorter one. For *E*. *coli* HU such effect was already studied by the comparison of HU-dsDNA and HU-nick DNA complex nobilities [12]. Nicked DNA is more flexible and *E*. *coli* HU introduces a kink into DNA. We believe that *S*. *melliferum* and *M*. *gallisepticum* HUs bend dsDNA stronger than *E*. *coli* or *N*. *gonorrhoeae* HU proteins as maximum mobility’s of HU–DNA complexes correspond to 31, 25, and 25 bp for *E*. *coli* HUα, *S*. *melliferum* and *M*. *gallisepticum* HUs, respectively. *N*. *gonorrhoeae* HU–dsDNA complex mobility is similar to *E*. *coli* HU.

### Specific binding of HU

**Fig 10:**
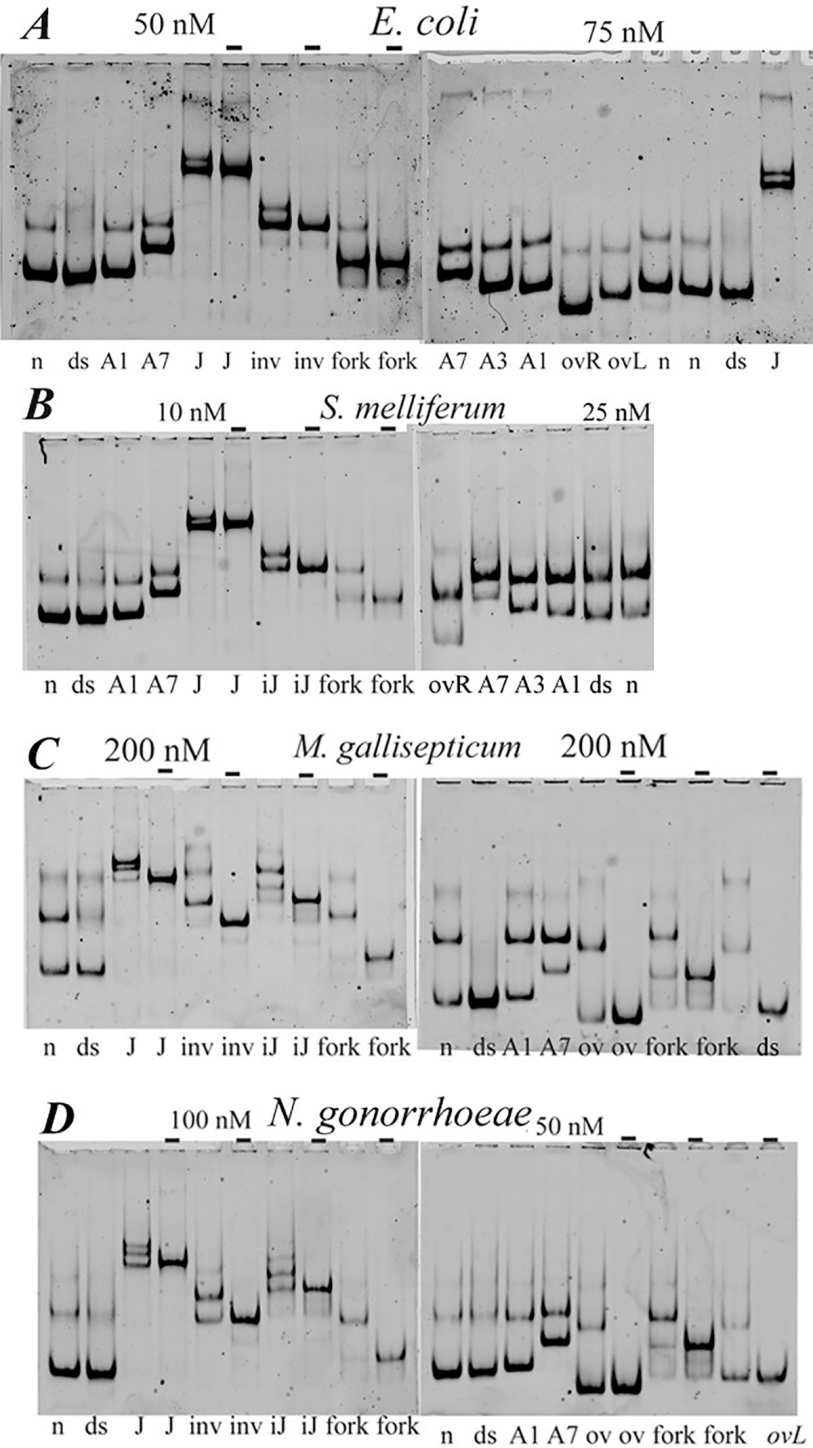
HU binding to “distorted” DNA structures checked by polyacrylamide gel mobility assay. HU protein at concentrations indicated above the gel image (“-“, no HU was added) was mixed with 5’-labelled DNA in a buffer containing 150 mM NaCl; the bound and free DNA were gel-separated. DNA structures indicated at the bottom of the gel images: n, nicked DNA; ds, dsDNA; A1, A3 and A7, DNA bulges, containing one, three or seven non-paired adenines in one of DNA strands; J–four-way junction; fork, ssDNA fork; ov, DNA overhang; iJ, incomplete junction lacking one DNA strand; inv, DNA invasion. Panels correspond to HU proteins of various bacteria.

HU-binding targets are not limited to generic dsDNA. *E*. *coli* HU binds with much higher affinity to distorted DNA structures such as forks, three/four-way junctions, nicks and overhangs [12,16,34,35]. We constructed some of these structures using labeled oligonucleotides. Ability of HU from four bacterial species to bind DNA constructs was estimated by electrophoretic mobility shift assay, EMSA (Fig 10). To discern non-specific dsDNA binding from the structure-specific binding to distorted DNA structures, EMSA experiments were conducted at higher Tris-Borate concentration and binding was tested at “physiological” salt concentration, 150 mM NaCl.

As can be seen in Fig 10A, nicked DNA (n), DNA bulges (linear DNA with 1 or 3 or 7 adenines inserted in one DNA strand, marked as A1, 3 or 7), DNA fork (f, Y-shape structure) as well as DNA overhang, junctions and invasion form single complexes with *E*. *coli* HUα under high salt conditions, which consequently migrate in the gel as sharp bands. In contrast, the non-specific complexes formed with linear DNA result in the appearance of a smear, since the salt-sensitive complexes partially dissociate during their migration in polyacrylamide gel (Fig 10A–10D).

**Fig 11:**
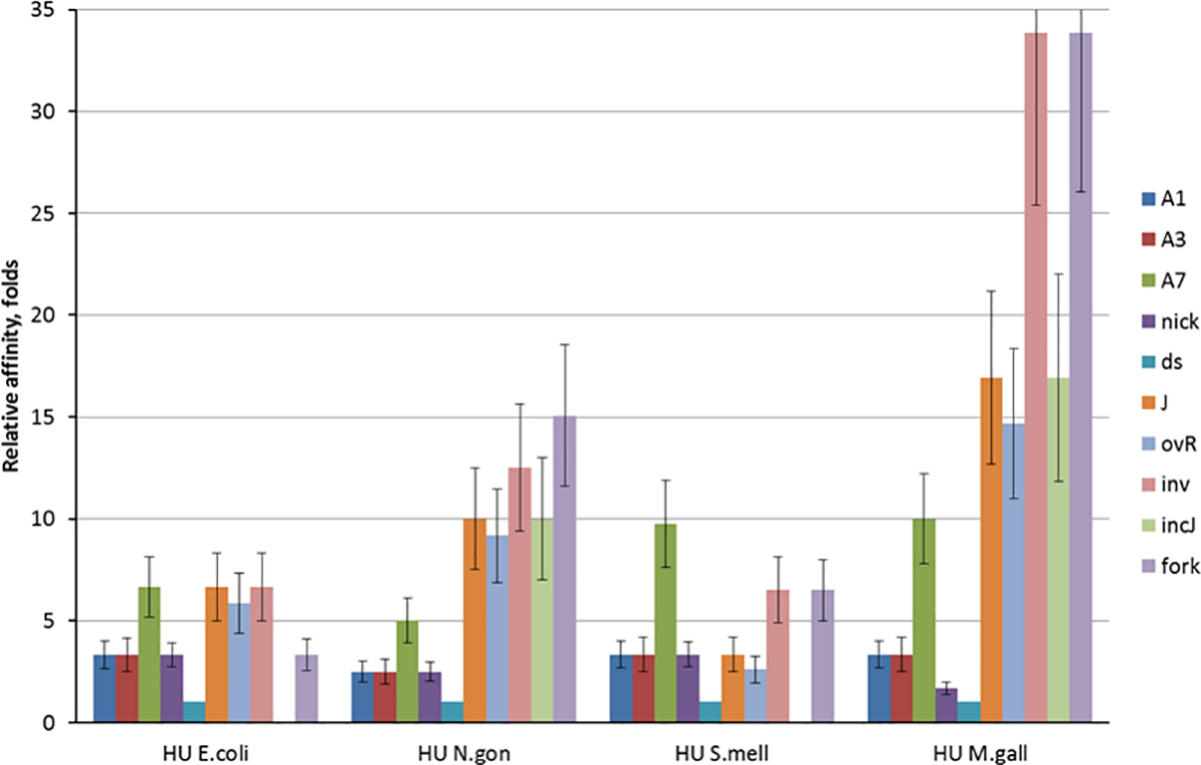
The profiles of HU affinities to various DNA-structures. Each column represents the association constant of HU complex with one DNA structure (see Fig 10 legend for structures description) normalized on the association constants of HU-dsDNA complex of the same protein. Data from at least three independent experiments were combined.

To determine the affinities of each HU protein to different DNA-structures, the dissociation constants (Kd) of observed complexes and its reciprocal—the association constants were calculated. The Kd values are available from supporting materials (S5 Table). The association constants of HU complexes with various DNA structures normalized on the association constants of HU-dsDNA complexes are shown in (Fig 11).

Comparison of individual profiles of specificity indicates that all HU proteins has lowest affinity to small A1 and A3 bulges and nicked DNA and high affinity to A7 bulge (especially for *S*. *melliferum* HU), DNA junctions and DNA overhang (with the exception of *S*. *melliferum* HU), DNA invasion and DNA fork (the latter, with an exception for *E*.*coli* HUα). DNA junctions and DNA invasion as well as fork and A7 bulge (in lesser degree) are more preferable HU substrates, perhaps as they carry more sites for HU binding than smaller DNA structures. At the same time, all studied HU proteins have individual characteristics of DNA binding. *E*. *coli* HUα has relatively low level of discrimination between the DNA-substrates comparing to other three proteins. *S*. *melliferum* HU has highest affinity to A7 bulge DNA and similar affinities to nick, junction and overhang DNAs, while affinity *M*. *gallisepticum* HU to nicked DNA is about 10 and 20 times less than to DNA junctions and invasion, respectively. *N*. *gonorrhoeae* HU also binds DNA junction and invasion 4 and 5 times stronger than nick. Such better recognition of DNA junction compared to nick was already shown for *H*. *pylori* HU [46].

#### Conclusion

High-scale alignment and clusterization of thousands histone-like proteins coupled with comparative analysis of hundred 3D fold models corresponding to maximal variations of HU sequences and investigation of DNA-binding properties of limited set of HU proteins including three previously not characterized HUs showed that 1) according to primary structure each representative of HU/IHF protein family (InterPro ID IPR000119) can be unambiguously attributed to one of three group: HU, IHF_A or IHF_B; 2) HU proteins 3D folding is more conservative than HU sequence; 3) comparison of DNA-binding features of four HU representatives closely or distantly related to each other show that in respect to DNA recognition, each HU protein has its individual profile of affinities to various DNA-structures with the increased specificity to the most complex structures. At the same time, the most dissimilar mycoplasma’ HUs bend dsDNA stronger than *E*.*coli* and *N*. *gonorrhea* HUs and have other deviations such as non-cooperative binding of *S*. *melliferum* HU and large DNA-binding site size of *M*. *gallisepticum* HU on dsDNA. Thus, the diversity in DNA recognition and bending features of HU proteins described here correlates with high variabilities of their sequences though not always coincides with proposed HU clusterization. This finding indicate that application of more sophisticated molecular-dynamics approaches for the broader set of HU proteins would be useful for investigation of structural basis of functional variability of HU-like proteins We hope that our work will help researchers to itemize any particular HU or IHF protein of interest.

## Supporting information

S1 File Sequence identification and analysis details. (DOCX)

S2 File Model validation details. (DOCX)

S3 File Models of HU proteins. (ZIP)

S4 File Model validation. (ZIP)

S1 Table Oligonucleotides used in this study for E.coli and N. gonorrhoeae HU genes cloning and for DNA construction. (XLSX)

S2 Table Score matrixes for HU/IHF clades. (XLSX)

S5 File Multiple sequence alignment and clusterization results description of S3 Table. (DOCX)

S3 Table Multiple sequence alignment and clusterization of HU/IHF family protein sequences. (XLSX)

S4 Table Sequences of HU proteins used for comparative modeling. (XLSX)

S5 Table Dissociation constants. (XLSX)
