# Supplementary material for: Comparison of histone-like HU protein DNA-binding properties and HU/IHF protein sequence alignment
Source: PLoS One. 2017 Nov 13;12(11):e0188037. doi: 10.1371/journal.pone.0188037 (PMC5683647; doi:10.1371/journal.pone.0188037)
Supplement: S1 File — (DOCX) [file pone.0188037.s001.docx]

**Supporting information S1 file**

**Sequence identification and analysis details**

Members of HU/IHF family proteins were identified by InterPro ID IPR000119, which represents bacterial histone-like proteins. Multiple sequence alignment (MSA) was performed by the rate matrix of residue substitution search.

The standard way to perform multiple sequence alignment (MSA) is to employ CLUSTAL tool, available in:

EMBL, https://www.ebi.ac.uk/Tools/msa/clustalo/

Expasy, https://www.expasy.org/genomics/sequence_alignment

UniProt , <http://www.uniprot.org/align/>

and many other bioinformatic consortiums sites.

Use CLUSTAL is the good way to perform MSA, especially, de novo. Here, we employed CLUSTAL for MSA of HU/IHF family proteins sequences and to verify results obtained by another approach.

Dozens of HU/IHF family proteins are experimentally proved to be histone-like proteins (see refs in reviews [22, 36]). These proteins can be used as the initial motifs to align other HU/IHF family proteins sequences. Thus, we used rate matrix of residue substitution search to perform MSA along with CLUSTAL.

In short,

1. Score matrix is used to find a score of any protein sequence and to find the position of the motif within this sequence. Score matrix is built on the basis of several already aligned sequences. It contains 20 rows, corresponding to each amino acid and 90 columns, corresponding to each position in HU protein core, which is 90 – residues long.

Final score matrixes for each found HU and IHF clades are represented in S2 Table.

Each cell (x, y) of score matrix corresponding to the analyzed clade is equal to

log2(number of corresponding amino acid (x) in the clade at position y/ number of sequences in the clade),

if number is equal to zero, or calculated value is less than -7, then cell (x, y) = -7.

1. Each sequence of HU/IHF family proteins identified by InterPro ID IPR000119, which represents bacterial histone-like proteins, is analyzed with score matrix, starting from position 1, 2, etc., in the protein sequence. Sum of 90 score matrix cells corresponding to amino acids in the analyzed sequence provides a sequence score. Sequence position with the best score provides the start of the HU core in the analyzed sequence.

To estimate if the analyzed sequence contains one or more inserted amino acid residues, in the score matrix one or more columns with zero cells was inserted. Each sequence of HU/IHF family proteins was analyzed for 1 to 8 – long insertions and 1 or 2 residue deletions at positions from 8 till 72 in 90 residue long core HU sequence. Best score was assessed (gap penalties were taken into account) to decide if analyzed sequence contains insertions or deletions as well as its position.

Comparison of the analyzed sequence scores for each clade reveals to which clade belongs the sequence.

1. To partition one clade into two clades, we used CLUSTAL phylogenetic tree, UniProt Consortium tools[[1](#_ENREF_1)], or searched for the amino acid correlations in the sequences which belongs to the clade.

Thus, MSA/clusterization algorithm employed for HU/IHF family proteins sequences included three steps above: 1). To build score matrixes for the clades; 2). To find for each sequence of HU/IHF family proteins to which clade it belongs (taking into account insertions/deletions); 3). To partition clades to two smaller clades. These procedures were repeats several dozens of times to obtain S3 Table for the sequence attribution and S2 Table for the score matrixes.
