# Supplementary material for: Comparison of histone-like HU protein DNA-binding properties and HU/IHF protein sequence alignment
Source: PLoS One. 2017 Nov 13;12(11):e0188037. doi: 10.1371/journal.pone.0188037 (PMC5683647; doi:10.1371/journal.pone.0188037)
Supplement: S2 File — (DOCX) [file pone.0188037.s002.docx]

**S2 file. Model validation details.**

Models of HU proteins are presented in S3 file, model validation are presented in S4 file for each model. Sequences of HU proteins used for comparative modeling presented in S3 File and their validation are listed in S4 Table

The qualities of the best homology models chosen using MolProbity validation tools (Vincent et al., 2010) were further validated with ProSa interactive web service (Sippl, 1993; Wiederstein & Sippl, 2007).

For each model z-score value was compared with the range of scores typically found for native proteins of similar size obtained using either X-ray or NMR (top panel), single residue energies were plotted as a function of amino acid sequence position (medium panel) and visualized using the molecule viewer Jmol (bottom panel). In the latest residues are colored from blue to red in the order of increasing residue energy.

Referencies

Sippl, M.J. (1993) Recognition of Errors in Three-Dimensional Structures of Proteins. Proteins 17, 355-362 and Wiederstein & Sippl (2007) ProSA-web: interactive web service for the recognition of errors in three-dimensional structures of proteins. Nucleic Acids Research 35, W407-W410

Vincent B. Chen, W. Bryan Arendall III, Jeffrey J. Headd, Daniel A. Keedy, Robert M. Immormino, Gary J. Kapral, Laura W. Murray, Jane S. Richardson and David C. Richardson (2010) MolProbity: all-atom structure validation for macromolecular crystallography. Acta Crystallographica D66: 12-21

Wiederstein & Sippl (2007) ProSA-web: interactive web service for the recognition of errors in three-dimensional structures of proteins. Nucleic Acids Research 35, W407-W410
