# Supplementary figures and images for: Comparison of histone-like HU protein DNA-binding properties and HU/IHF protein sequence alignment (part 2 of 2)
Source: PLoS One. 2017 Nov 13;12(11):e0188037. doi: 10.1371/journal.pone.0188037 (PMC5683647; doi:10.1371/journal.pone.0188037)

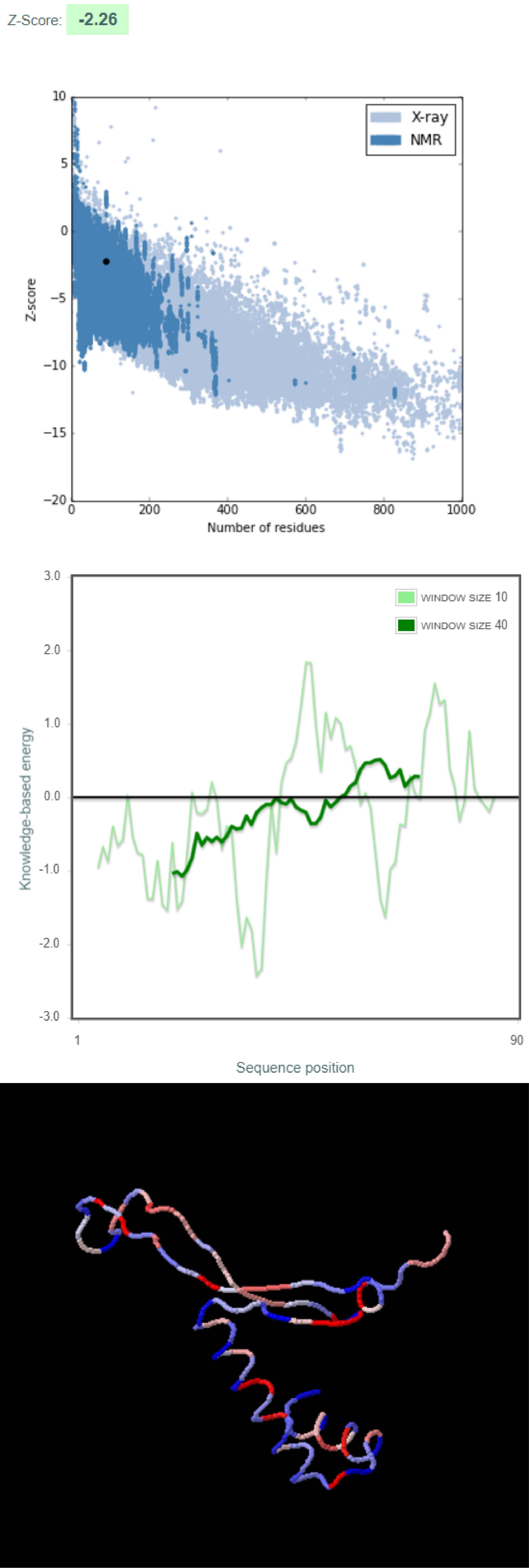

Supplement: S4 File — (ZIP) [file pone.0188037.s004.zip › E_4 v.jpg]

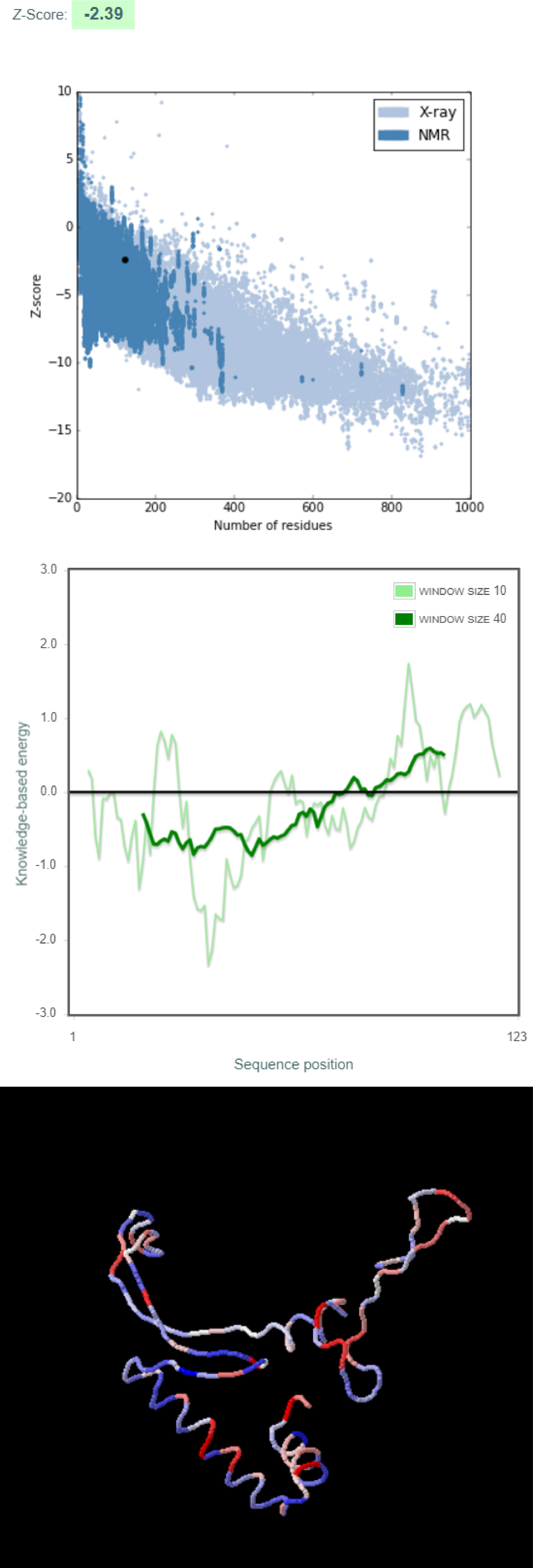

Supplement: S4 File — (ZIP) [file pone.0188037.s004.zip › M_1 v.jpg]

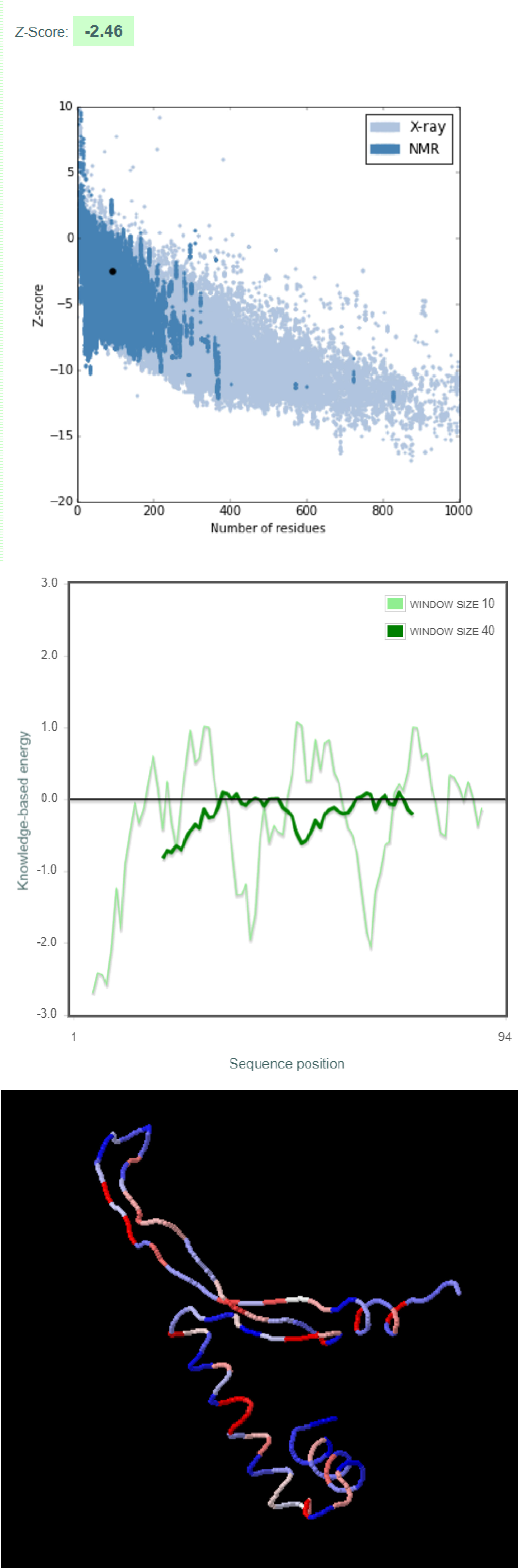

Supplement: S4 File — (ZIP) [file pone.0188037.s004.zip › S_1 v.jpg]
