# Supplementary figures and images for: Comparison of histone-like HU protein DNA-binding properties and HU/IHF protein sequence alignment (part 1 of 2)
Source: PLoS One. 2017 Nov 13;12(11):e0188037. doi: 10.1371/journal.pone.0188037 (PMC5683647; doi:10.1371/journal.pone.0188037)

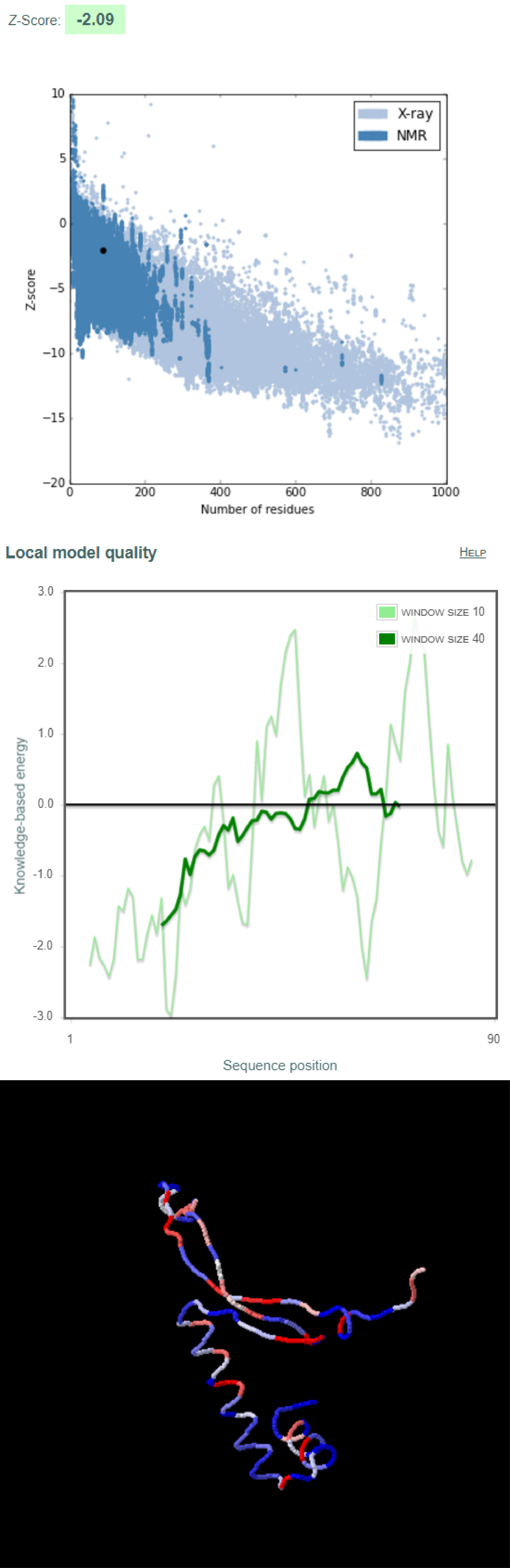

Supplement: S4 File — (ZIP) [file pone.0188037.s004.zip › A2_1 v.jpg]

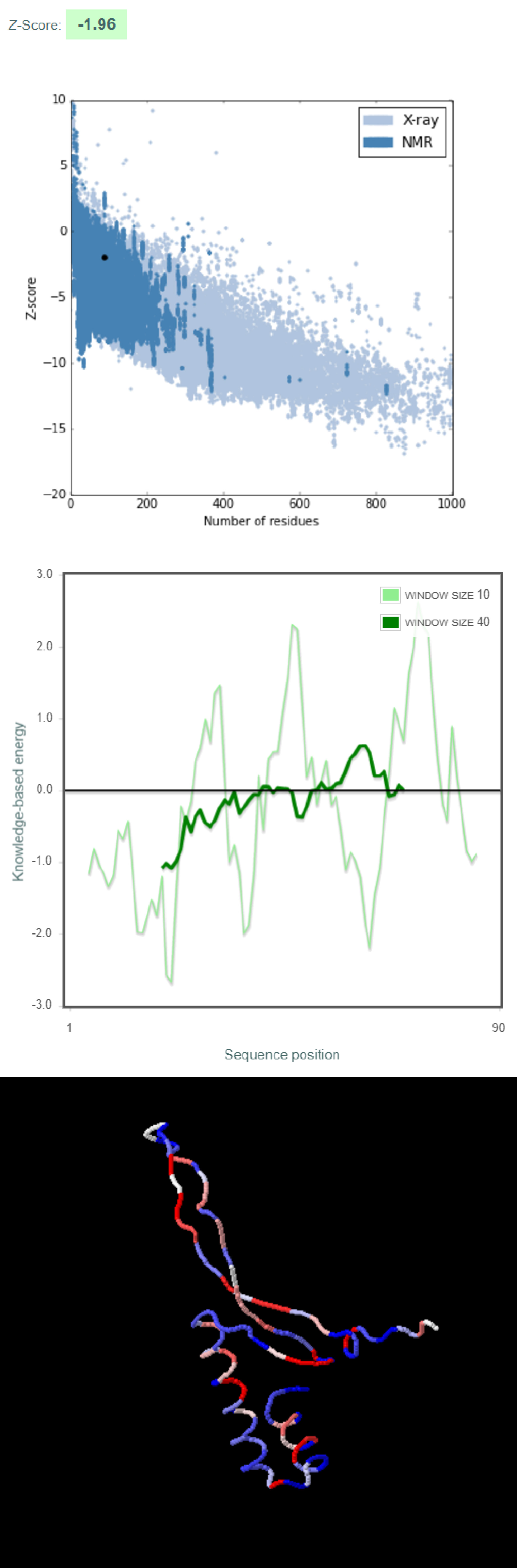

Supplement: S4 File — (ZIP) [file pone.0188037.s004.zip › A2_2 v.jpg]

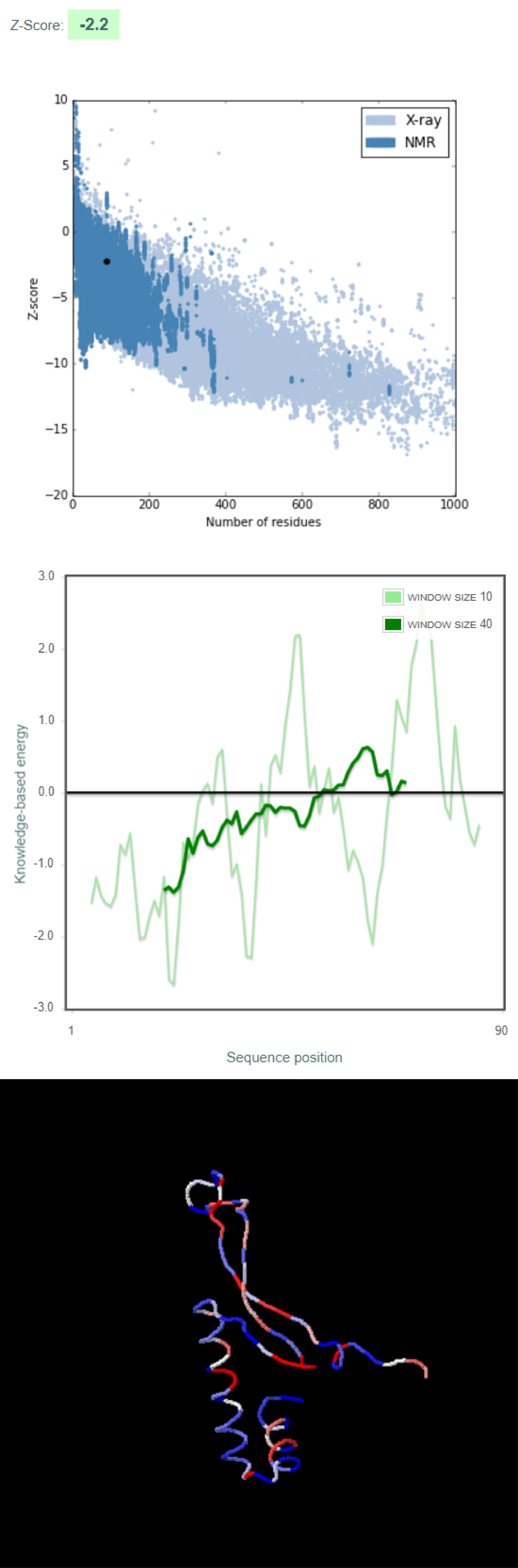

Supplement: S4 File — (ZIP) [file pone.0188037.s004.zip › A2_3 v.jpg]

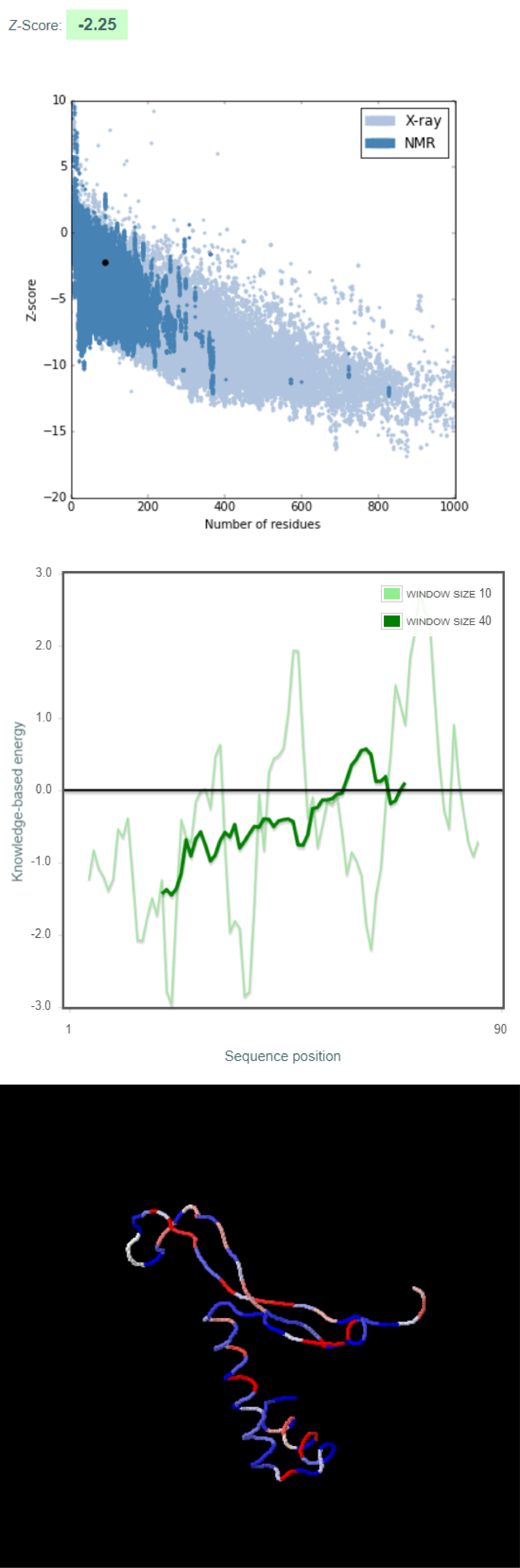

Supplement: S4 File — (ZIP) [file pone.0188037.s004.zip › A2_4 v.jpg]

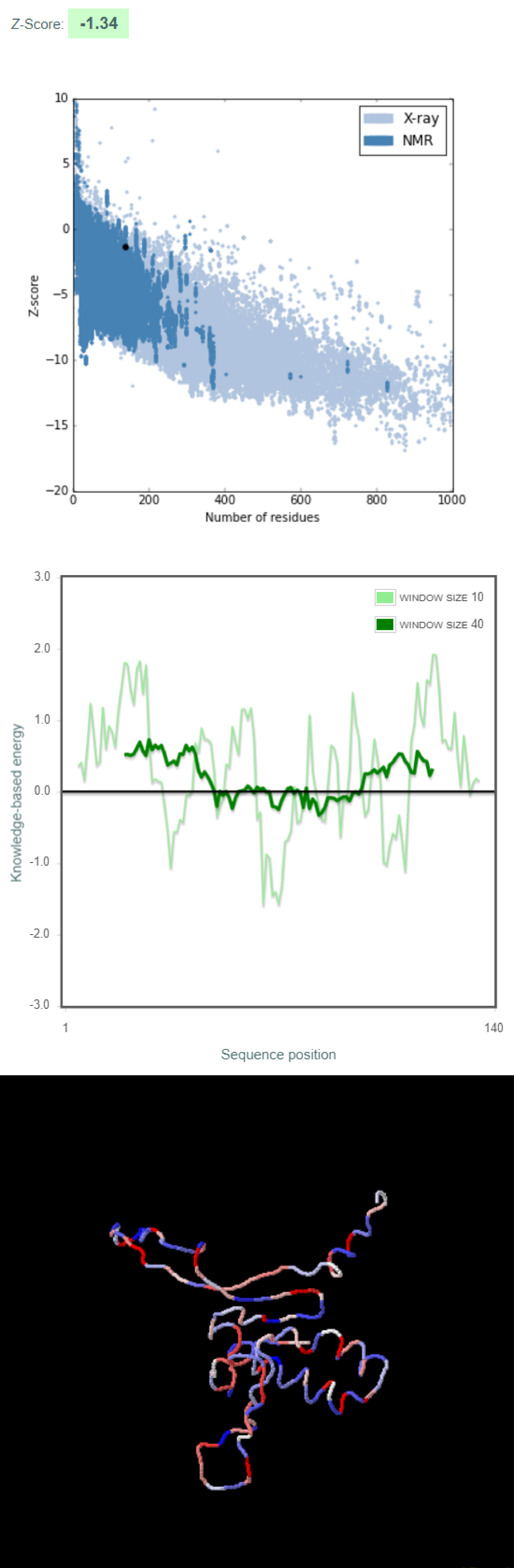

Supplement: S4 File — (ZIP) [file pone.0188037.s004.zip › A3_1 v.jpg]

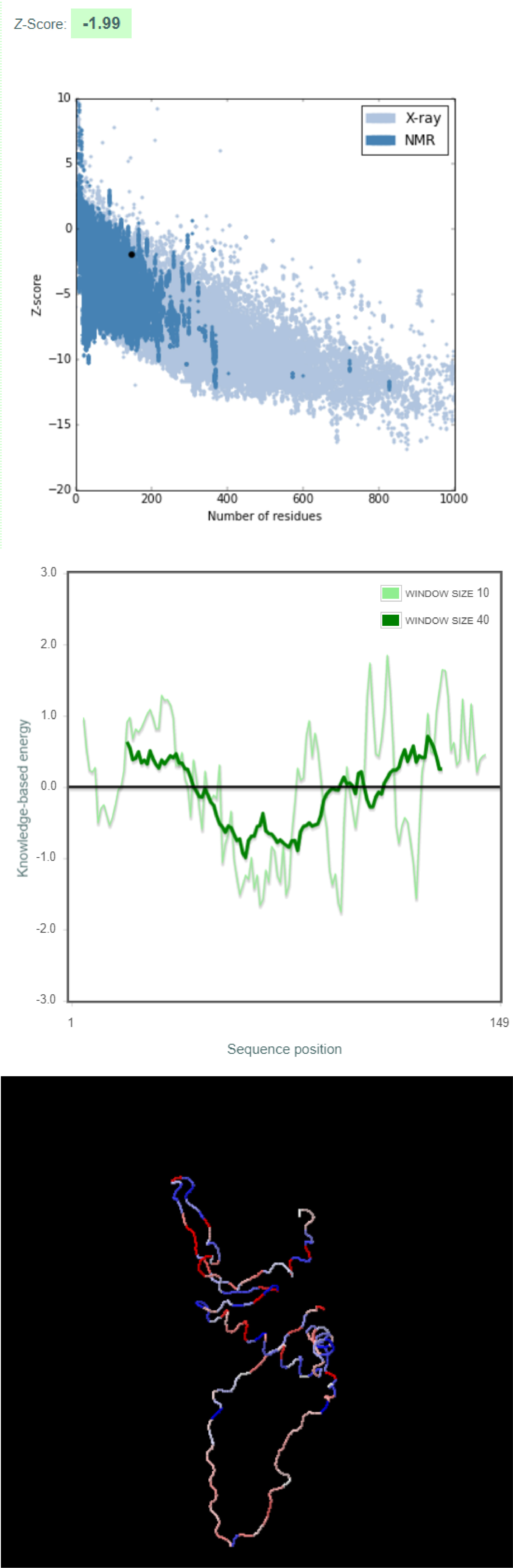

Supplement: S4 File — (ZIP) [file pone.0188037.s004.zip › A3_2 v.jpg]

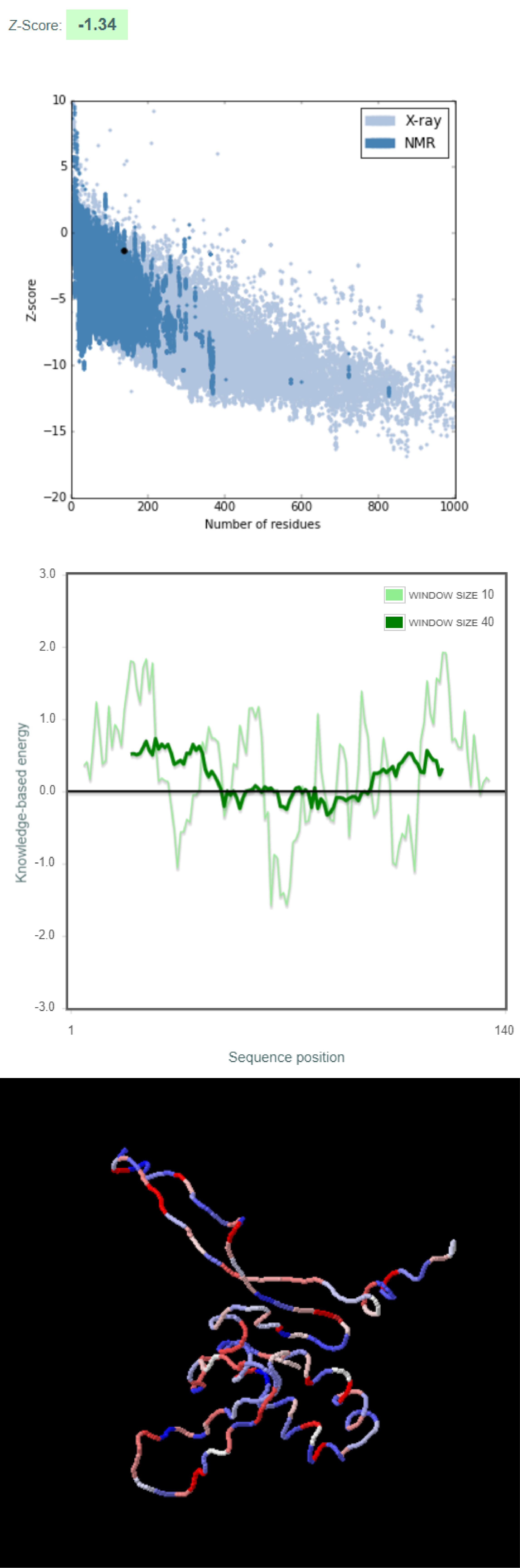

Supplement: S4 File — (ZIP) [file pone.0188037.s004.zip › A3_3 v.jpg]

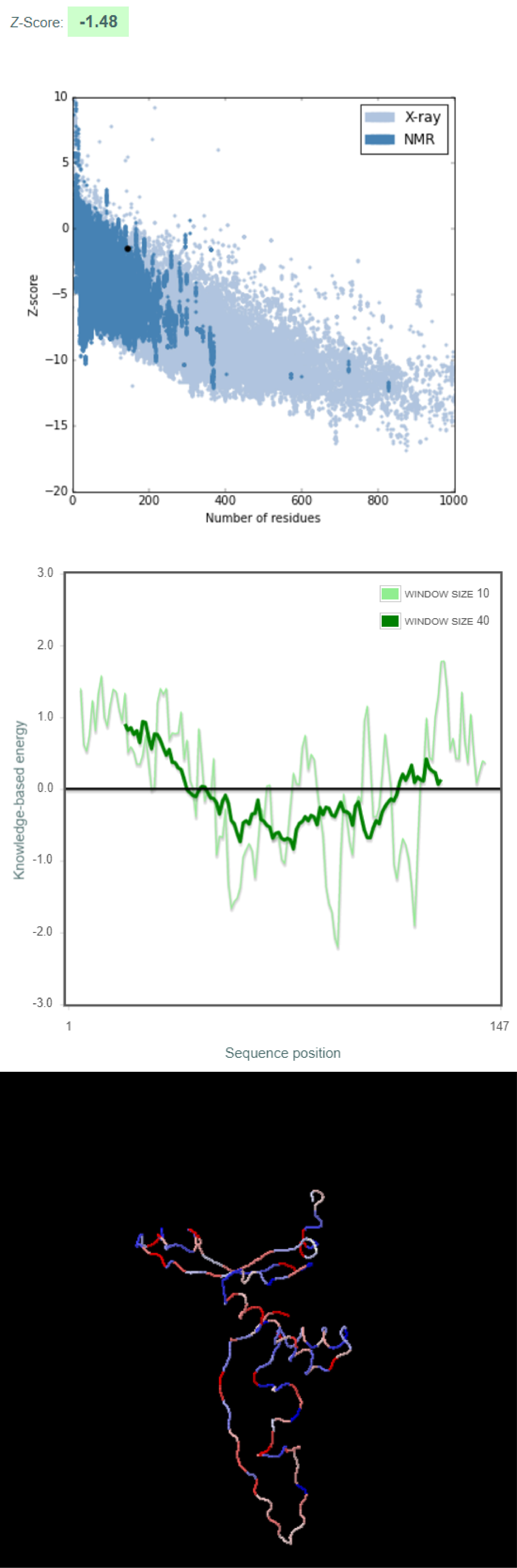

Supplement: S4 File — (ZIP) [file pone.0188037.s004.zip › A3_4 v.jpg]

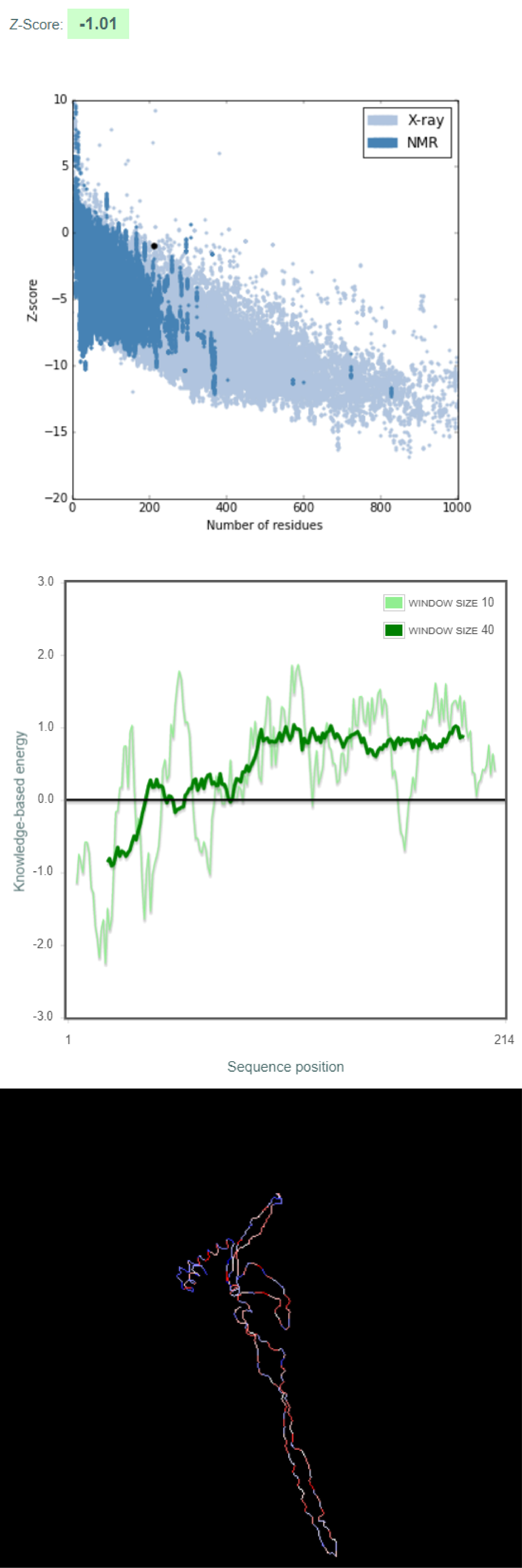

Supplement: S4 File — (ZIP) [file pone.0188037.s004.zip › A4_1 v.jpg]

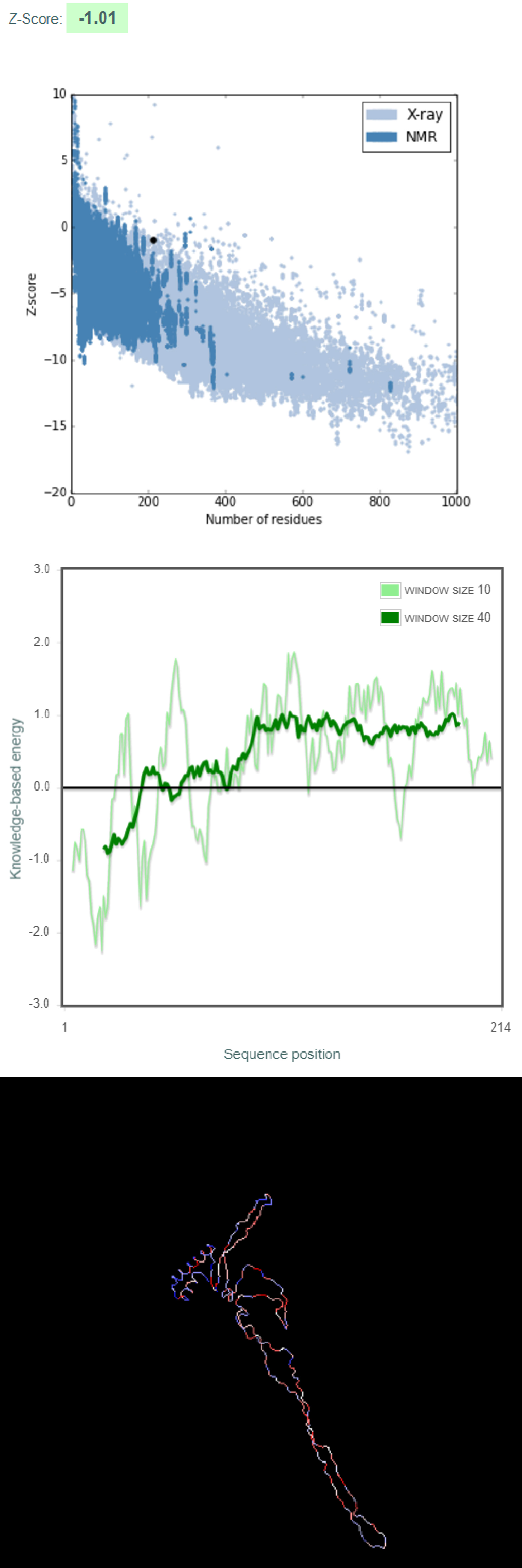

Supplement: S4 File — (ZIP) [file pone.0188037.s004.zip › A4_2 v.jpg]

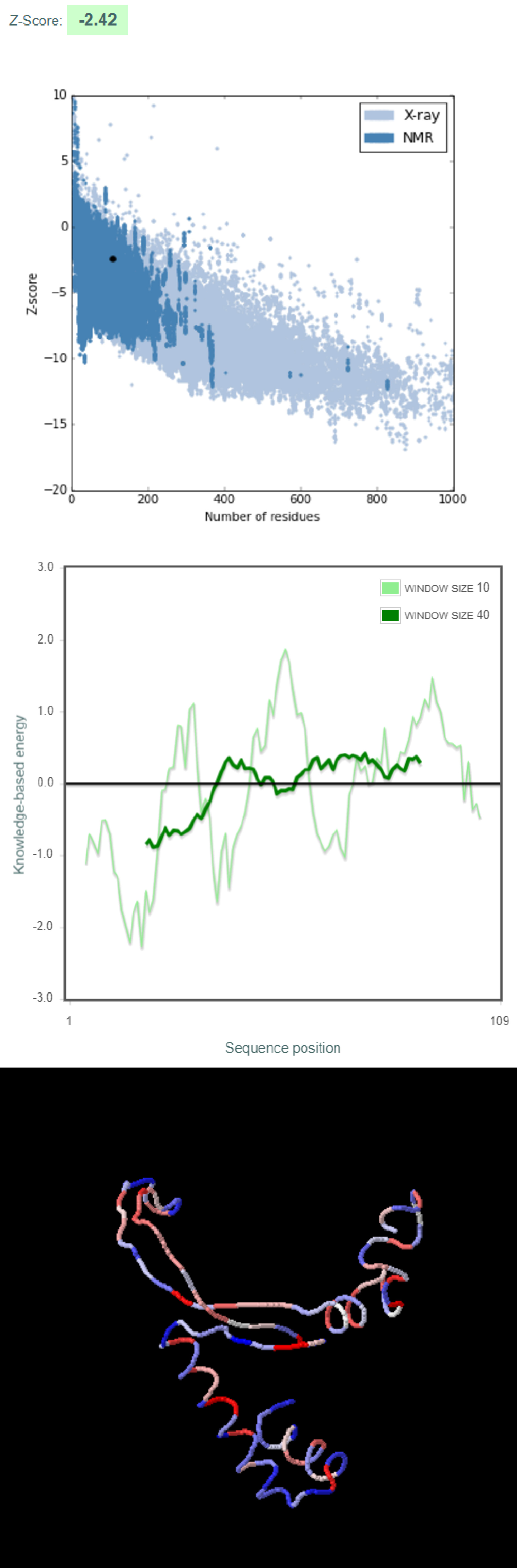

Supplement: S4 File — (ZIP) [file pone.0188037.s004.zip › A4_3 v.jpg]

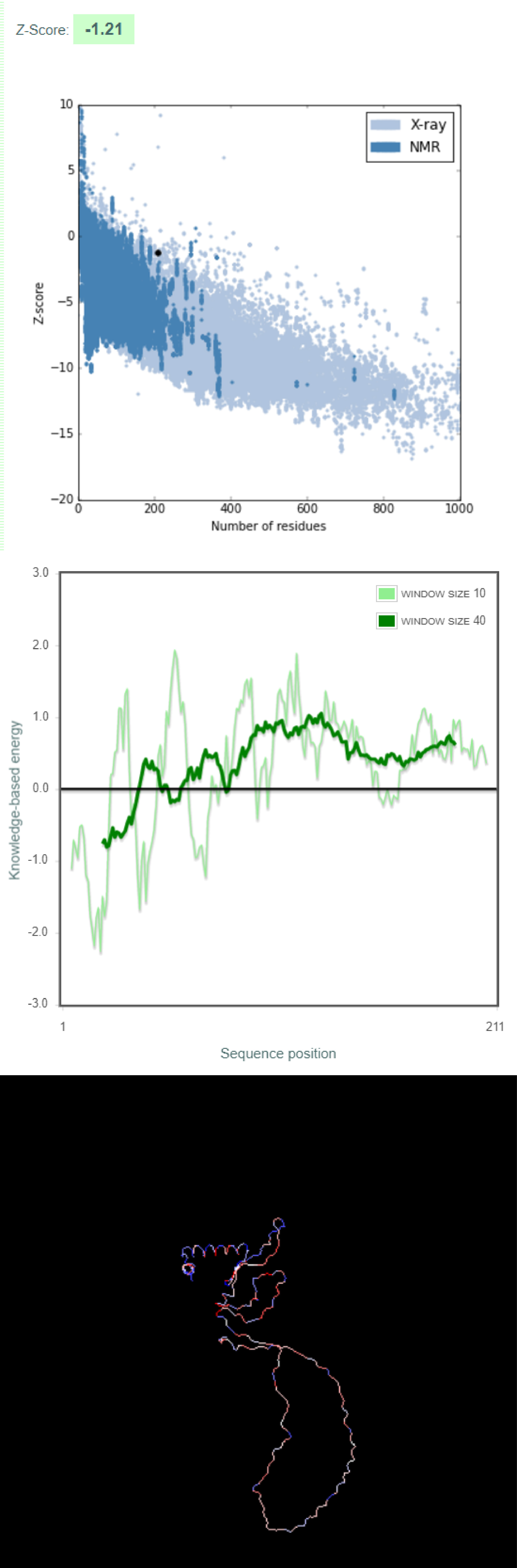

Supplement: S4 File — (ZIP) [file pone.0188037.s004.zip › A4_4 v.jpg]

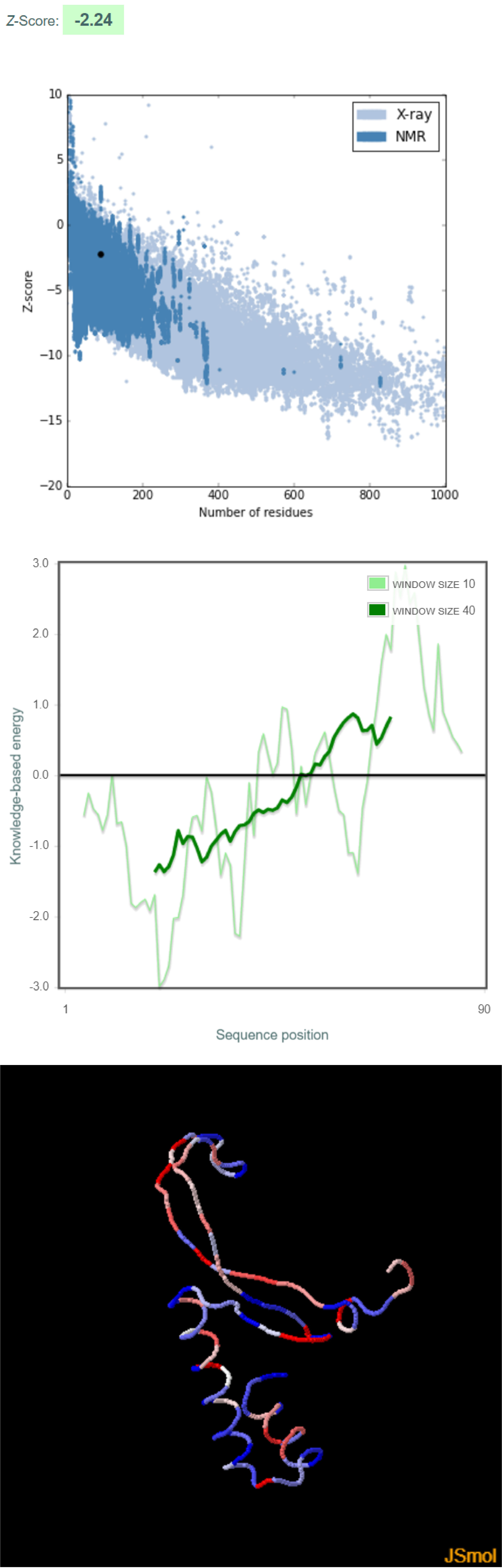

Supplement: S4 File — (ZIP) [file pone.0188037.s004.zip › A_1 v.jpg]

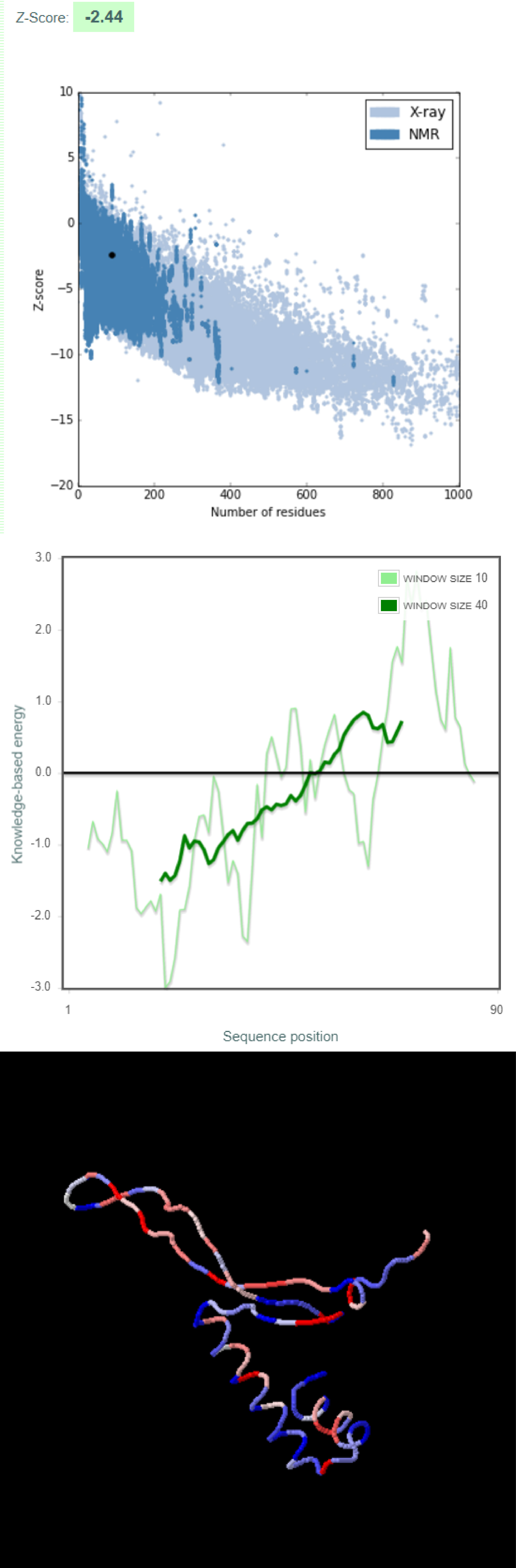

Supplement: S4 File — (ZIP) [file pone.0188037.s004.zip › A_2 v.jpg]

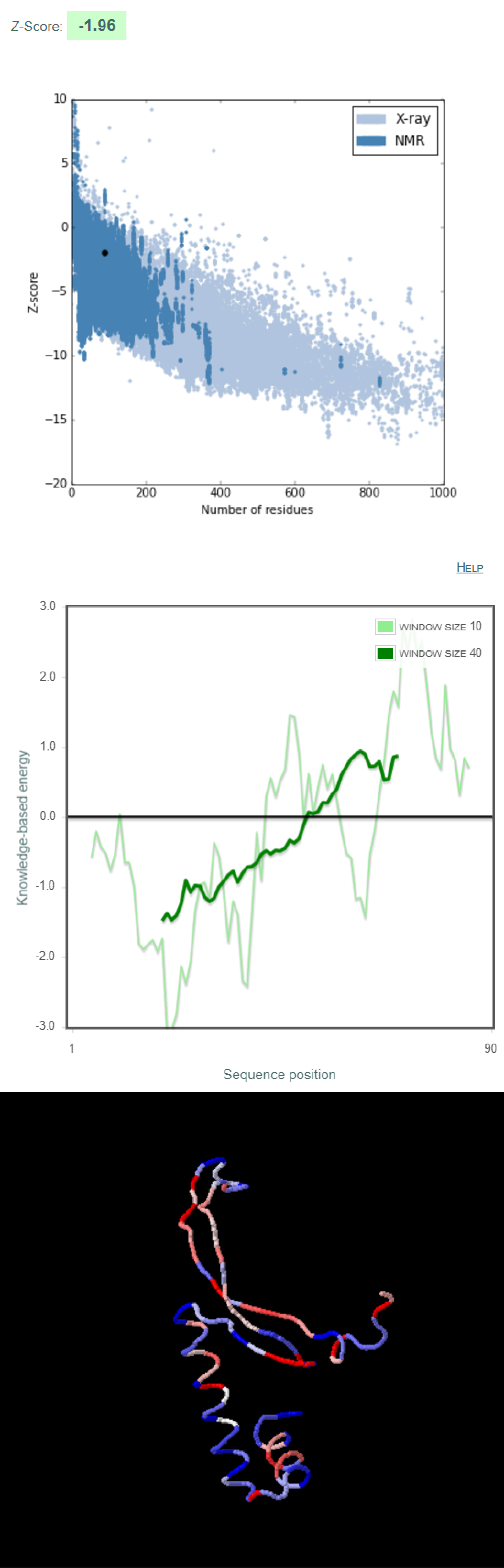

Supplement: S4 File — (ZIP) [file pone.0188037.s004.zip › A_3 v.jpg]

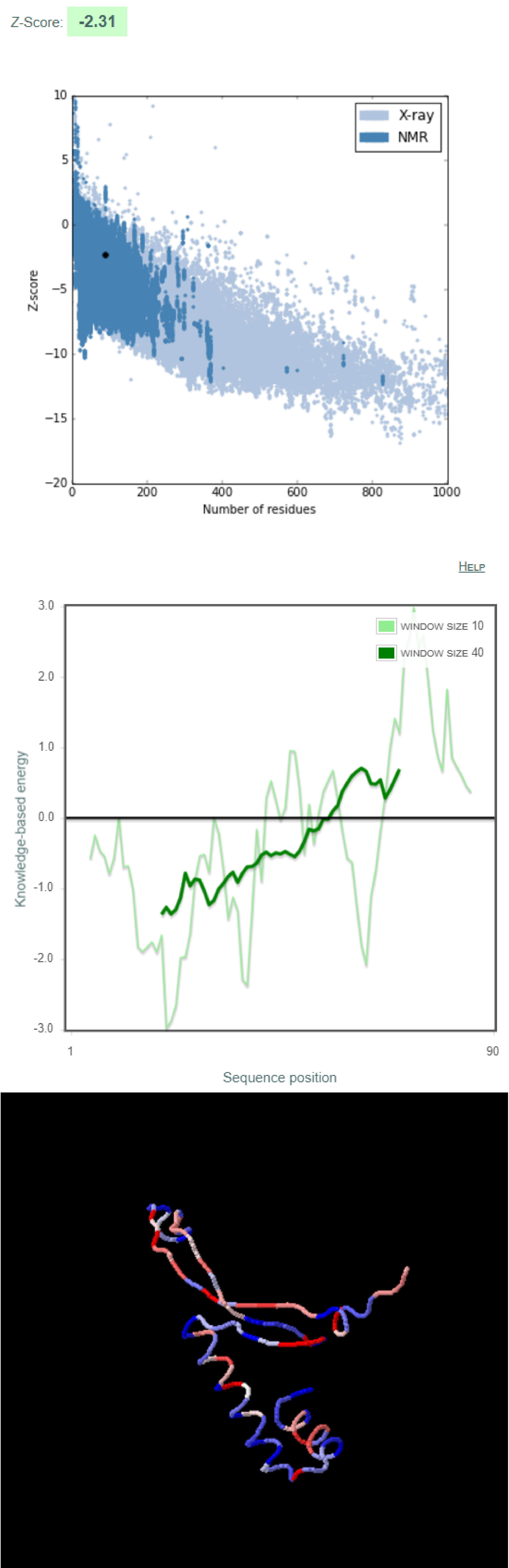

Supplement: S4 File — (ZIP) [file pone.0188037.s004.zip › A_4 v.jpg]

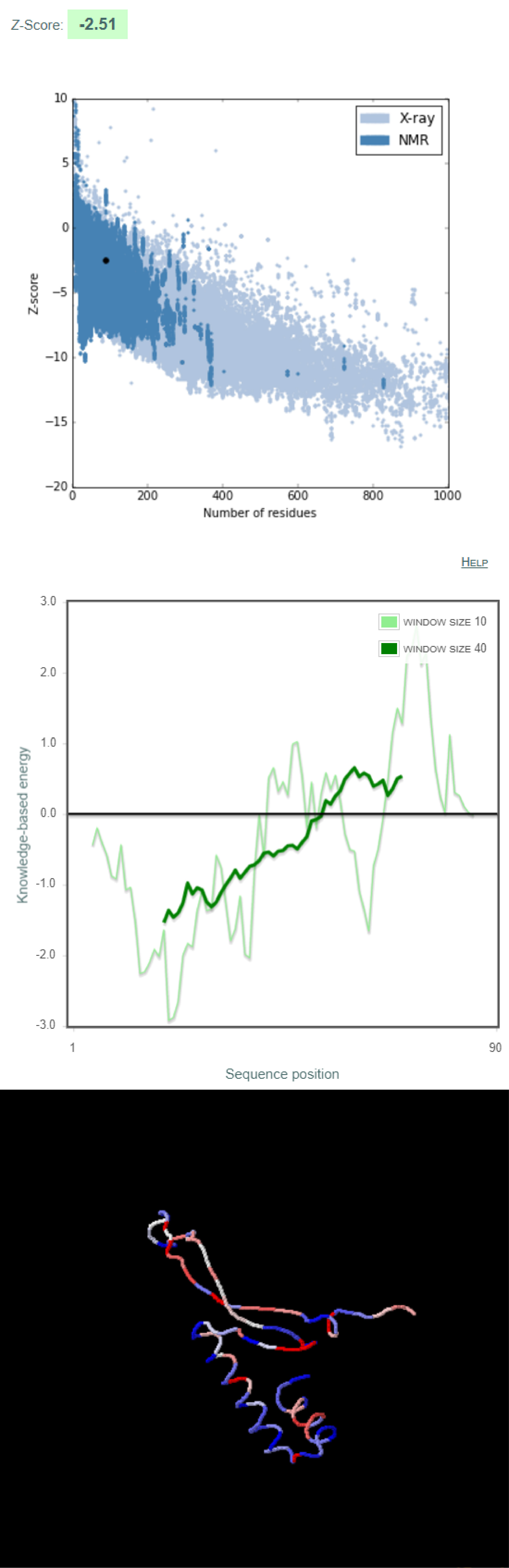

Supplement: S4 File — (ZIP) [file pone.0188037.s004.zip › A_5 v.jpg]

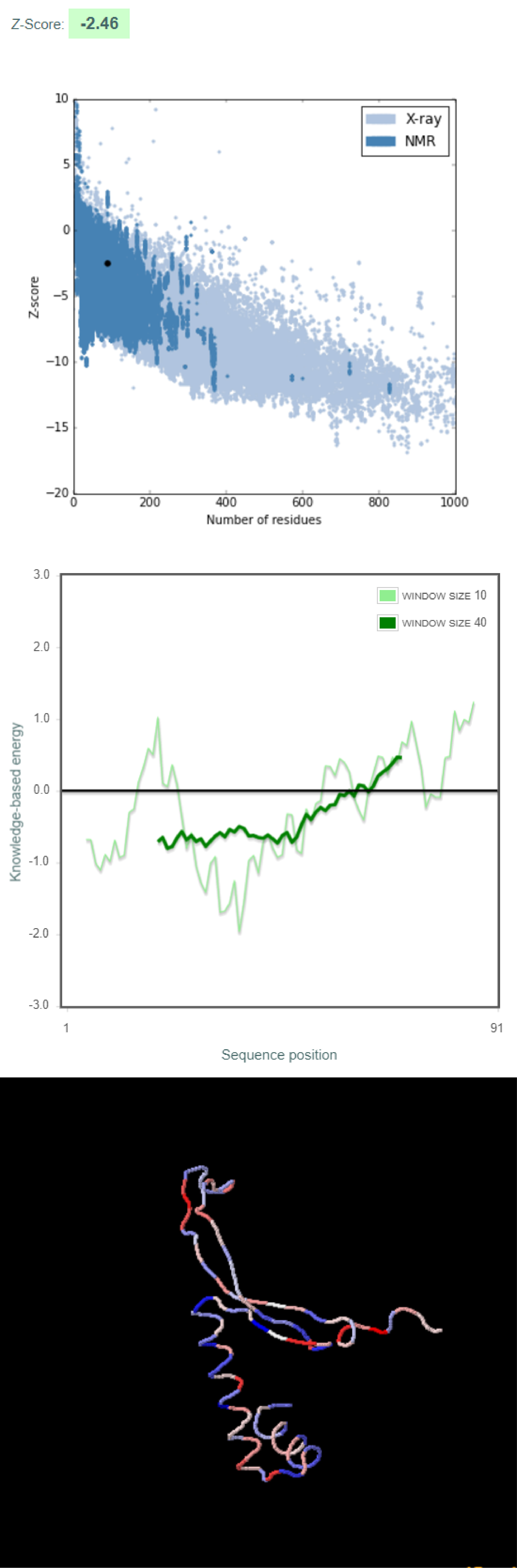

Supplement: S4 File — (ZIP) [file pone.0188037.s004.zip › B2_1 v.jpg]

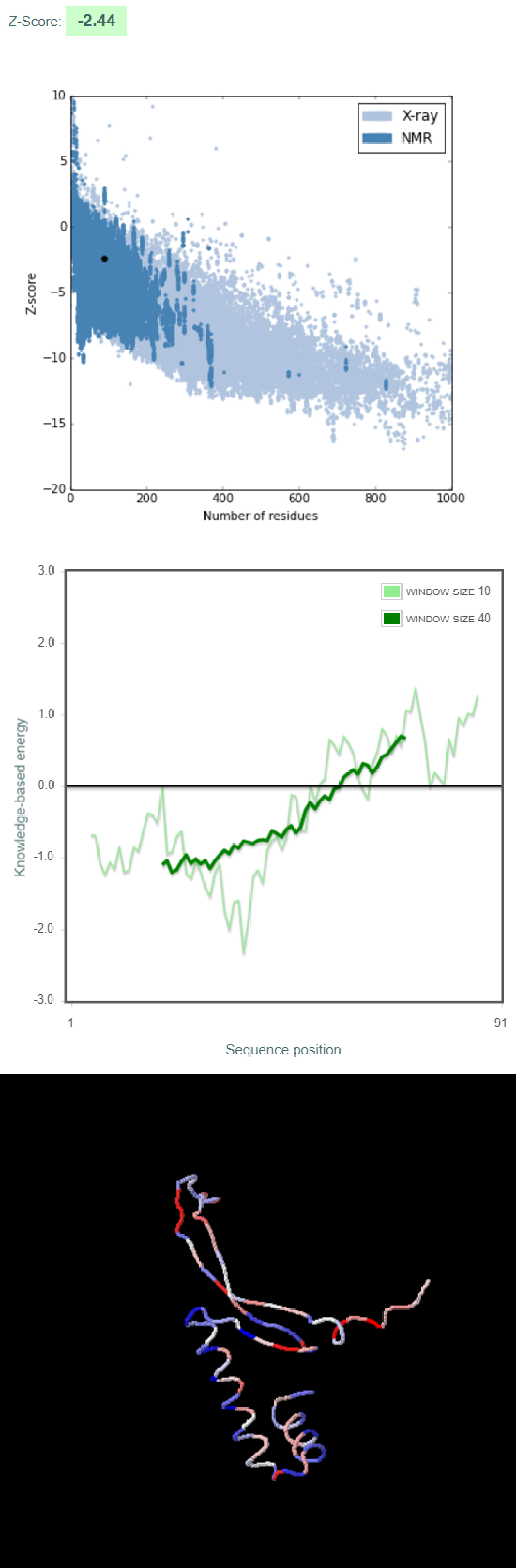

Supplement: S4 File — (ZIP) [file pone.0188037.s004.zip › B2_2 v.jpg]

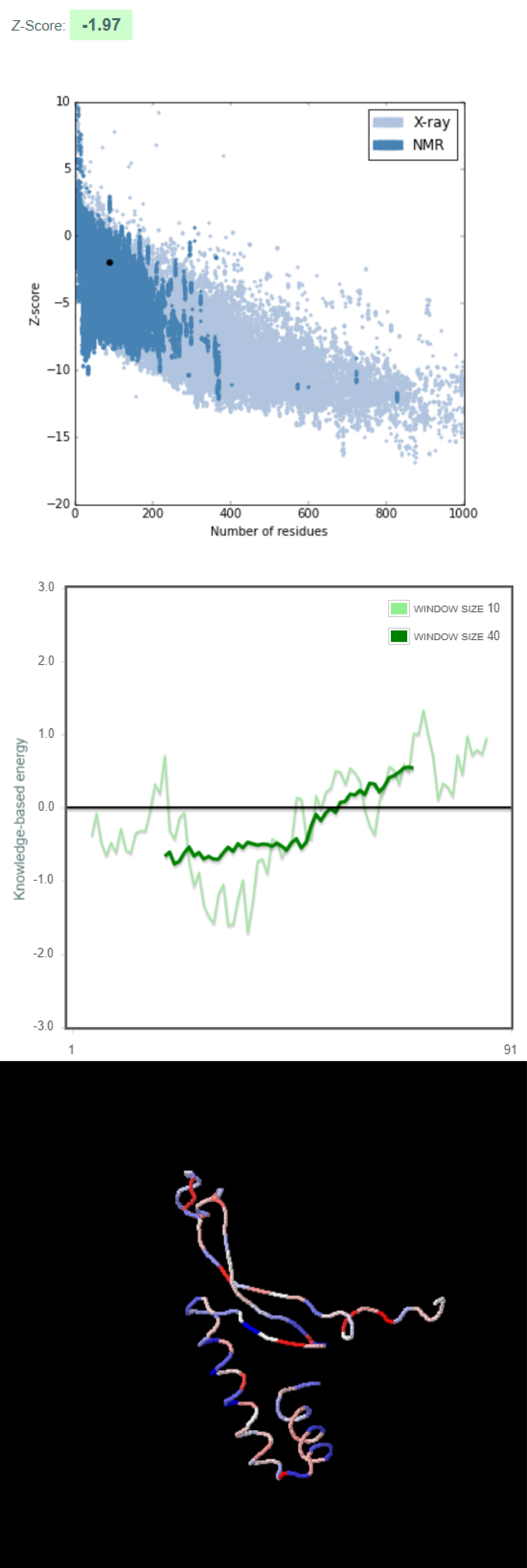

Supplement: S4 File — (ZIP) [file pone.0188037.s004.zip › B2_3 v.jpg]

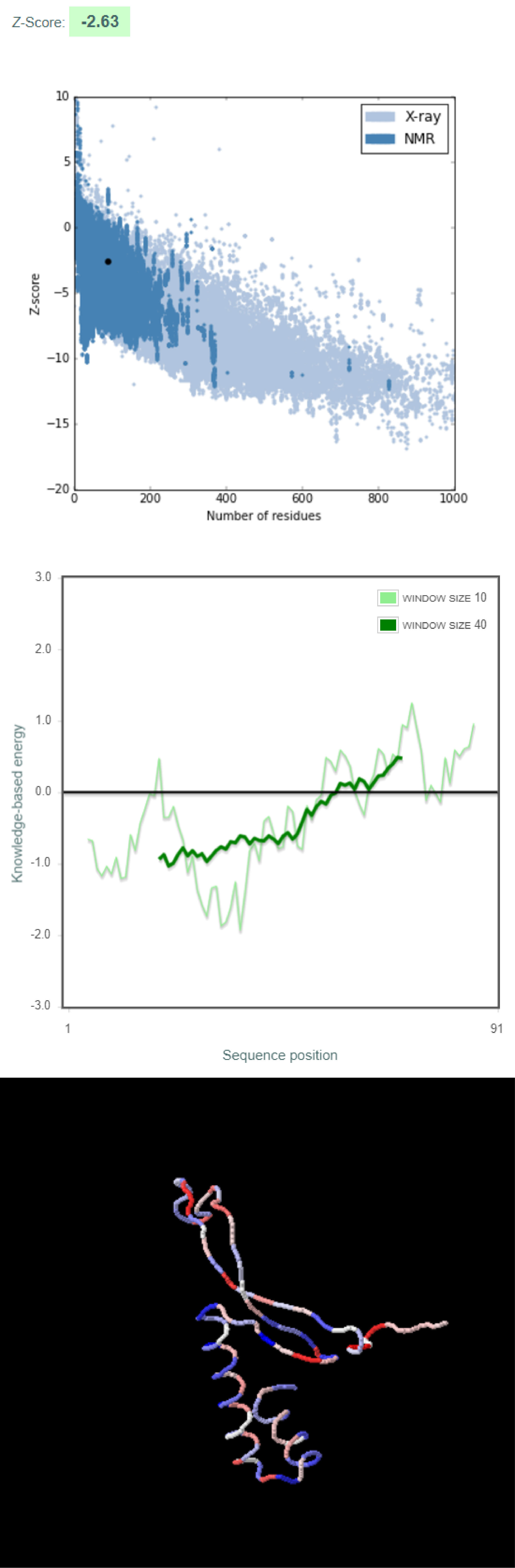

Supplement: S4 File — (ZIP) [file pone.0188037.s004.zip › B2_4 v.jpg]

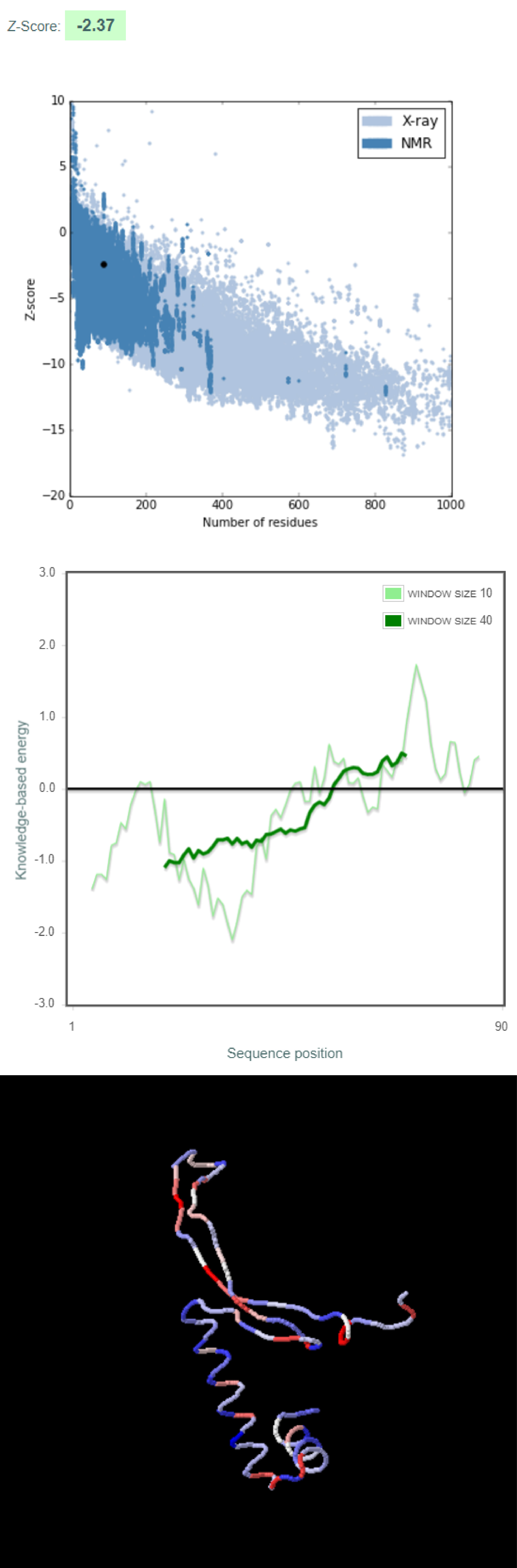

Supplement: S4 File — (ZIP) [file pone.0188037.s004.zip › B3_1 v.jpg]

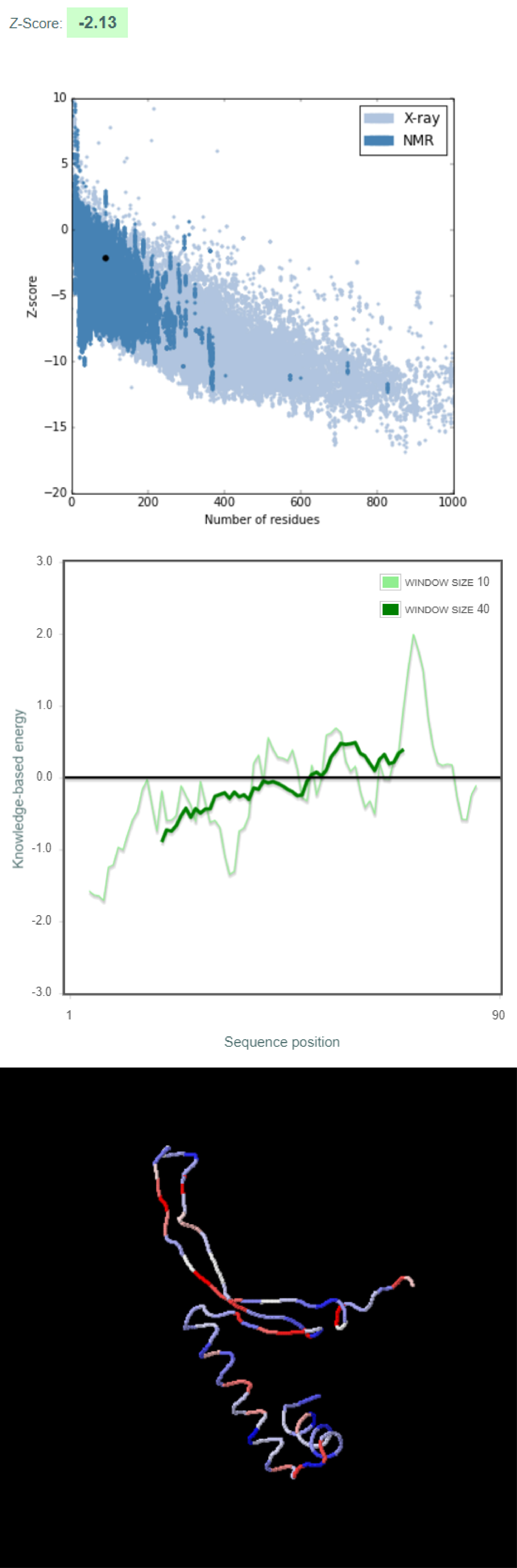

Supplement: S4 File — (ZIP) [file pone.0188037.s004.zip › B3_2 v.jpg]

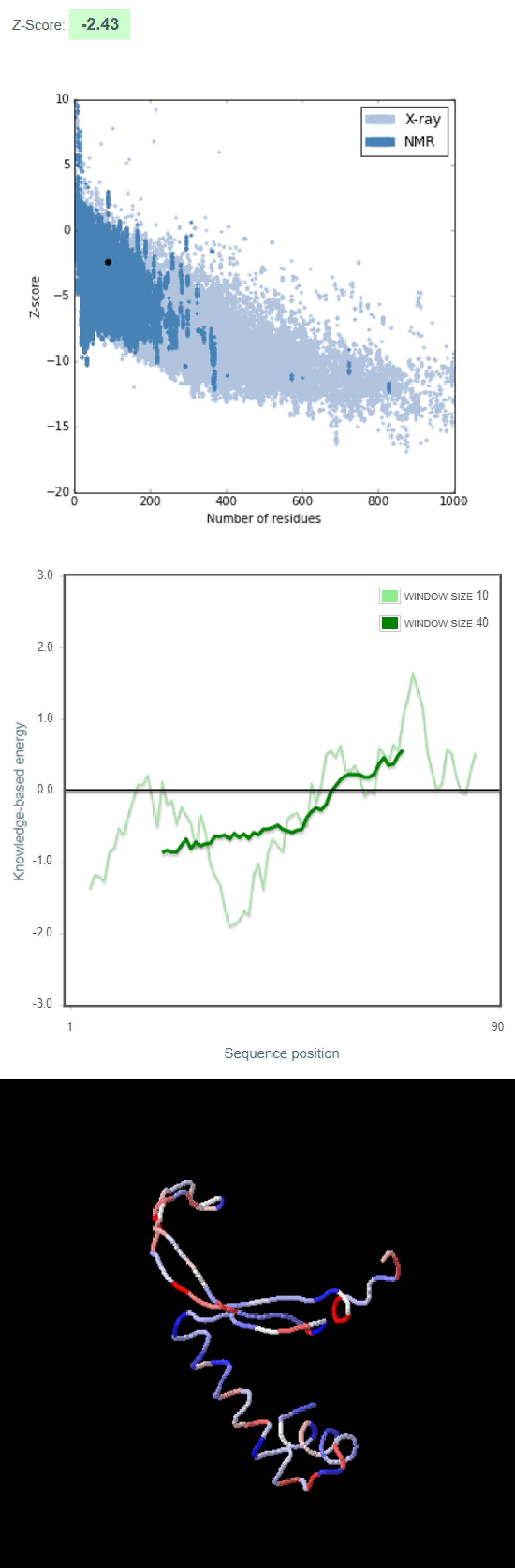

Supplement: S4 File — (ZIP) [file pone.0188037.s004.zip › B3_3 v.jpg]

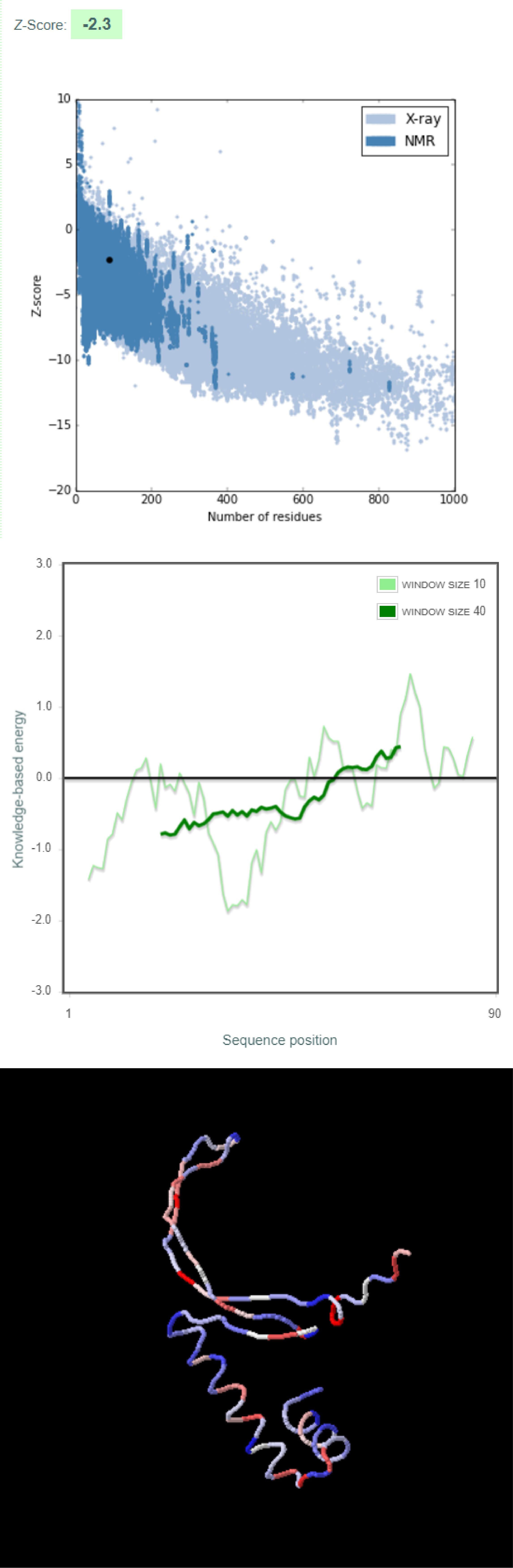

Supplement: S4 File — (ZIP) [file pone.0188037.s004.zip › B3_4 v.jpg]

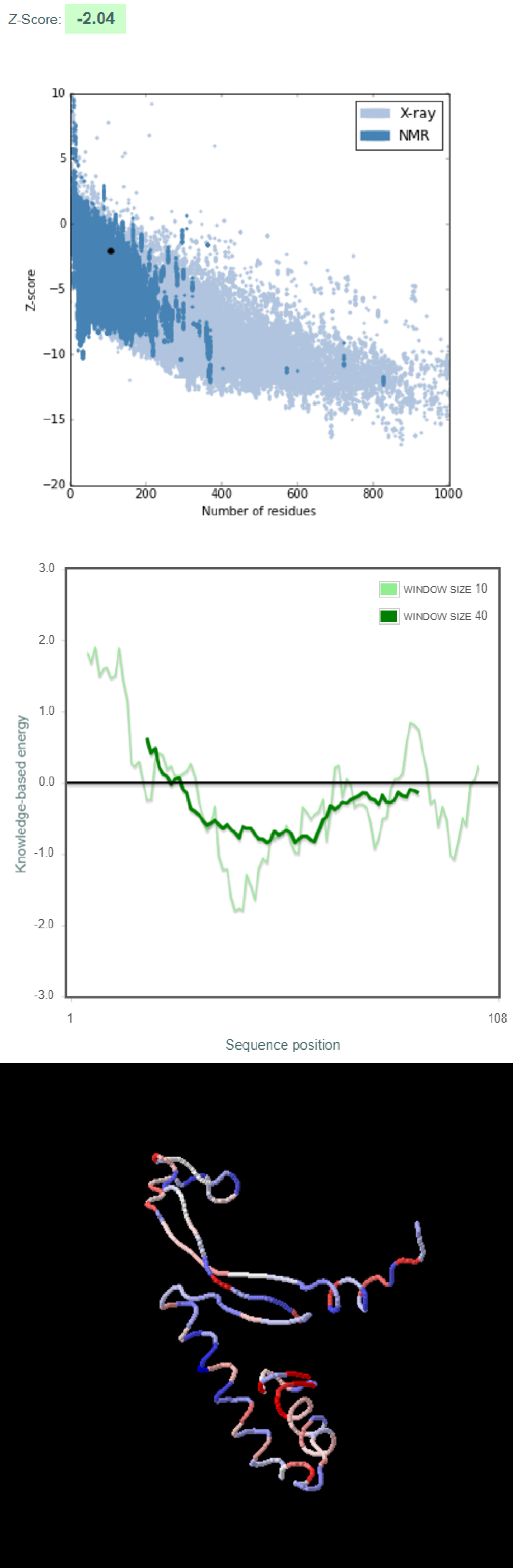

Supplement: S4 File — (ZIP) [file pone.0188037.s004.zip › B4_1 v.jpg]

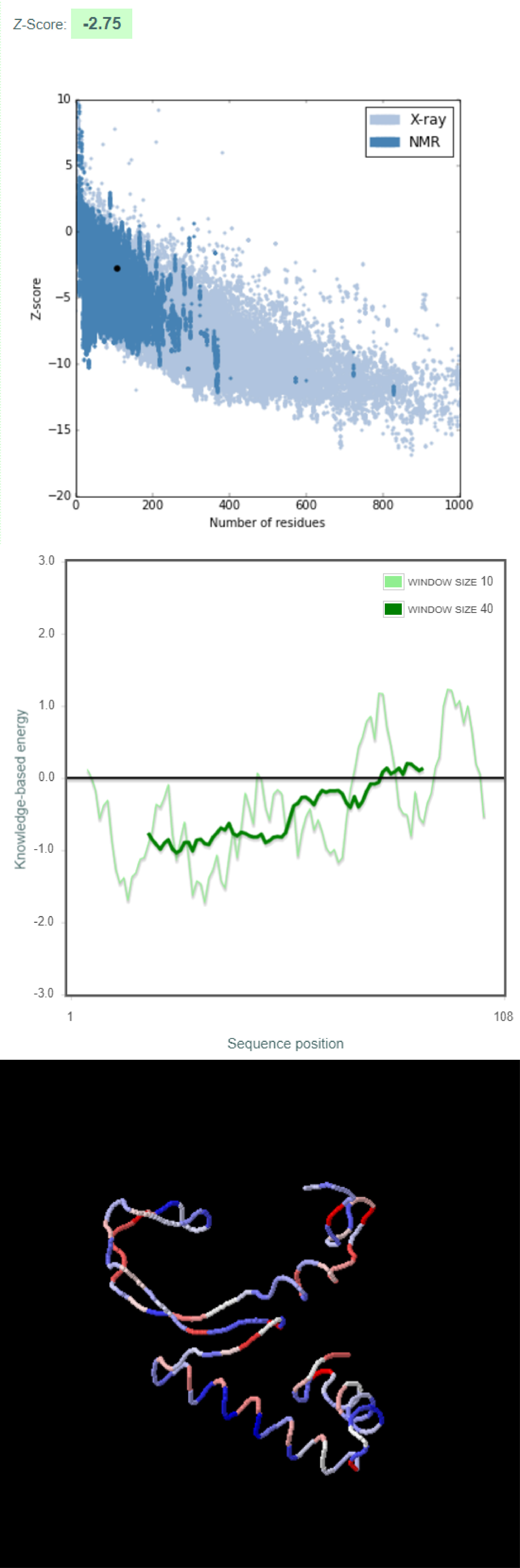

Supplement: S4 File — (ZIP) [file pone.0188037.s004.zip › B4_10 v.jpg]

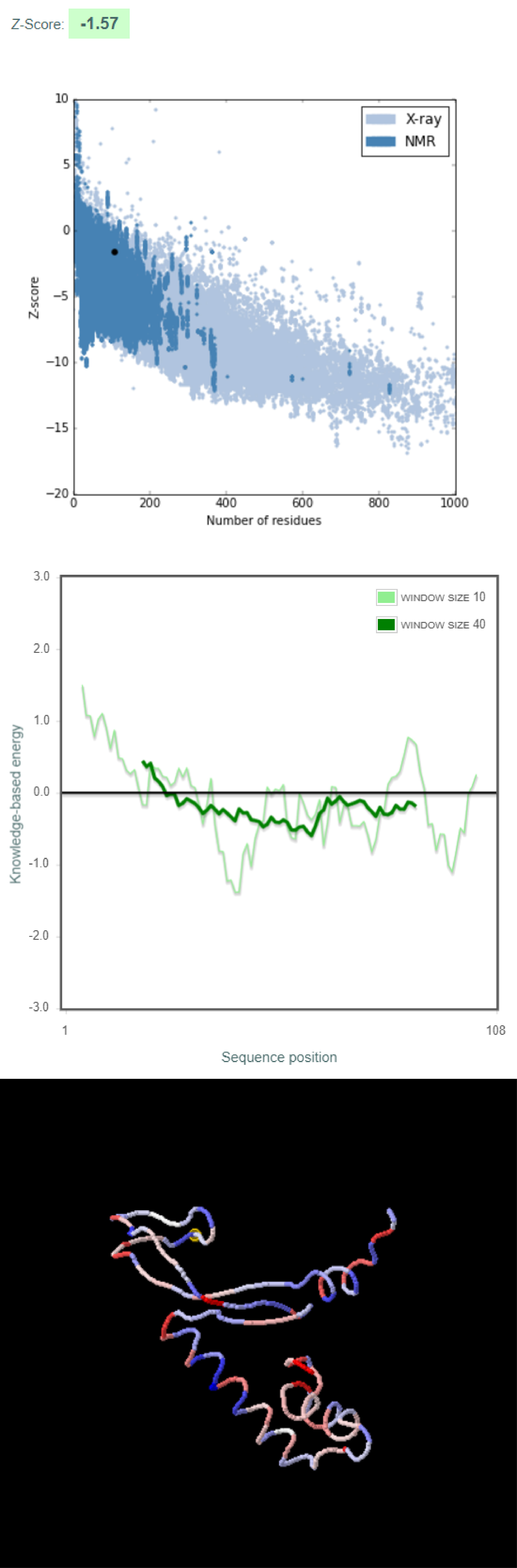

Supplement: S4 File — (ZIP) [file pone.0188037.s004.zip › B4_2 v.jpg]

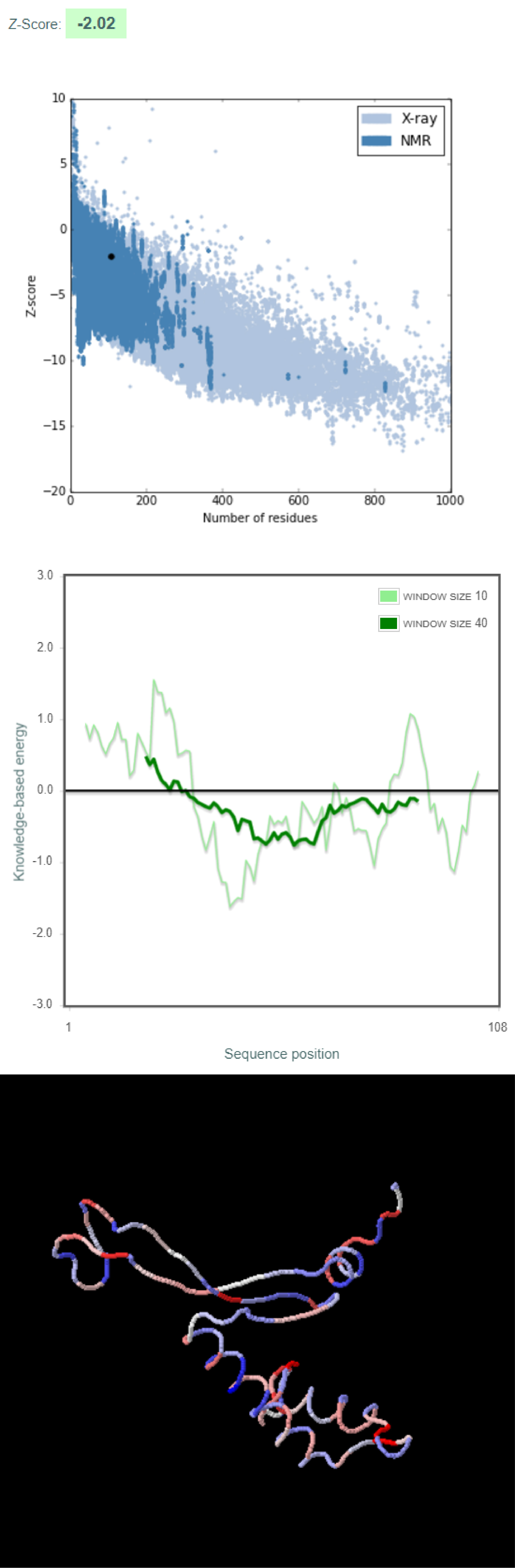

Supplement: S4 File — (ZIP) [file pone.0188037.s004.zip › B4_3 v.jpg]

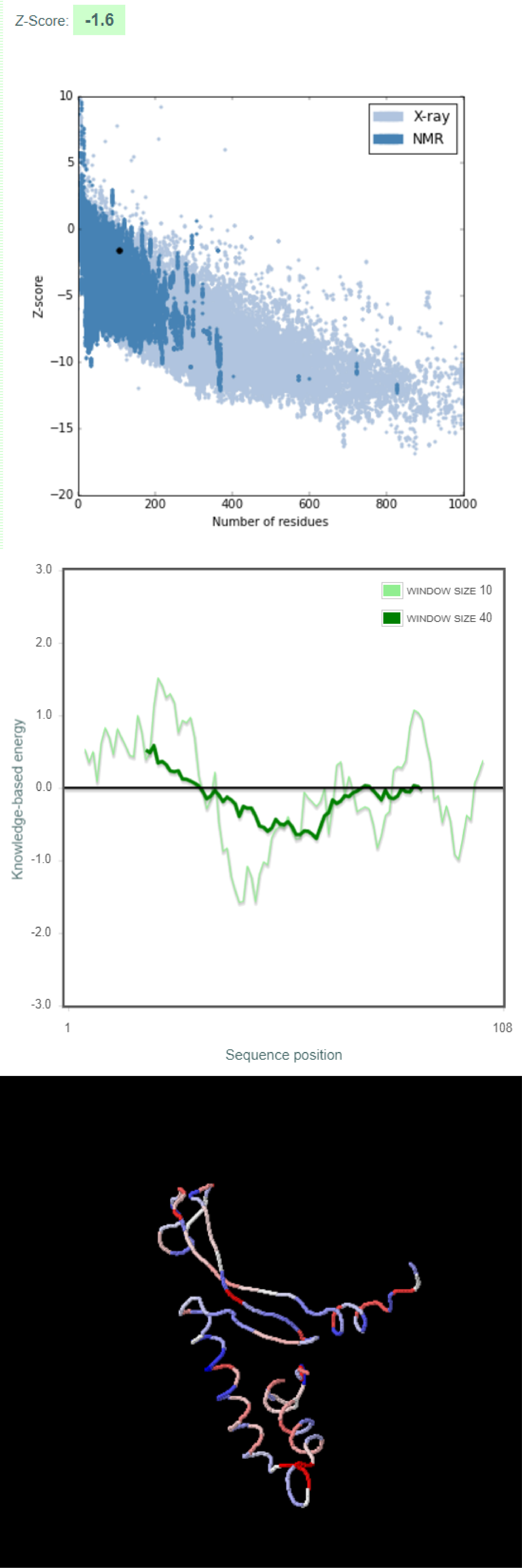

Supplement: S4 File — (ZIP) [file pone.0188037.s004.zip › B4_4 v.jpg]

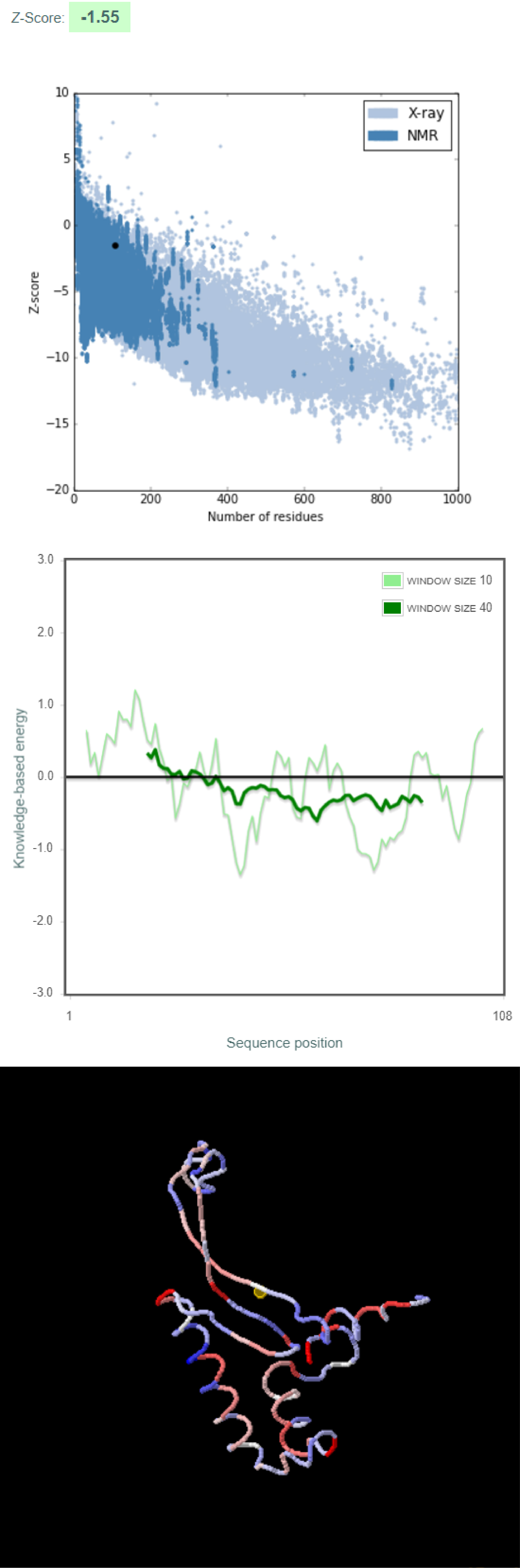

Supplement: S4 File — (ZIP) [file pone.0188037.s004.zip › B4_5 v.jpg]

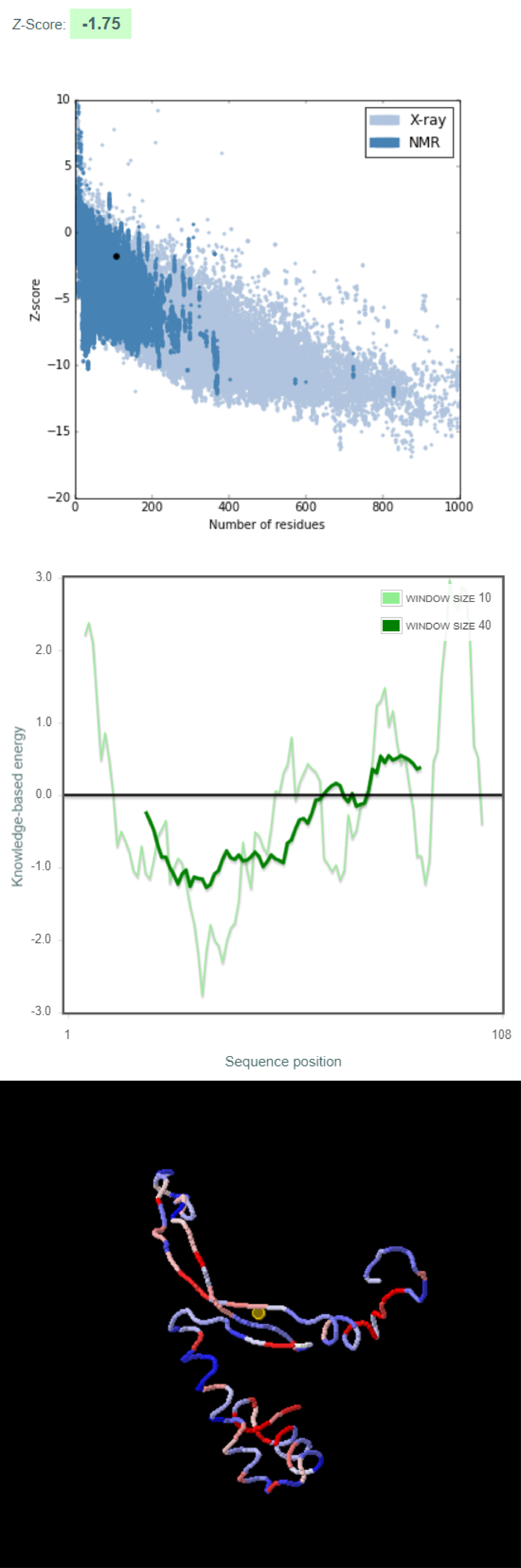

Supplement: S4 File — (ZIP) [file pone.0188037.s004.zip › B4_6 v.jpg]

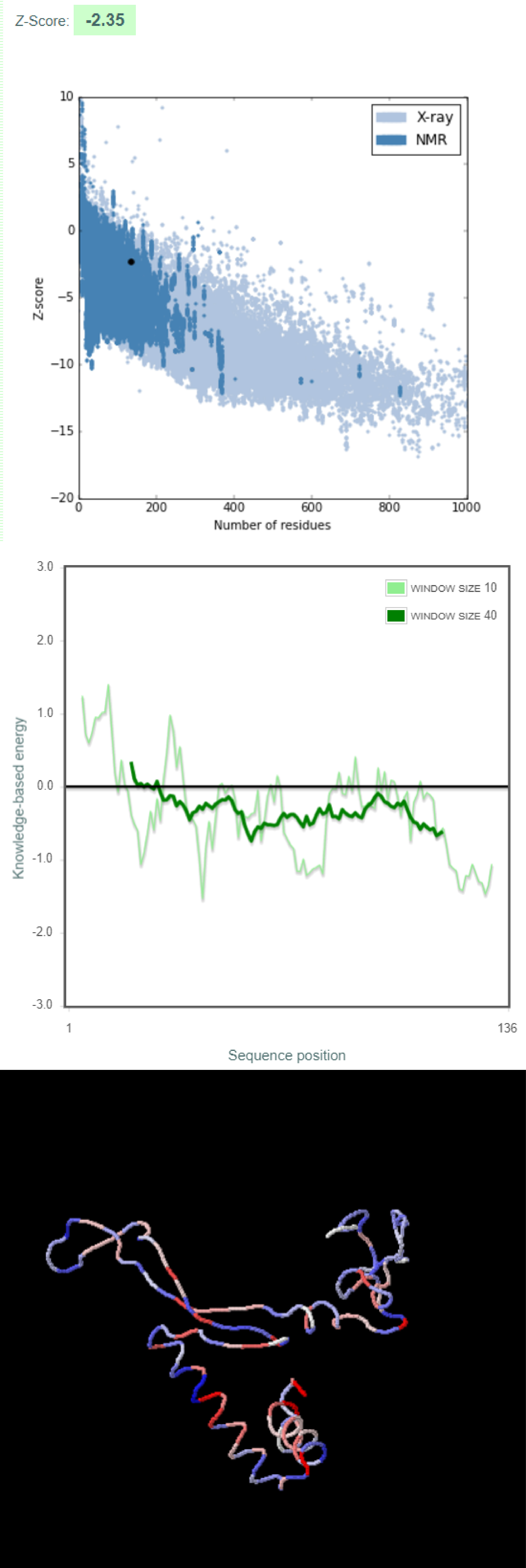

Supplement: S4 File — (ZIP) [file pone.0188037.s004.zip › B4_7 v.jpg]

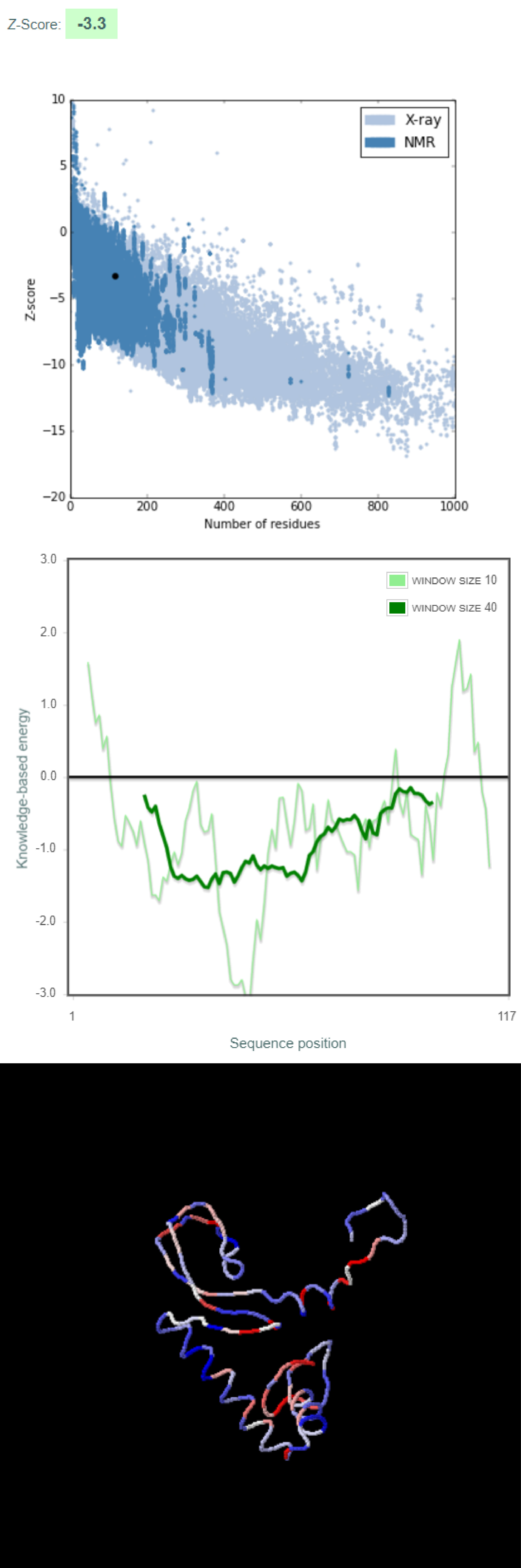

Supplement: S4 File — (ZIP) [file pone.0188037.s004.zip › B4_8 v.jpg]

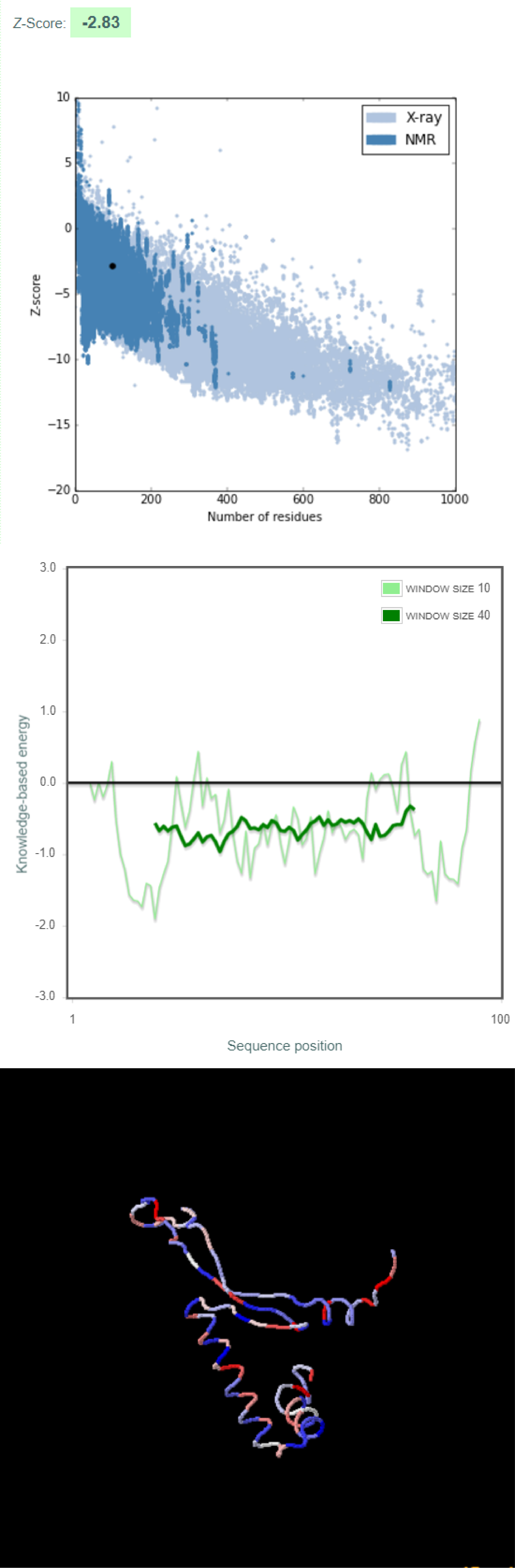

Supplement: S4 File — (ZIP) [file pone.0188037.s004.zip › B4_9 v.jpg]

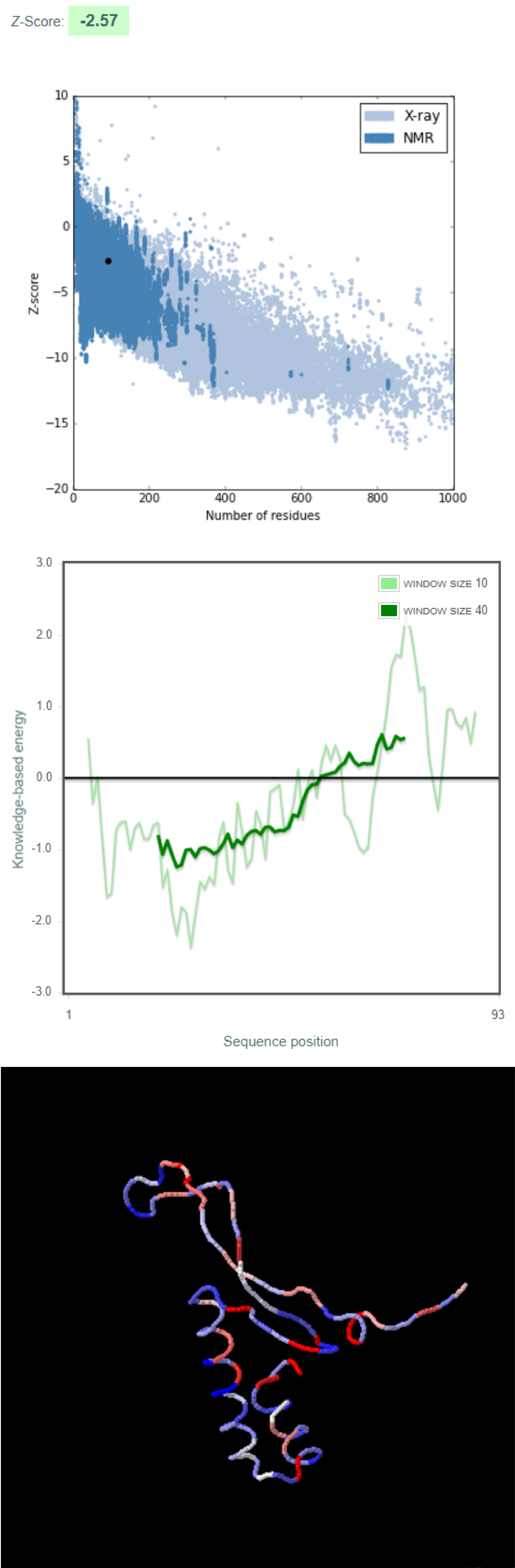

Supplement: S4 File — (ZIP) [file pone.0188037.s004.zip › B_1 v.jpg]

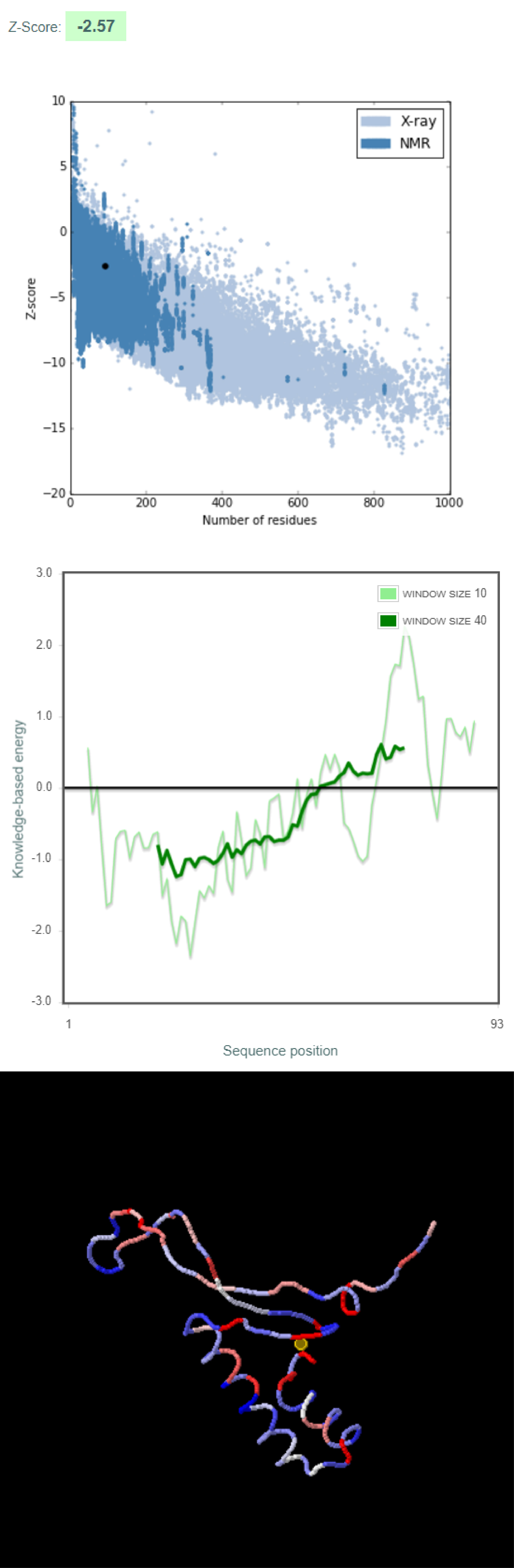

Supplement: S4 File — (ZIP) [file pone.0188037.s004.zip › B_2 v.jpg]

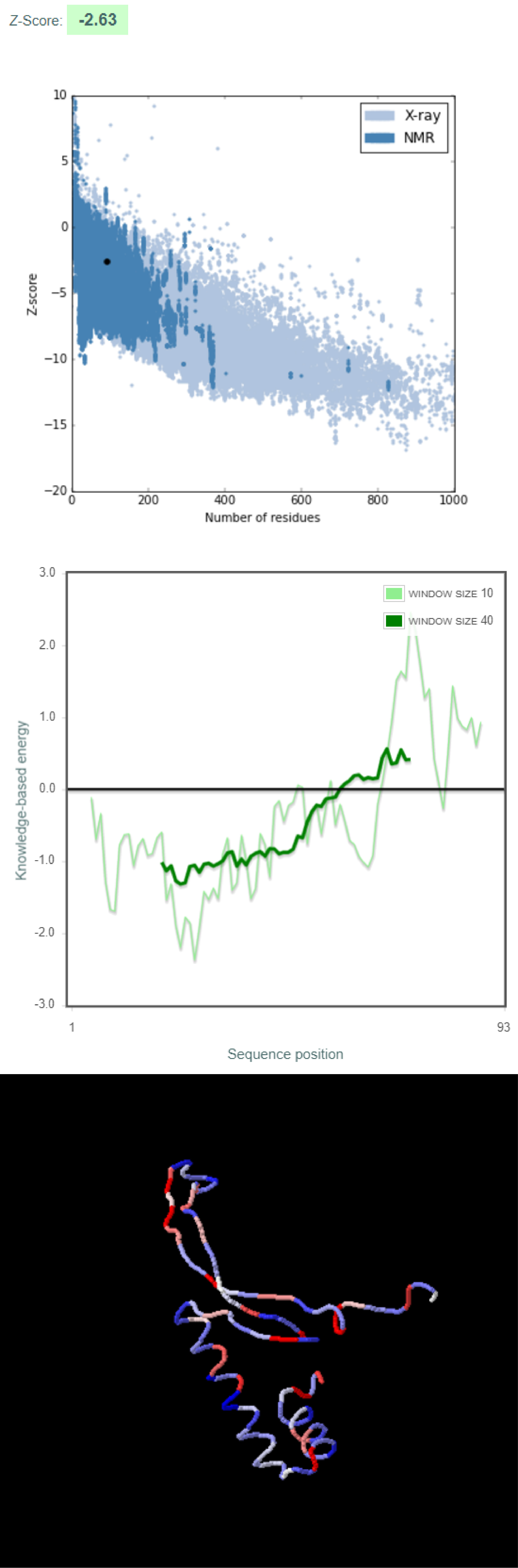

Supplement: S4 File — (ZIP) [file pone.0188037.s004.zip › B_3 v.jpg]

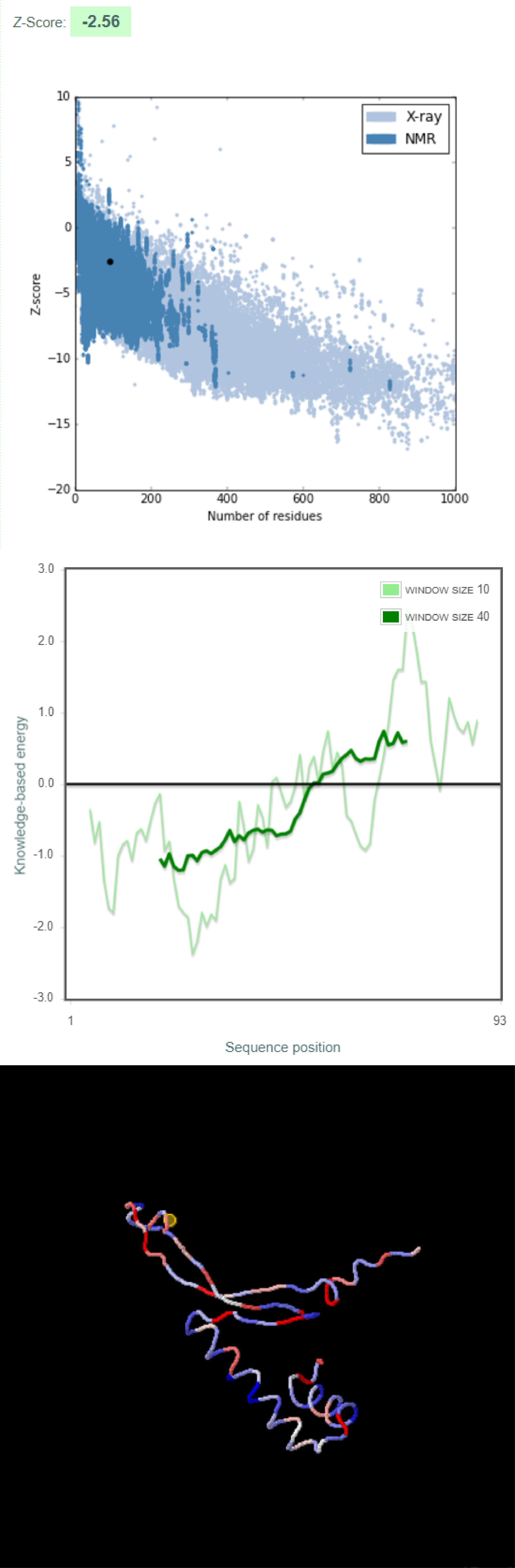

Supplement: S4 File — (ZIP) [file pone.0188037.s004.zip › B_4 v.jpg]

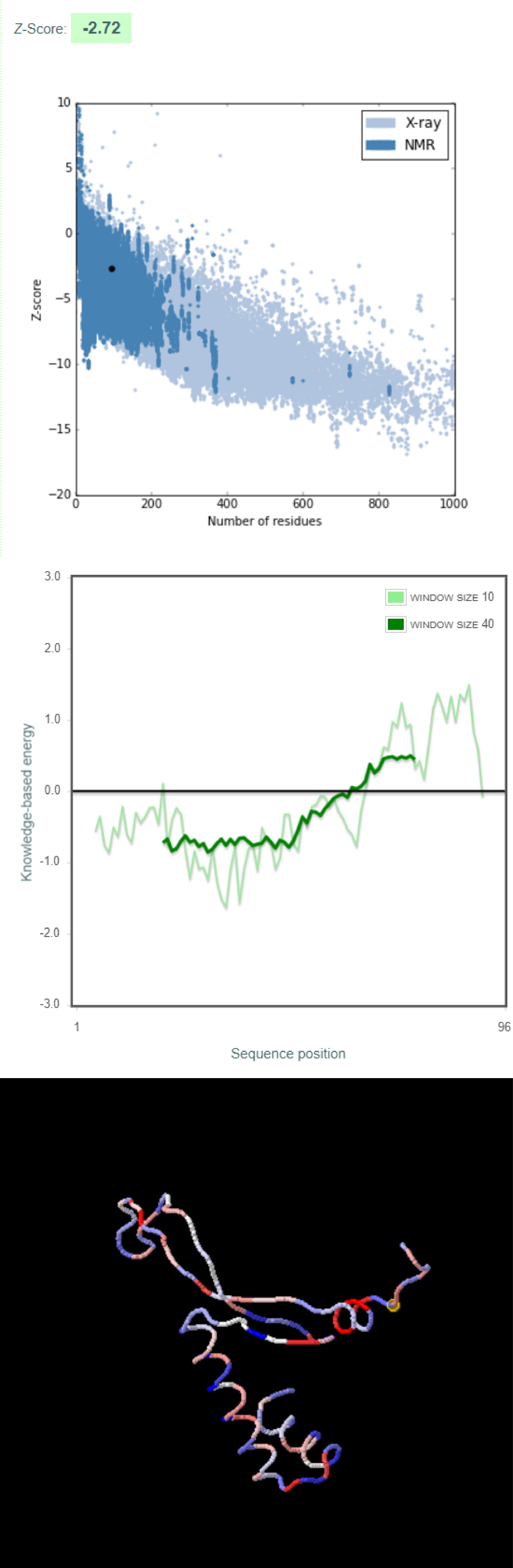

Supplement: S4 File — (ZIP) [file pone.0188037.s004.zip › C2_1 v.jpg]

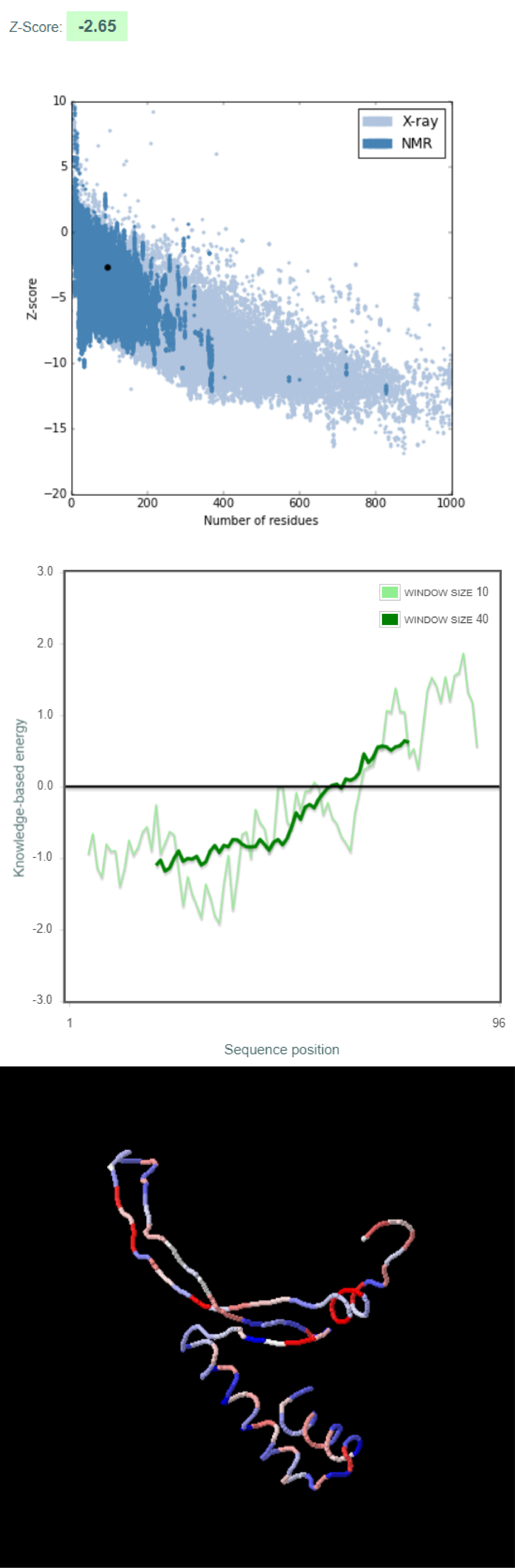

Supplement: S4 File — (ZIP) [file pone.0188037.s004.zip › C2_2 v.jpg]

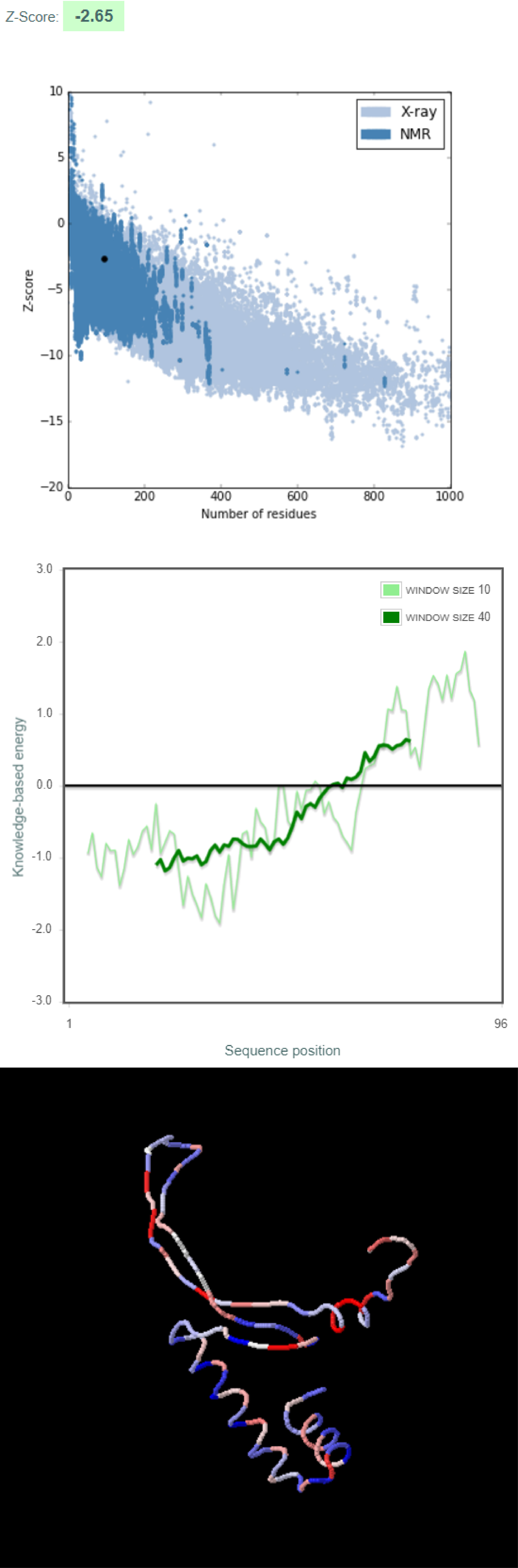

Supplement: S4 File — (ZIP) [file pone.0188037.s004.zip › C2_3 v.jpg]

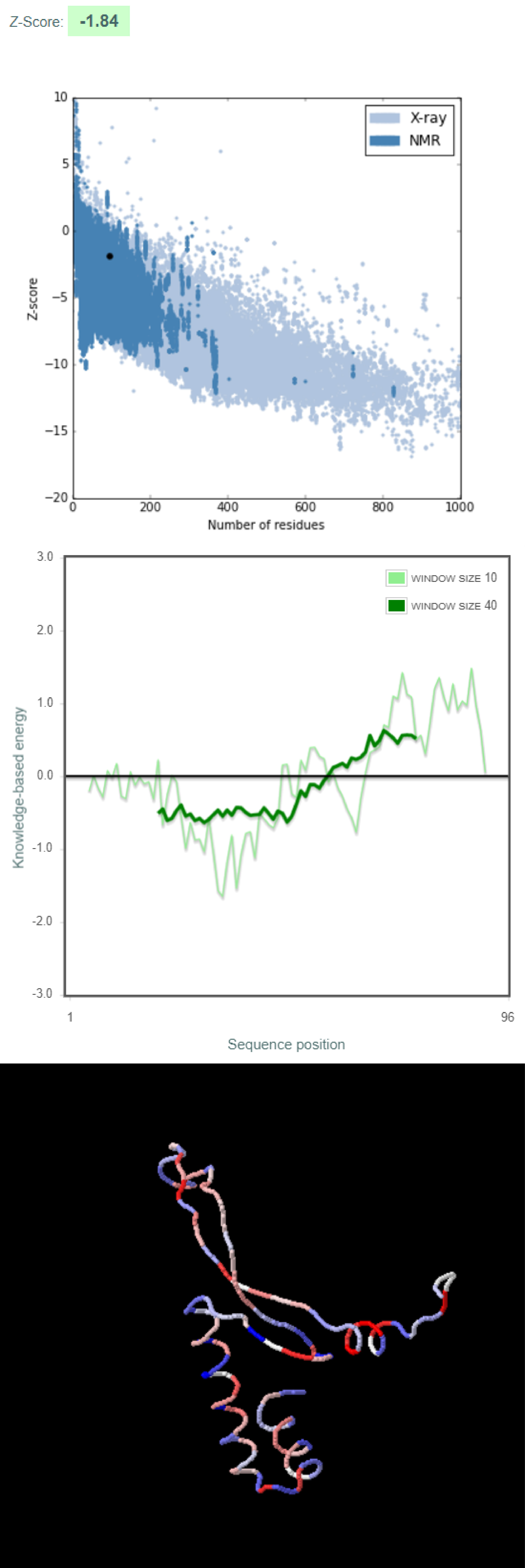

Supplement: S4 File — (ZIP) [file pone.0188037.s004.zip › C2_4 v.jpg]

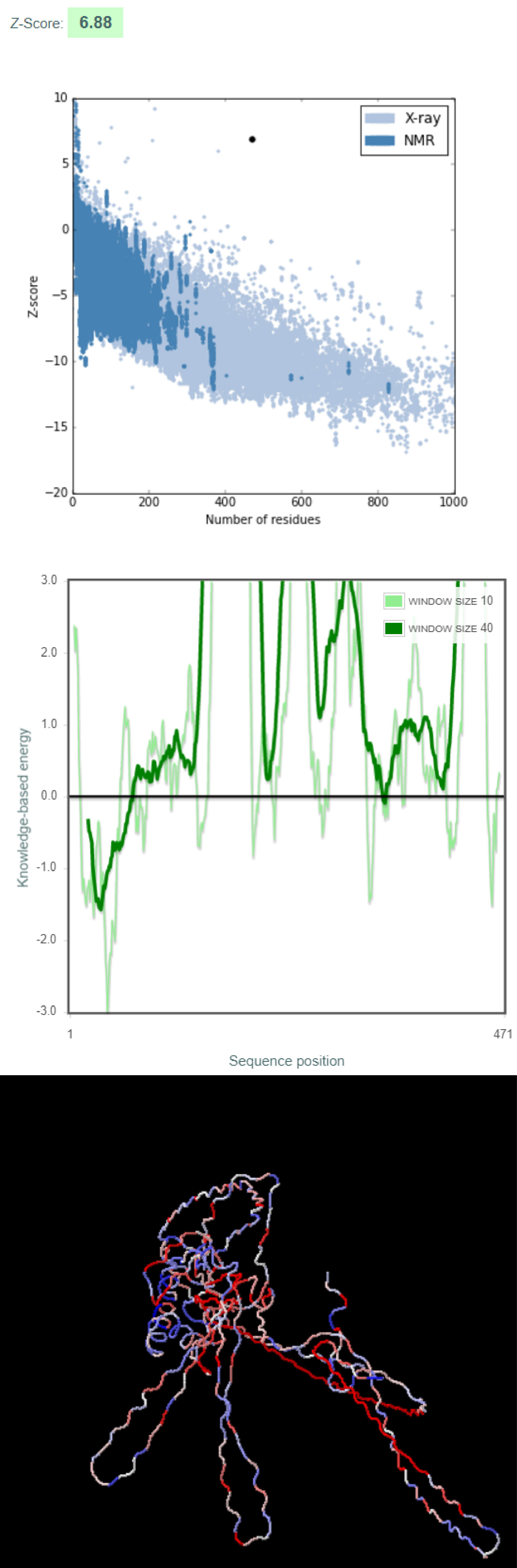

Supplement: S4 File — (ZIP) [file pone.0188037.s004.zip › C3_1 v.jpg]

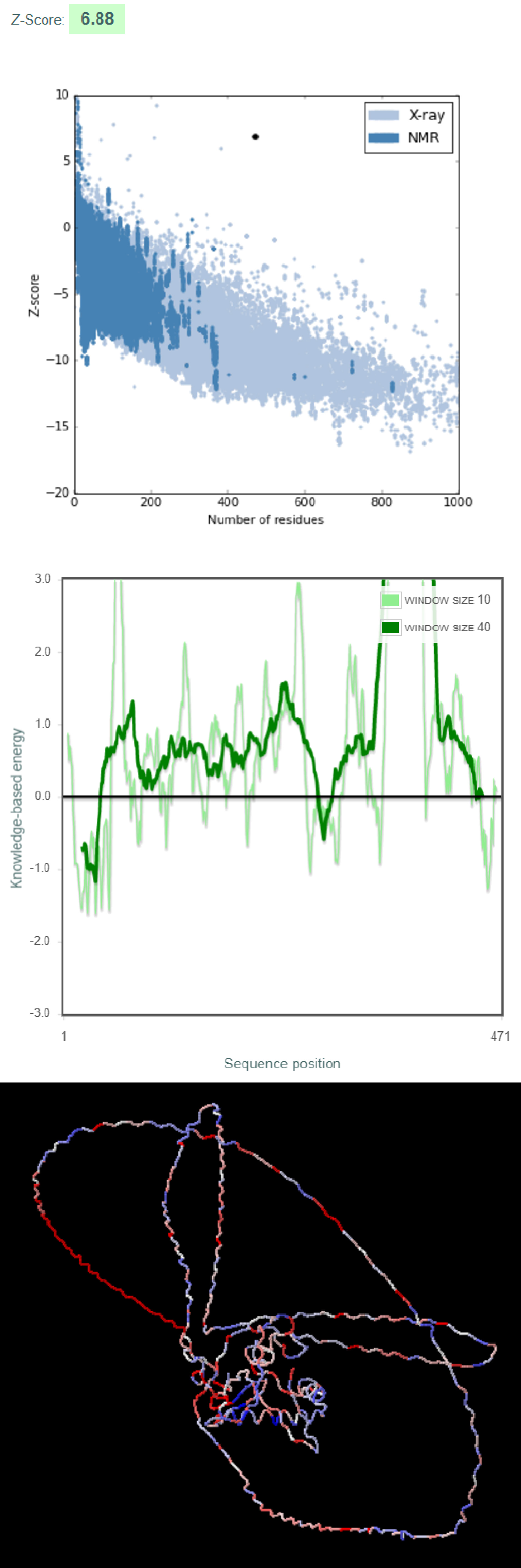

Supplement: S4 File — (ZIP) [file pone.0188037.s004.zip › C3_2 v.jpg]

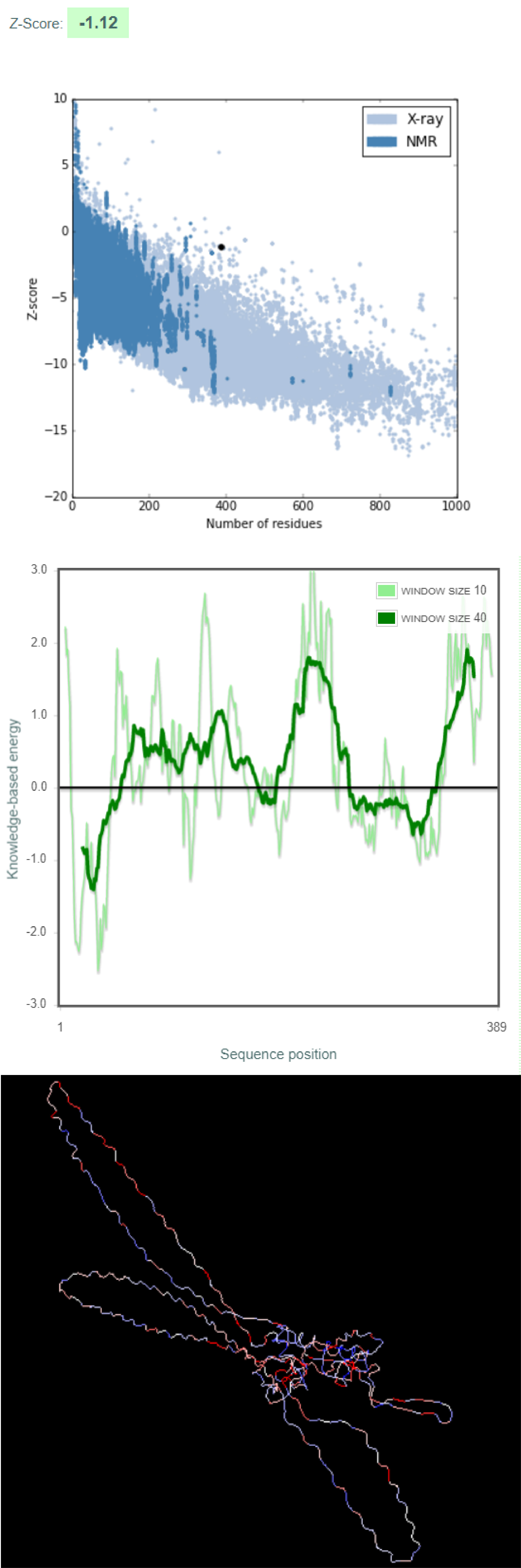

Supplement: S4 File — (ZIP) [file pone.0188037.s004.zip › C3_3 v.jpg]

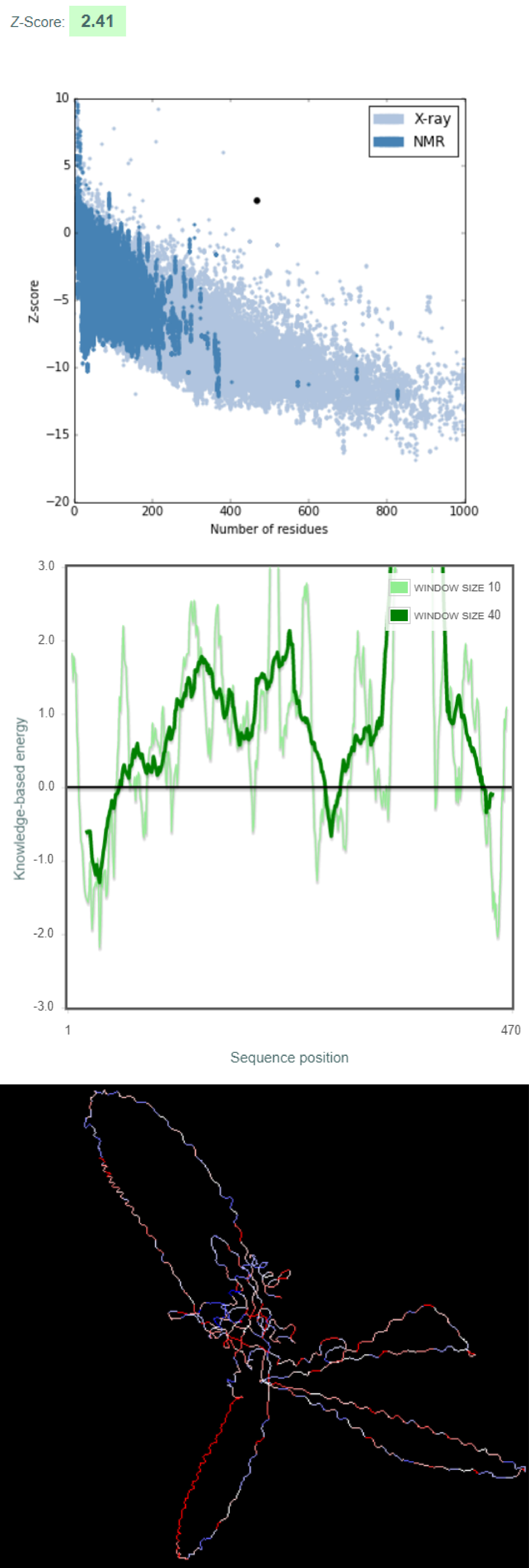

Supplement: S4 File — (ZIP) [file pone.0188037.s004.zip › C3_4 v.jpg]

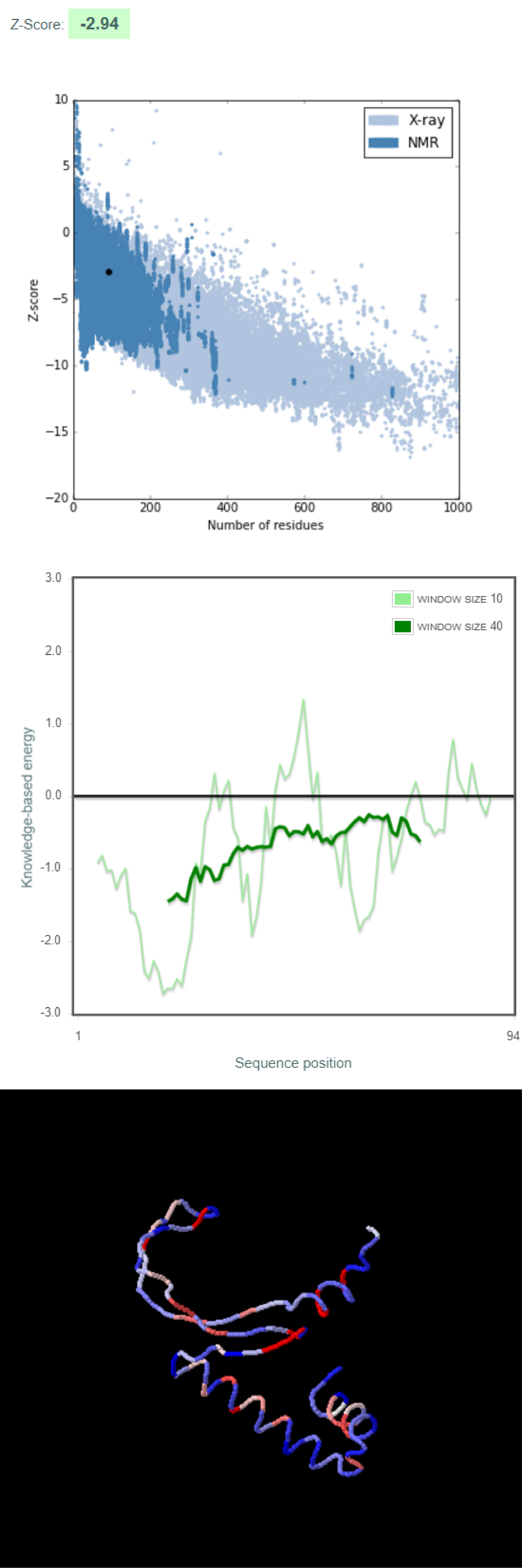

Supplement: S4 File — (ZIP) [file pone.0188037.s004.zip › C4_1 v.jpg]

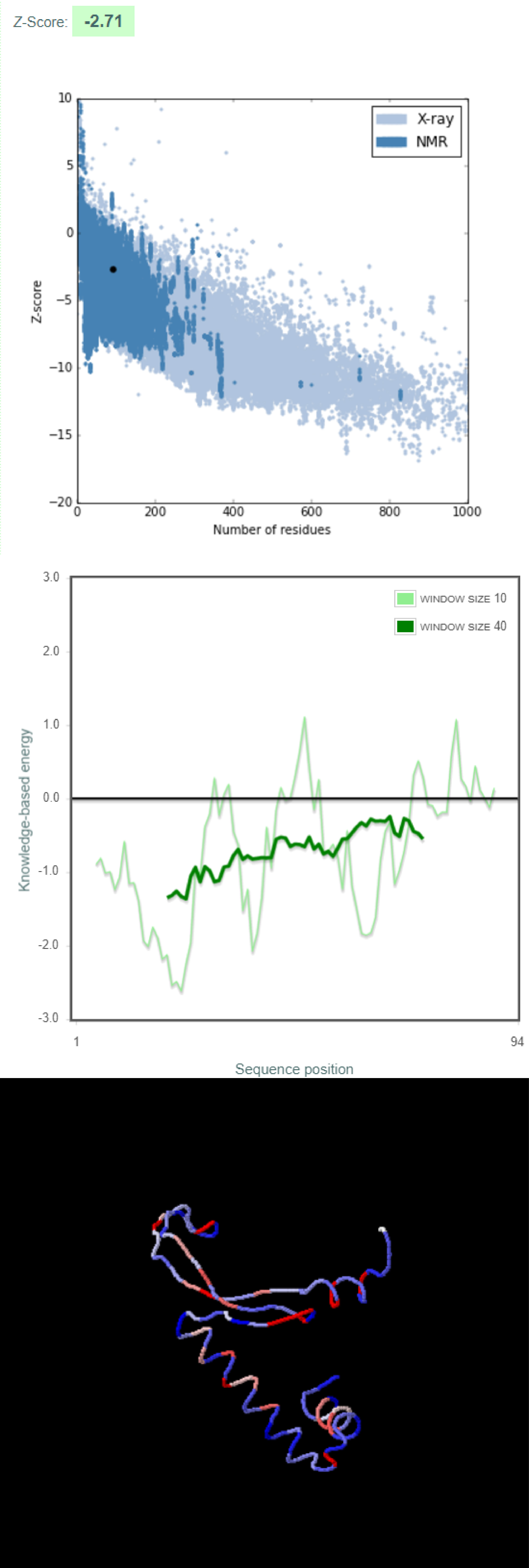

Supplement: S4 File — (ZIP) [file pone.0188037.s004.zip › C4_2 v.jpg]

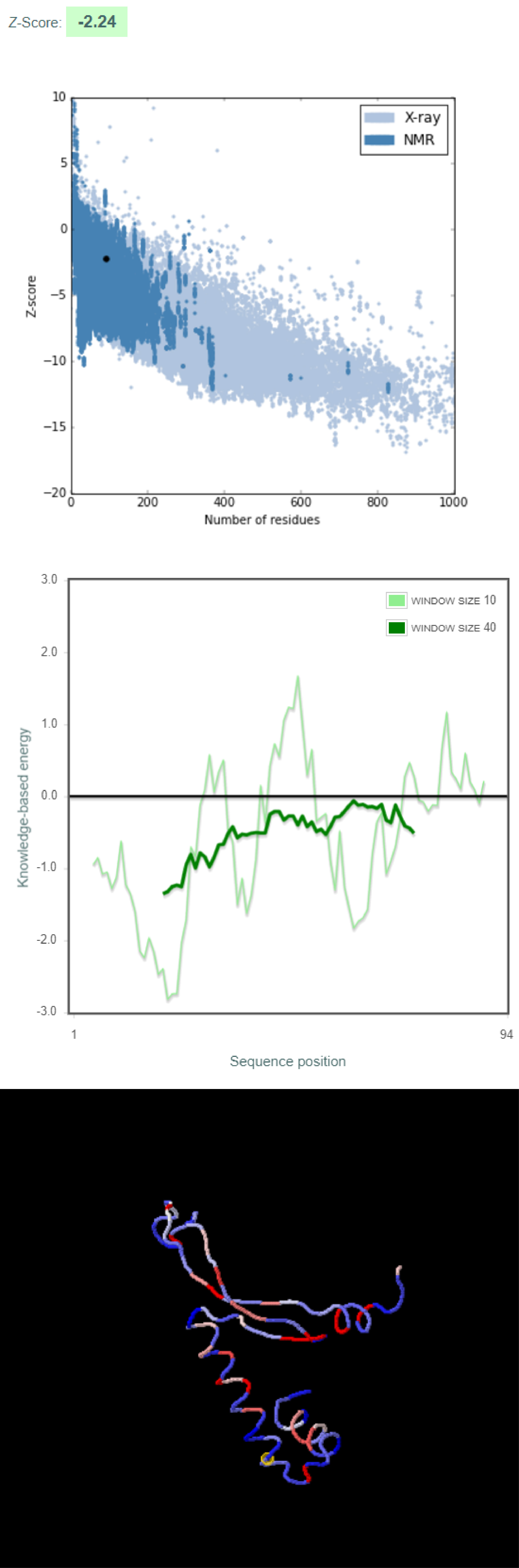

Supplement: S4 File — (ZIP) [file pone.0188037.s004.zip › C4_3 v.jpg]

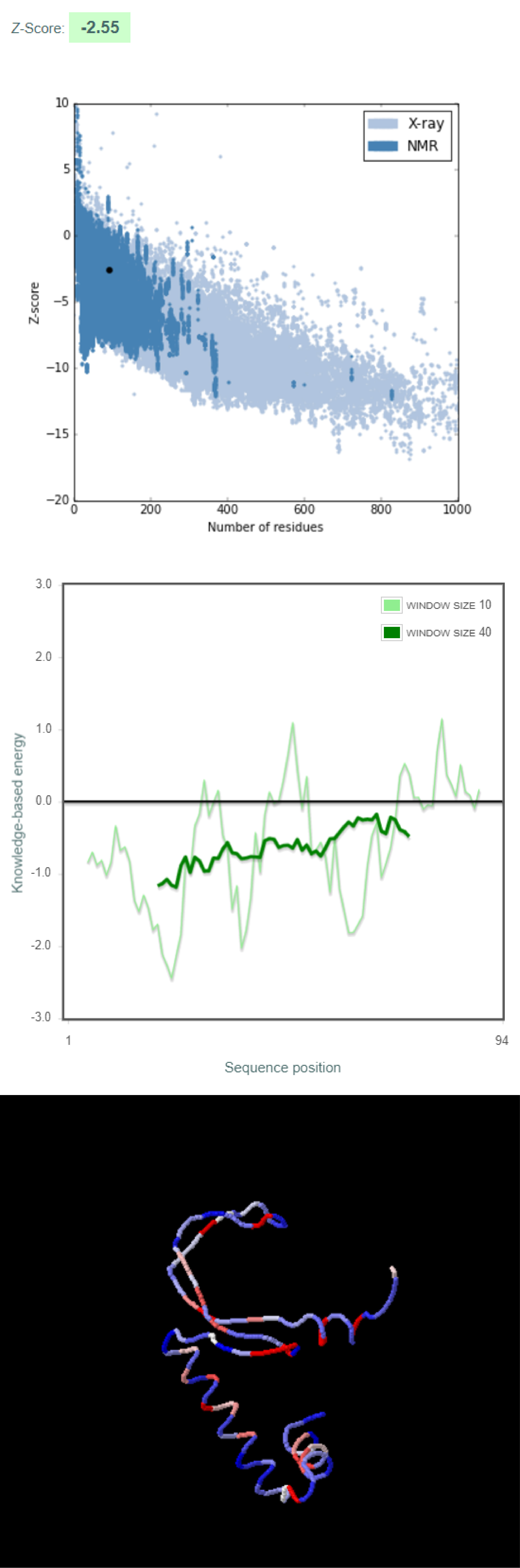

Supplement: S4 File — (ZIP) [file pone.0188037.s004.zip › C4_4 v.jpg]

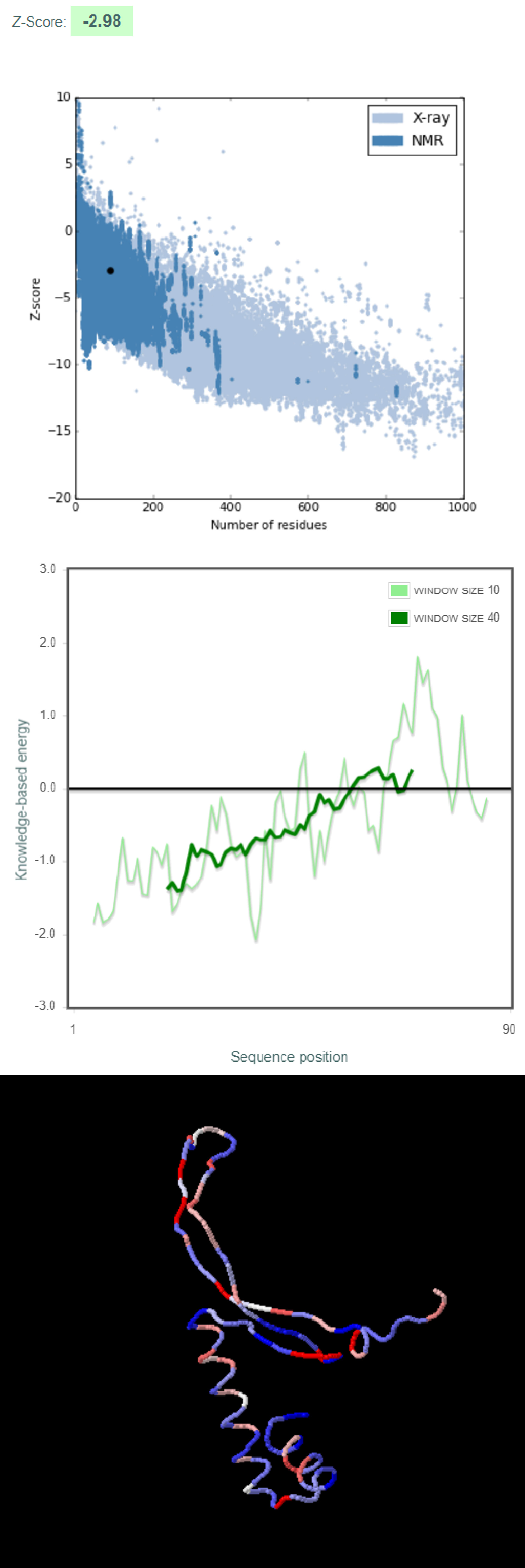

Supplement: S4 File — (ZIP) [file pone.0188037.s004.zip › C_1 v.jpg]

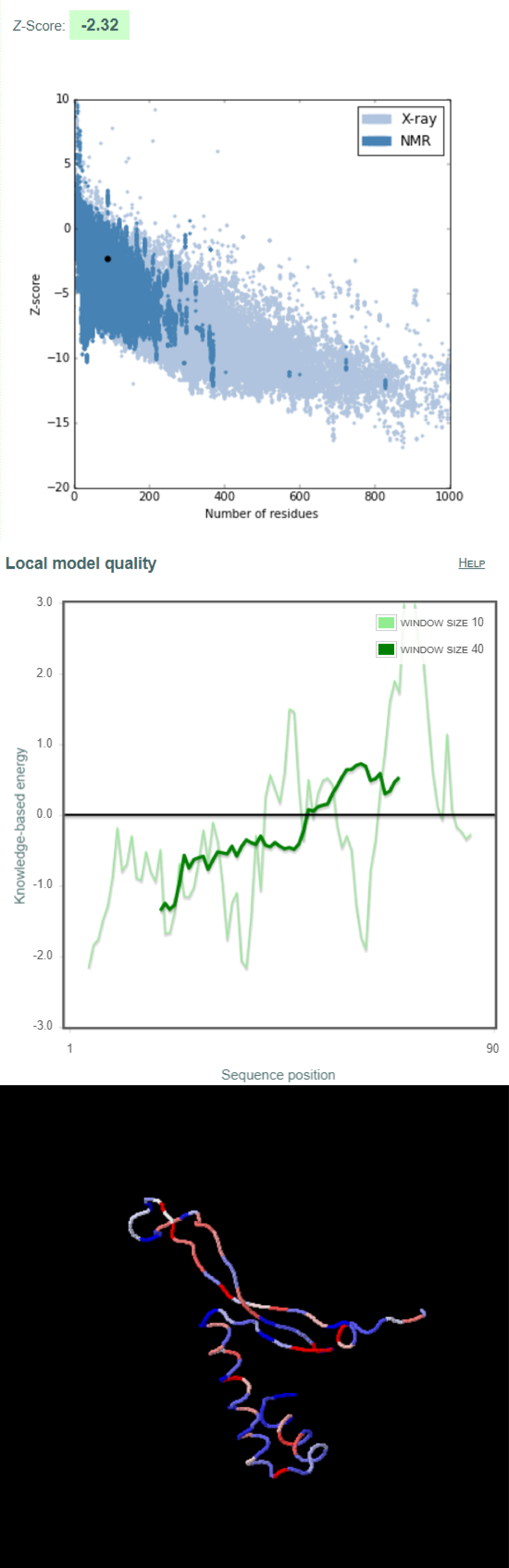

Supplement: S4 File — (ZIP) [file pone.0188037.s004.zip › C_2 v.jpg]

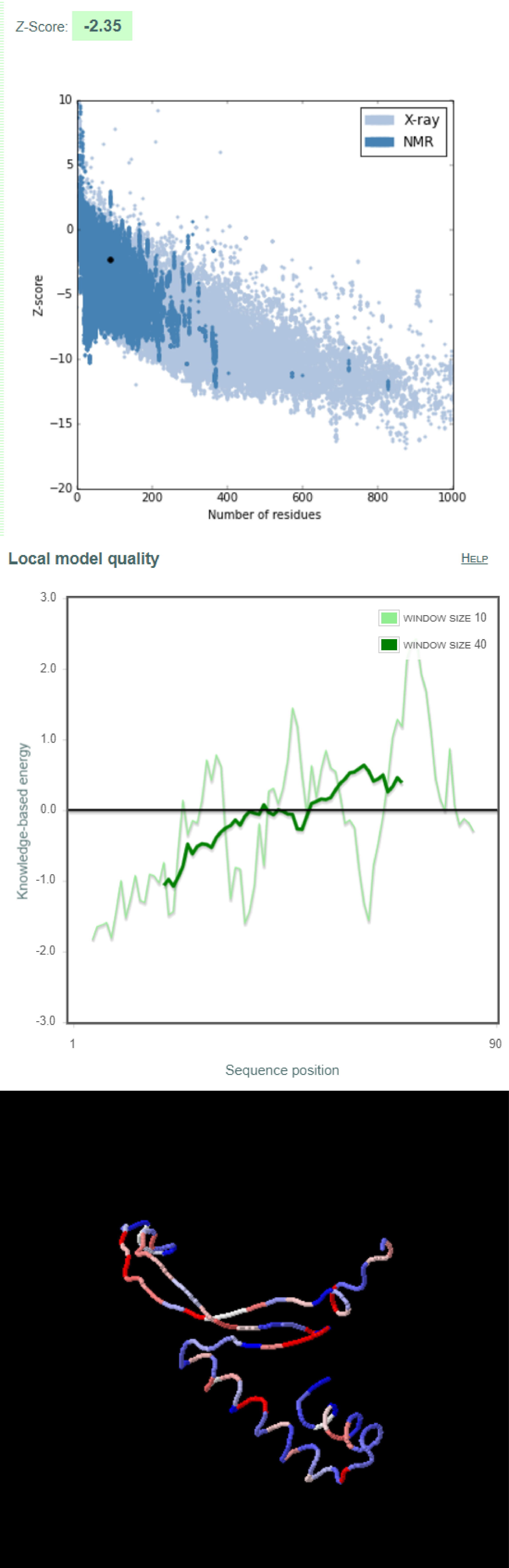

Supplement: S4 File — (ZIP) [file pone.0188037.s004.zip › C_3 v.jpg]

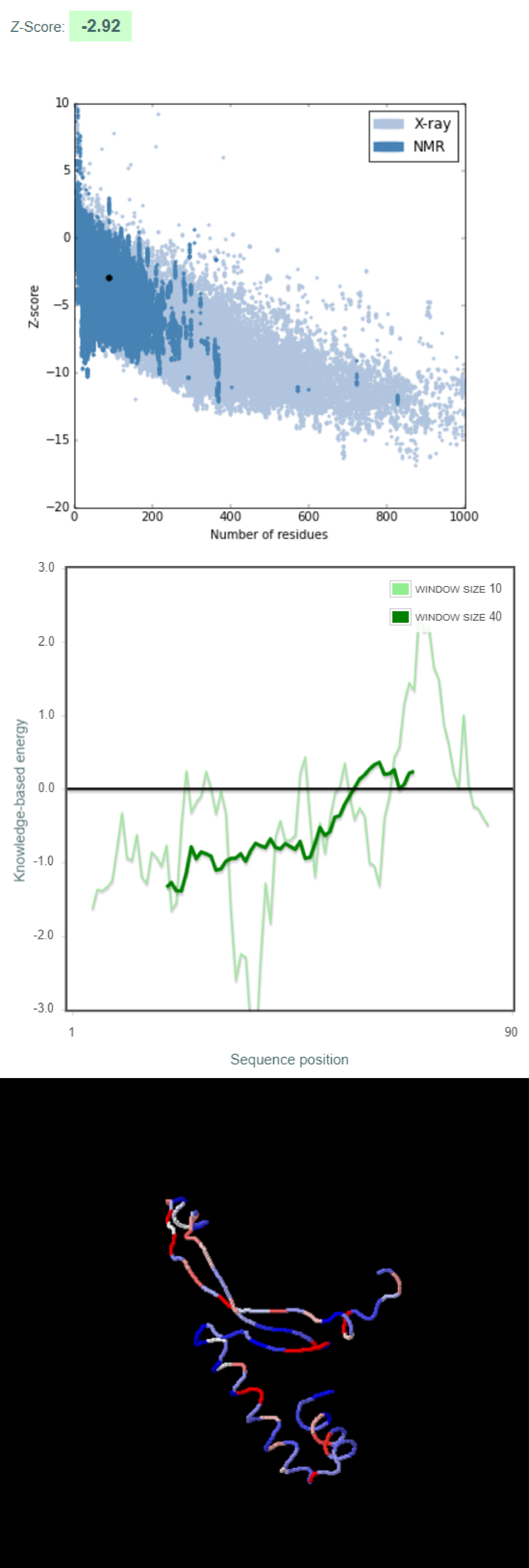

Supplement: S4 File — (ZIP) [file pone.0188037.s004.zip › C_4 v.jpg]

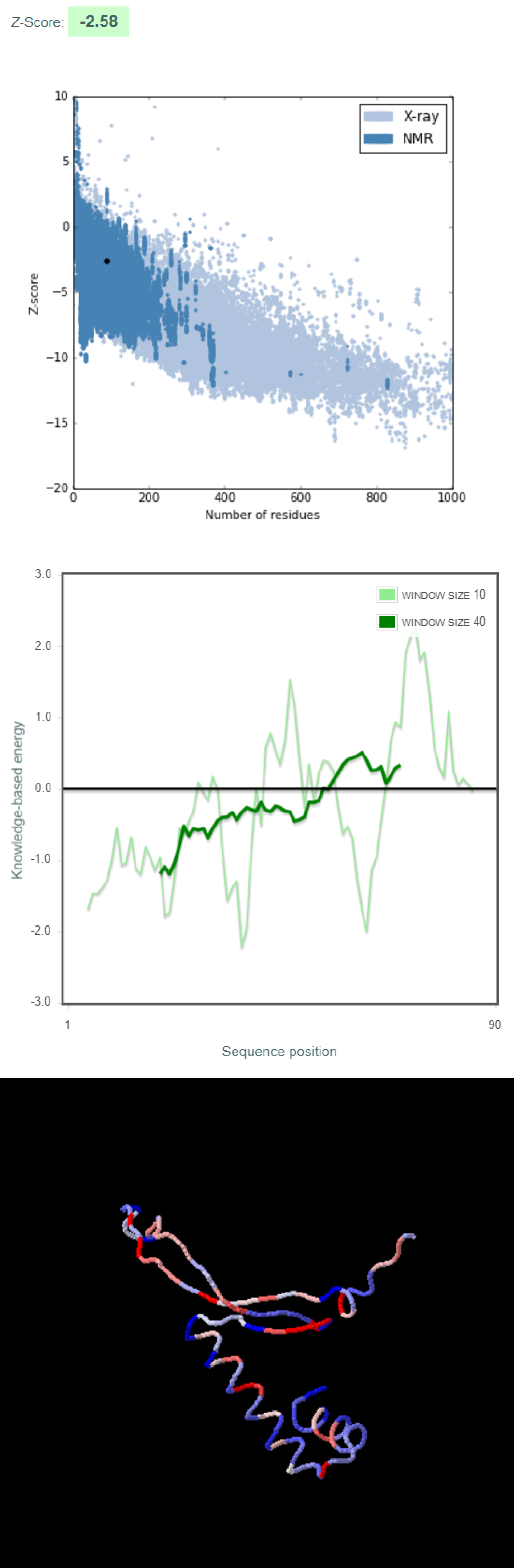

Supplement: S4 File — (ZIP) [file pone.0188037.s004.zip › C_5 v.jpg]

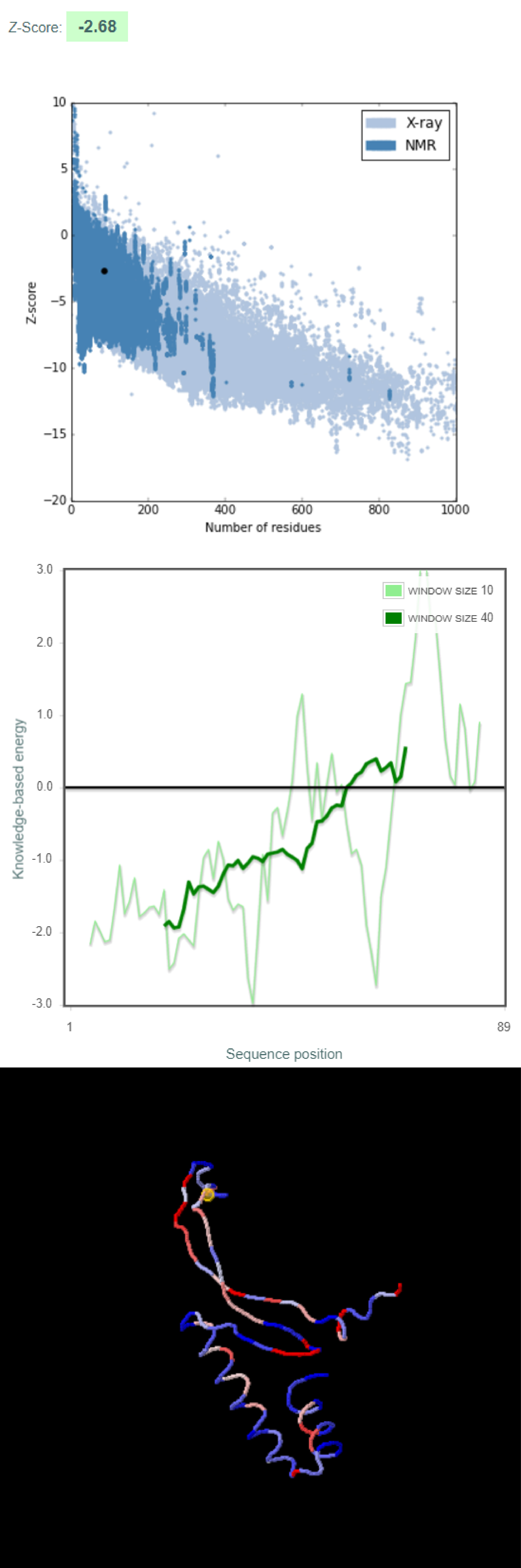

Supplement: S4 File — (ZIP) [file pone.0188037.s004.zip › C_6 v.jpg]

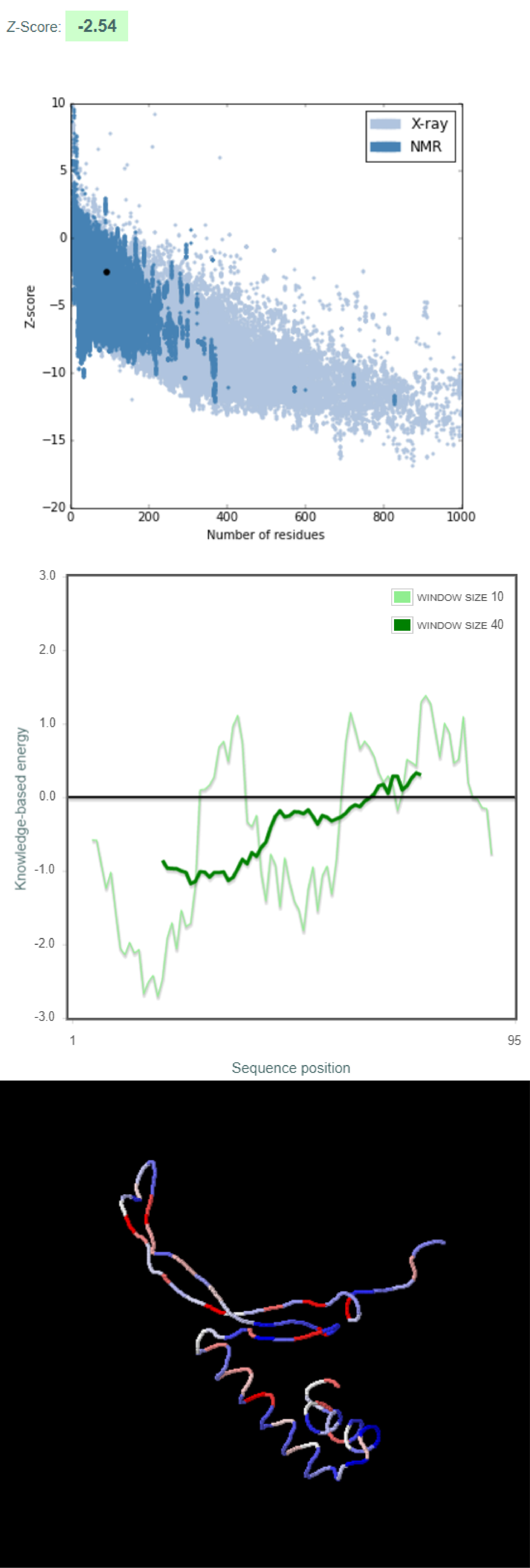

Supplement: S4 File — (ZIP) [file pone.0188037.s004.zip › D2_1 v.jpg]

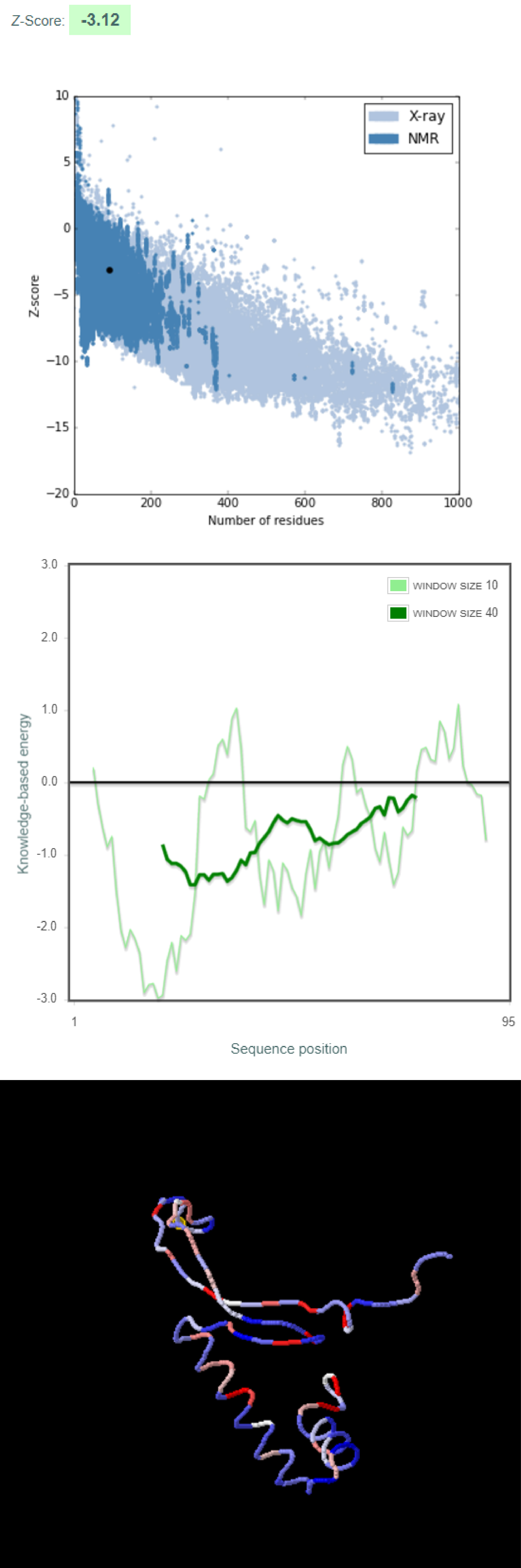

Supplement: S4 File — (ZIP) [file pone.0188037.s004.zip › D2_2 v.jpg]

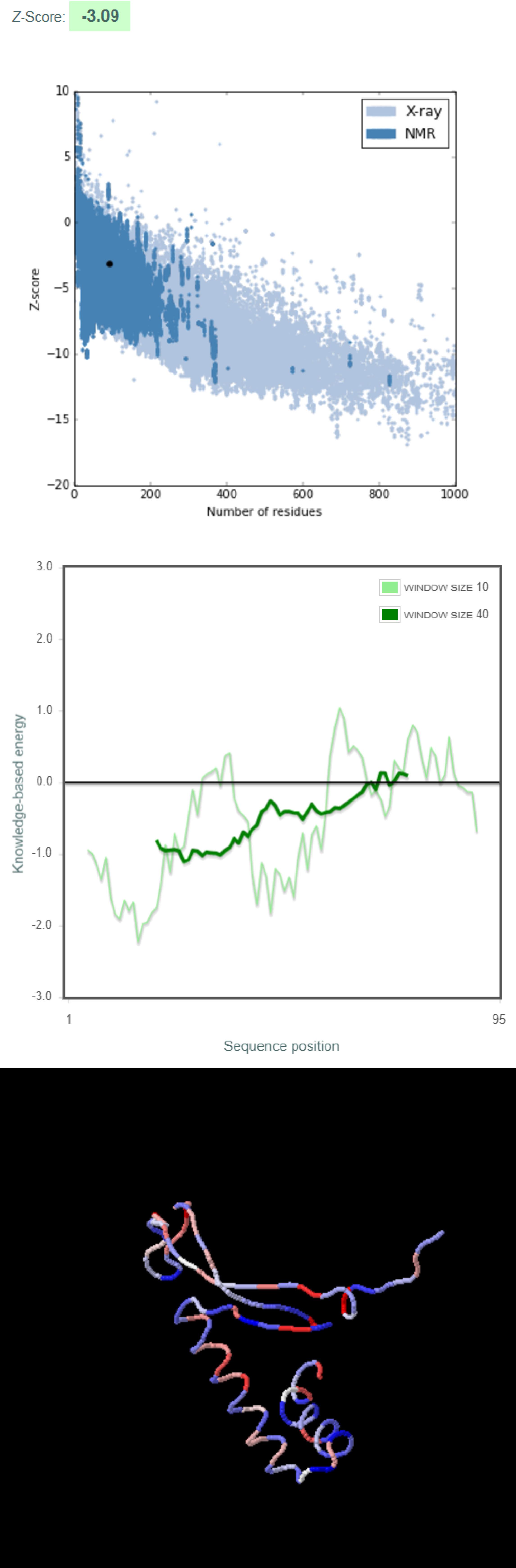

Supplement: S4 File — (ZIP) [file pone.0188037.s004.zip › D2_3 v.jpg]

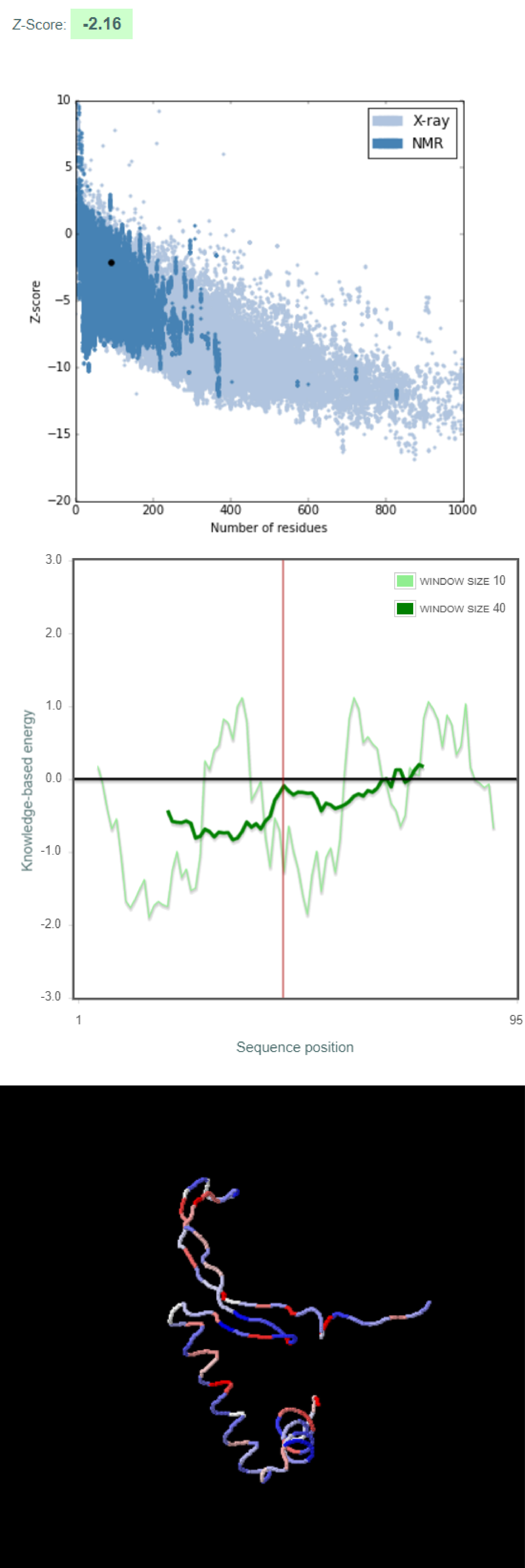

Supplement: S4 File — (ZIP) [file pone.0188037.s004.zip › D2_4 v.jpg]

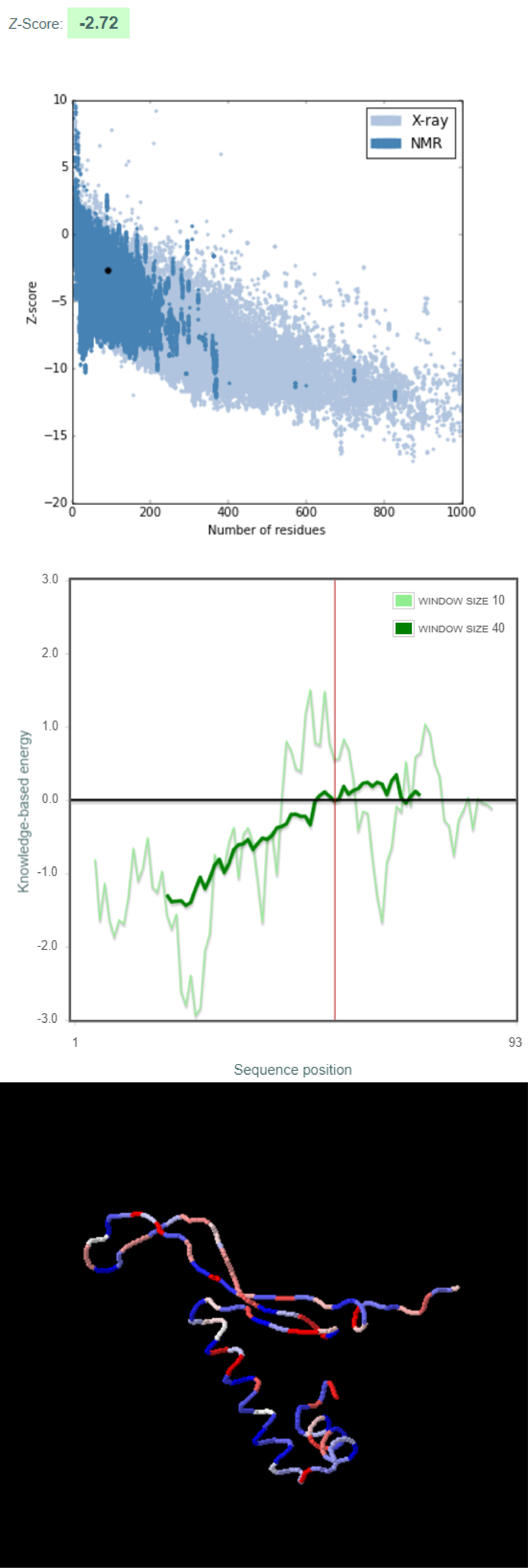

Supplement: S4 File — (ZIP) [file pone.0188037.s004.zip › D3_1 v.jpg]

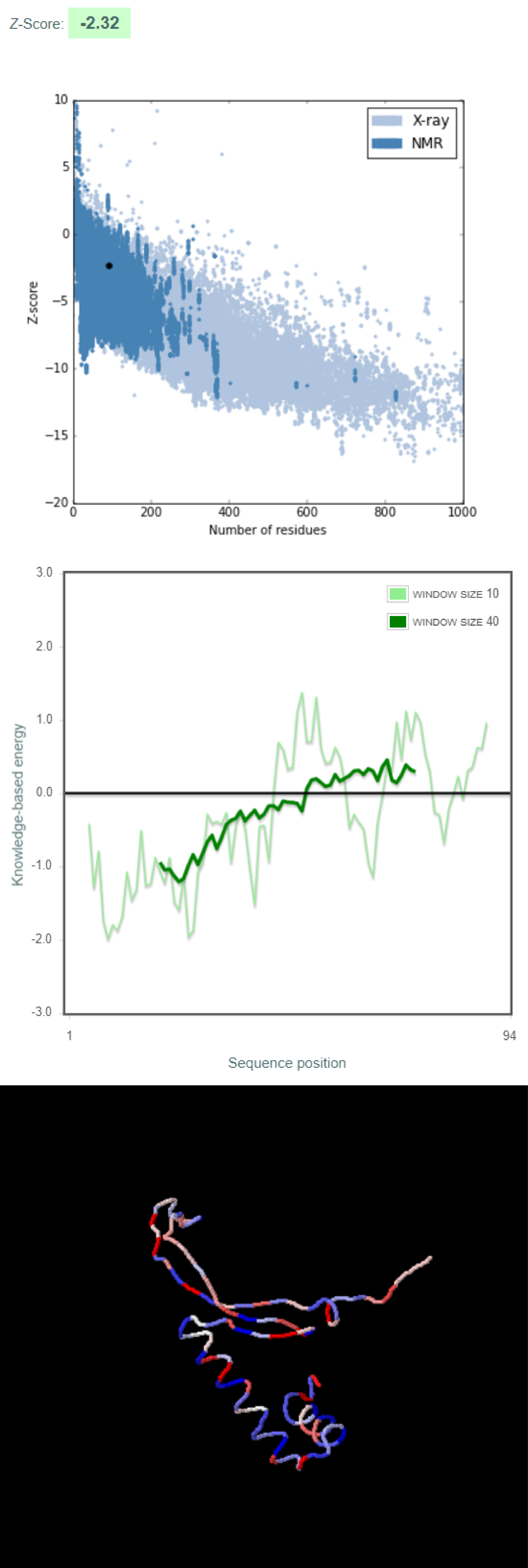

Supplement: S4 File — (ZIP) [file pone.0188037.s004.zip › D3_2 v.jpg]

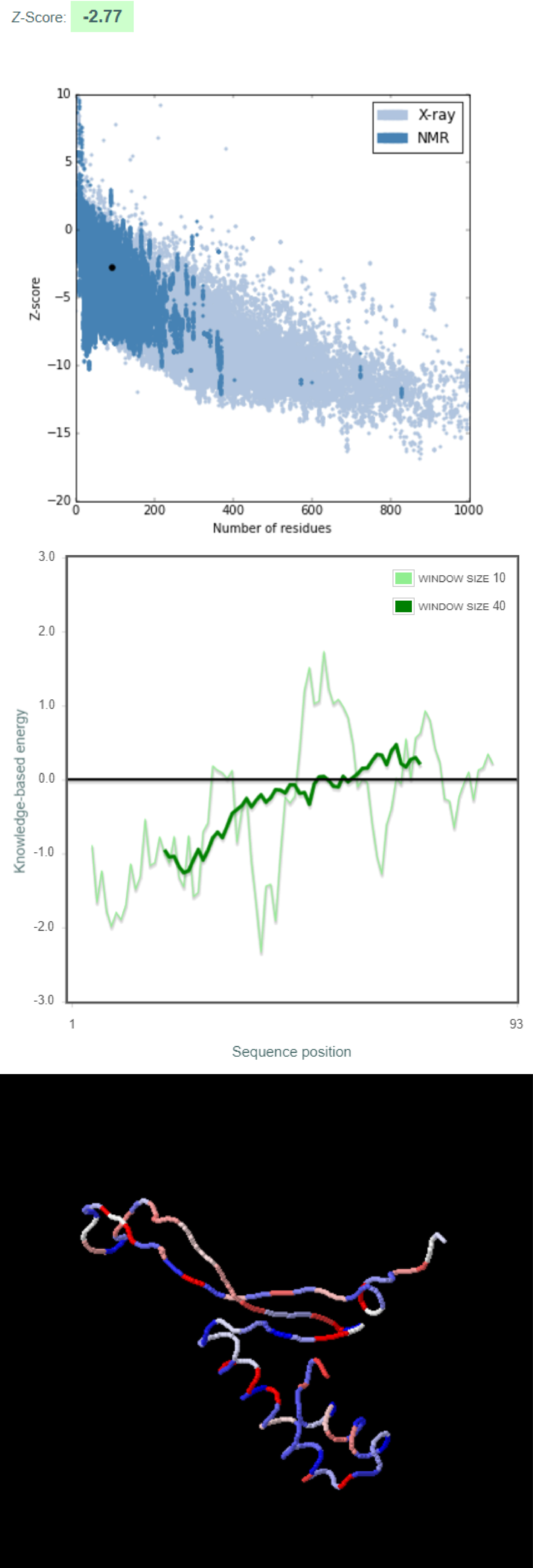

Supplement: S4 File — (ZIP) [file pone.0188037.s004.zip › D3_3 v.jpg]

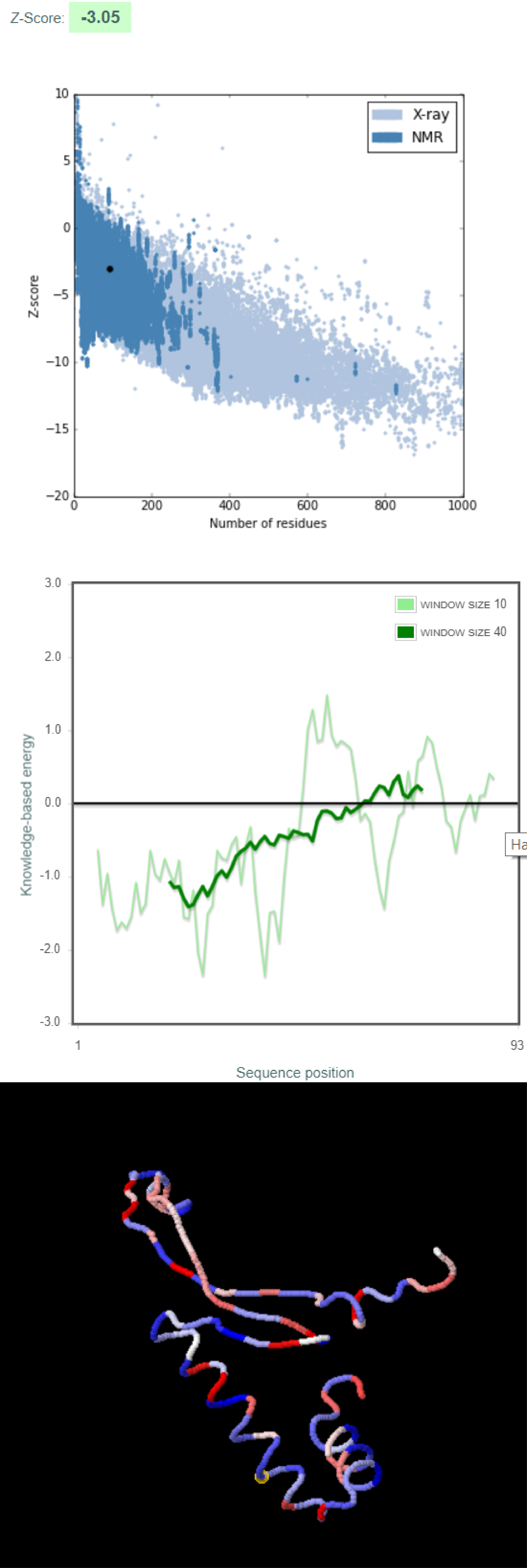

Supplement: S4 File — (ZIP) [file pone.0188037.s004.zip › D3_4 v.jpg]

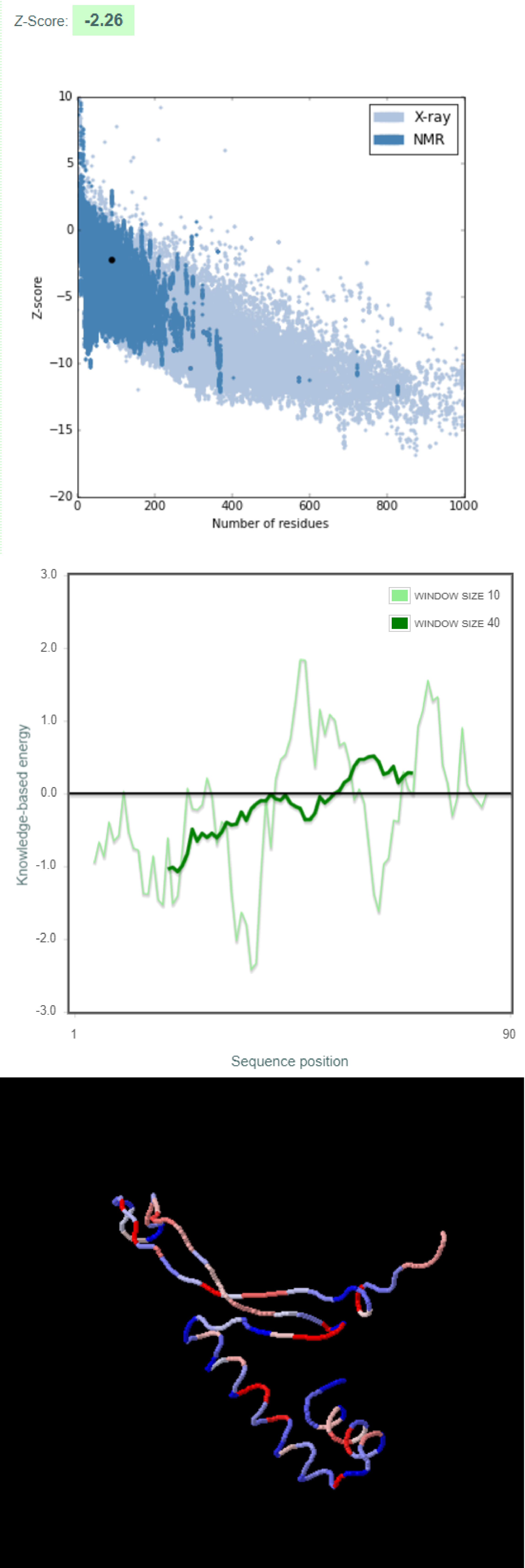

Supplement: S4 File — (ZIP) [file pone.0188037.s004.zip › D4_1 v.jpg]

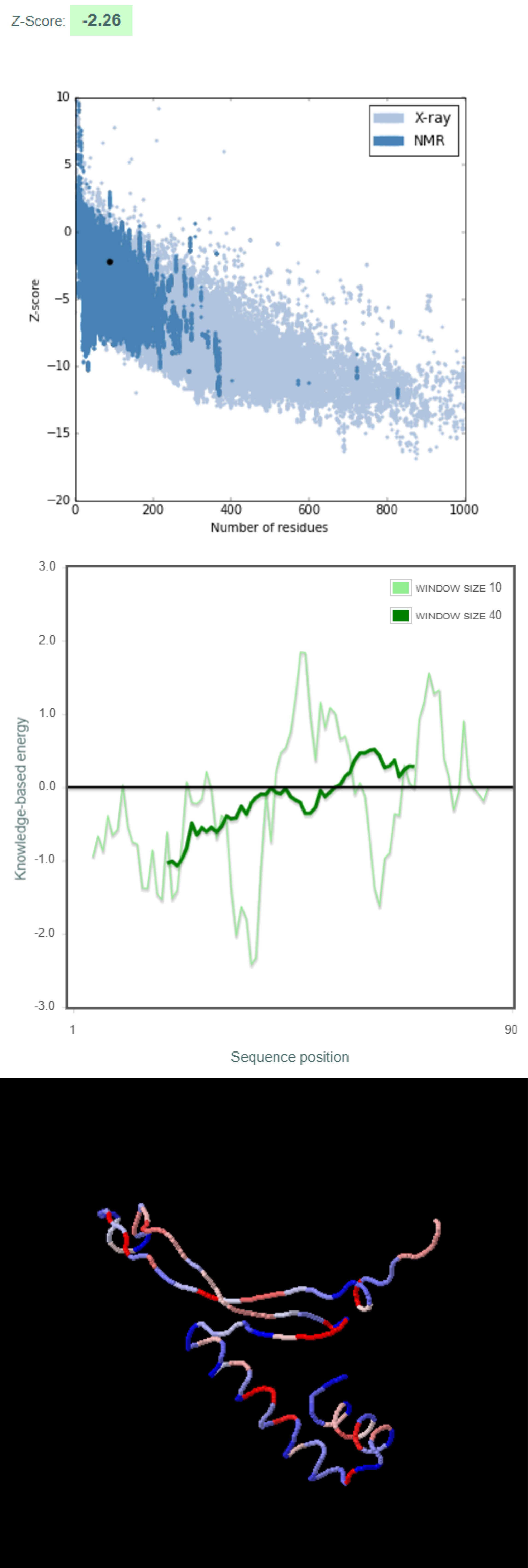

Supplement: S4 File — (ZIP) [file pone.0188037.s004.zip › D4_2 v.jpg]

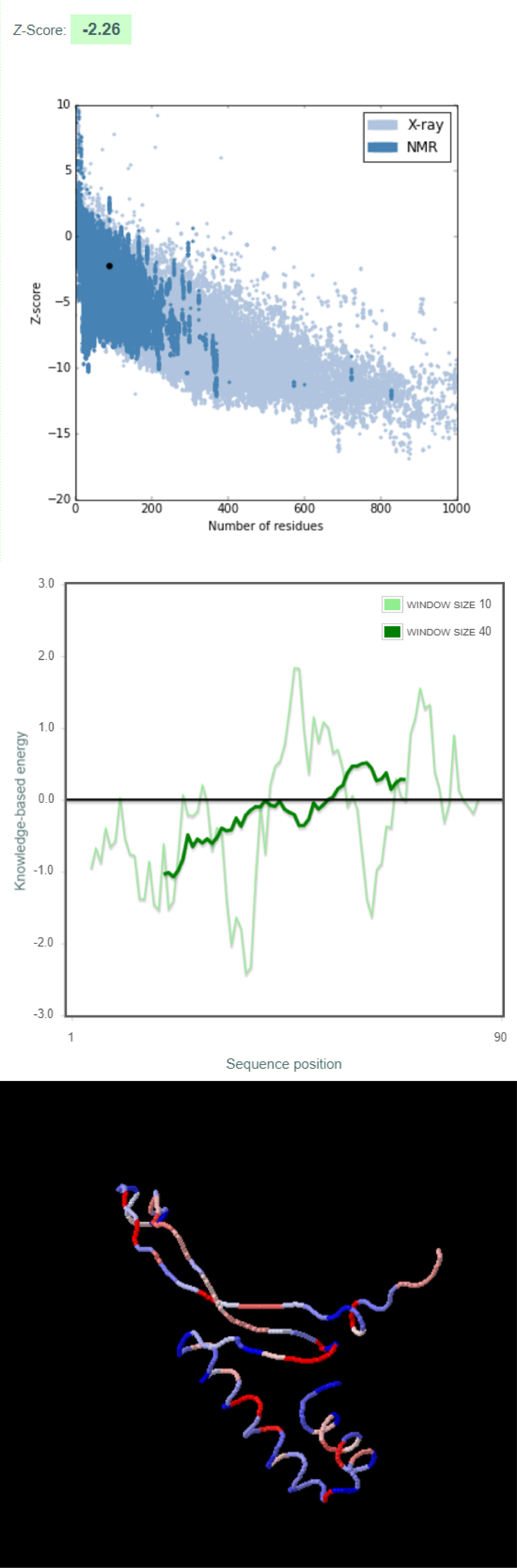

Supplement: S4 File — (ZIP) [file pone.0188037.s004.zip › D4_3 v.jpg]

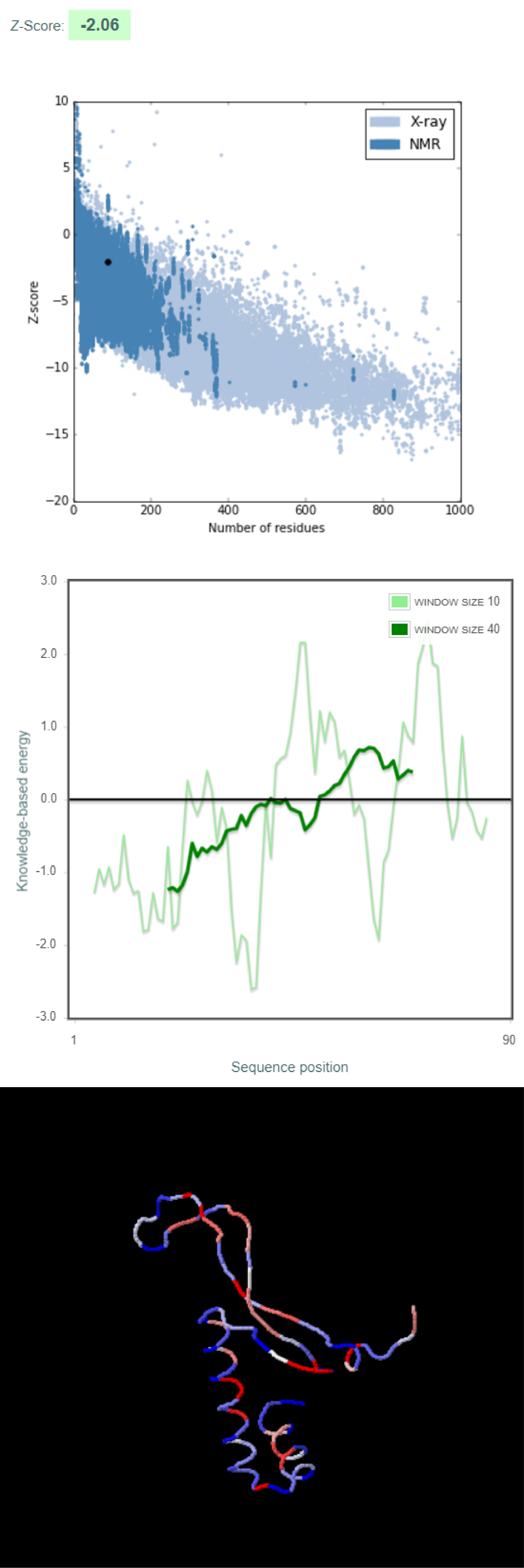

Supplement: S4 File — (ZIP) [file pone.0188037.s004.zip › D4_4 v.jpg]

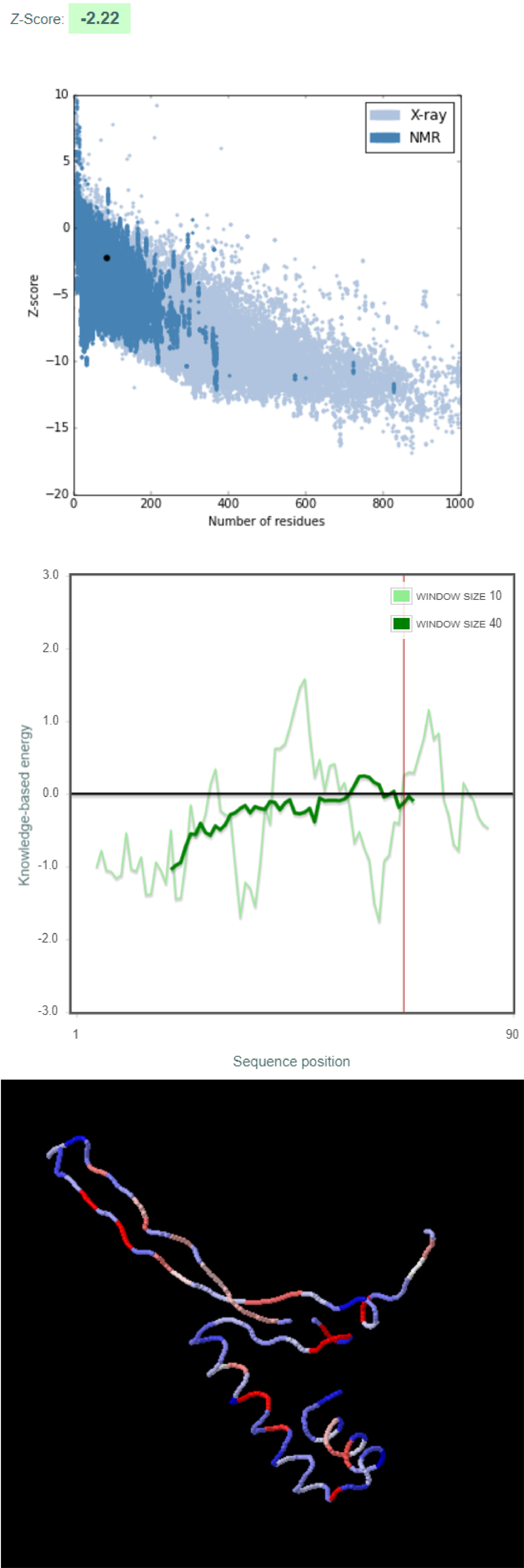

Supplement: S4 File — (ZIP) [file pone.0188037.s004.zip › D4_5 v.jpg]

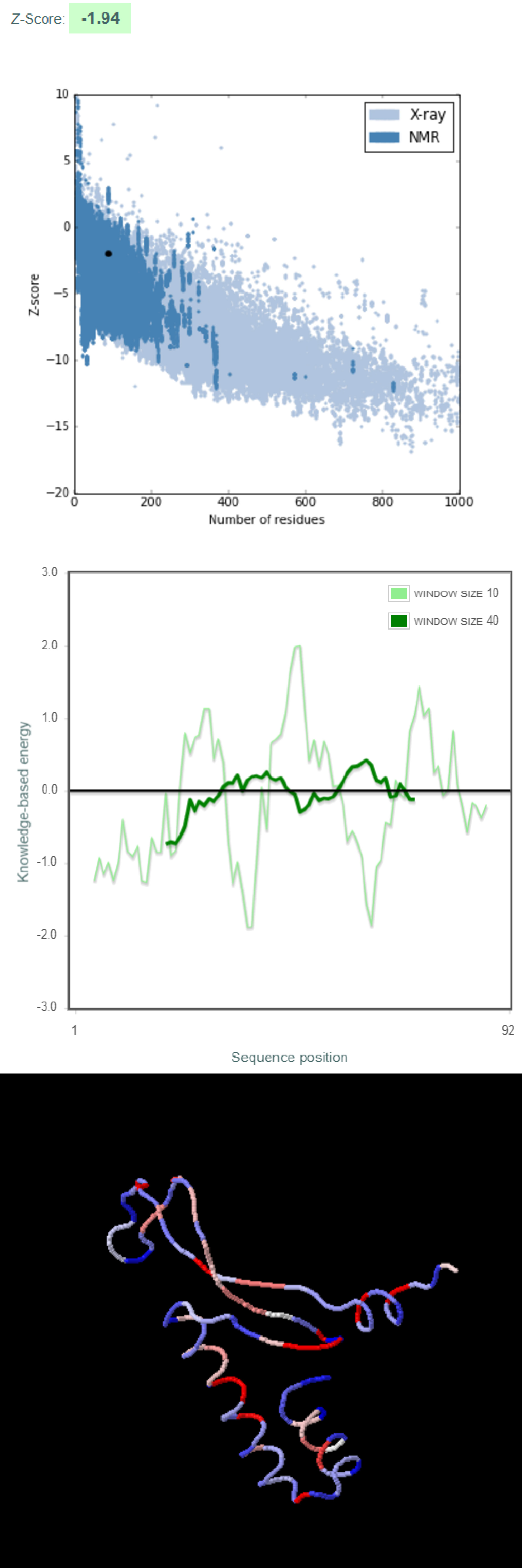

Supplement: S4 File — (ZIP) [file pone.0188037.s004.zip › D4_6 v.jpg]

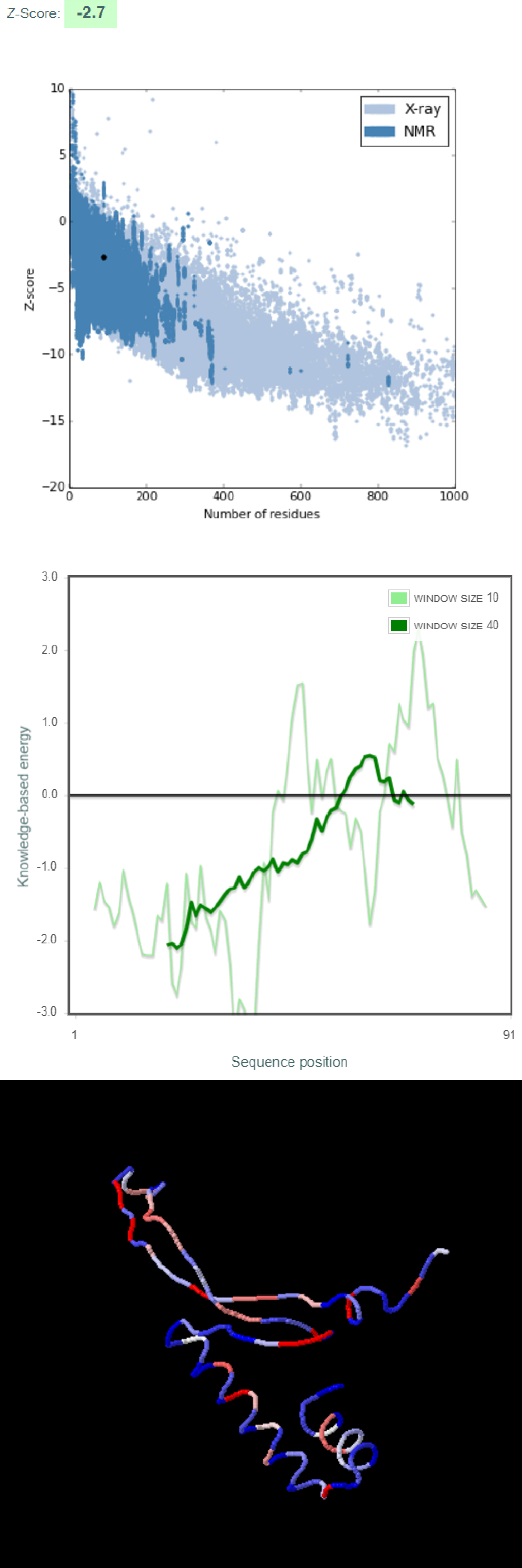

Supplement: S4 File — (ZIP) [file pone.0188037.s004.zip › D4_7 v.jpg]

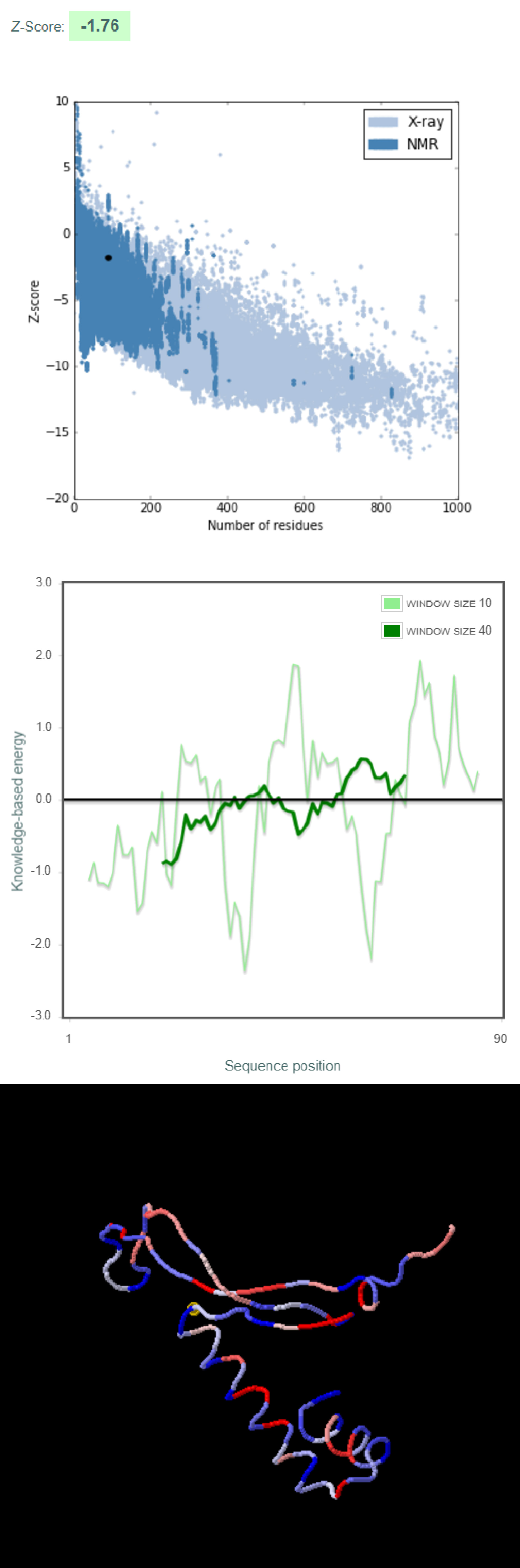

Supplement: S4 File — (ZIP) [file pone.0188037.s004.zip › D4_8 v.jpg]

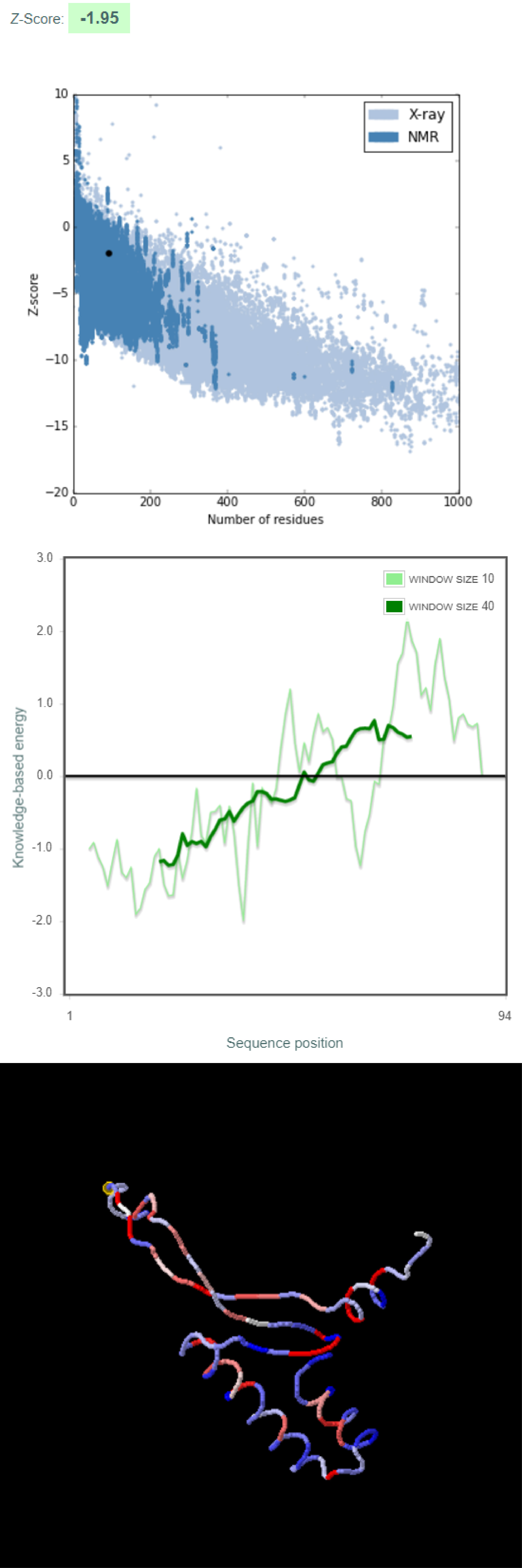

Supplement: S4 File — (ZIP) [file pone.0188037.s004.zip › D_1 v.jpg]

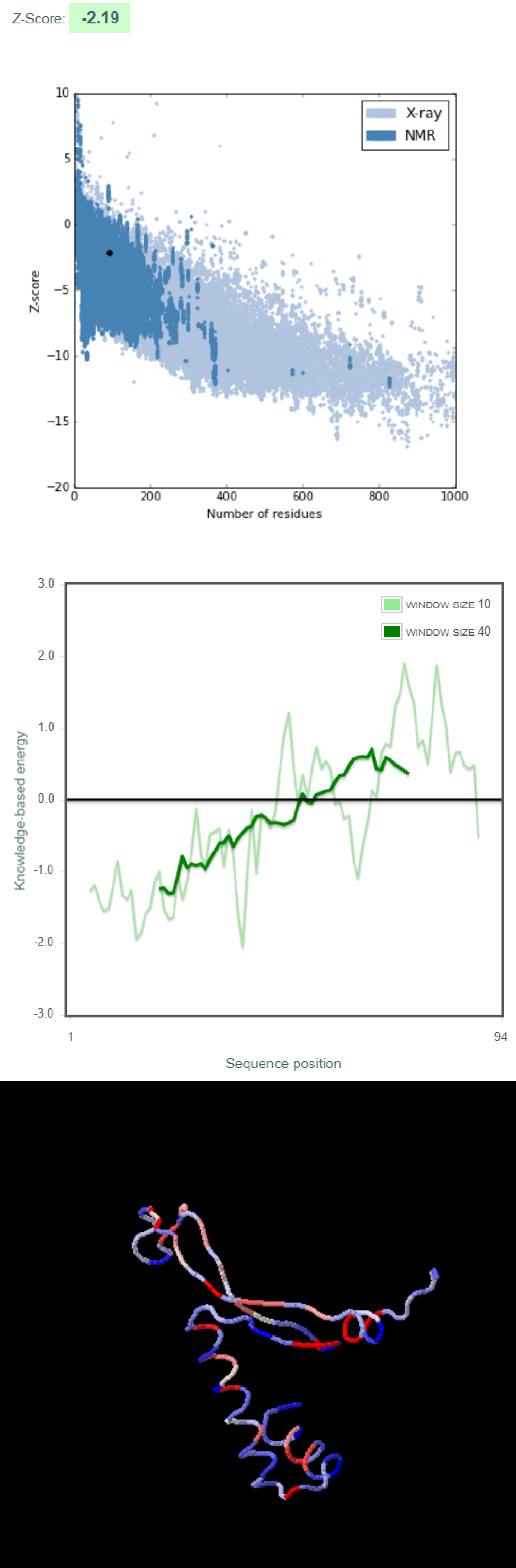

Supplement: S4 File — (ZIP) [file pone.0188037.s004.zip › D_2 v.jpg]

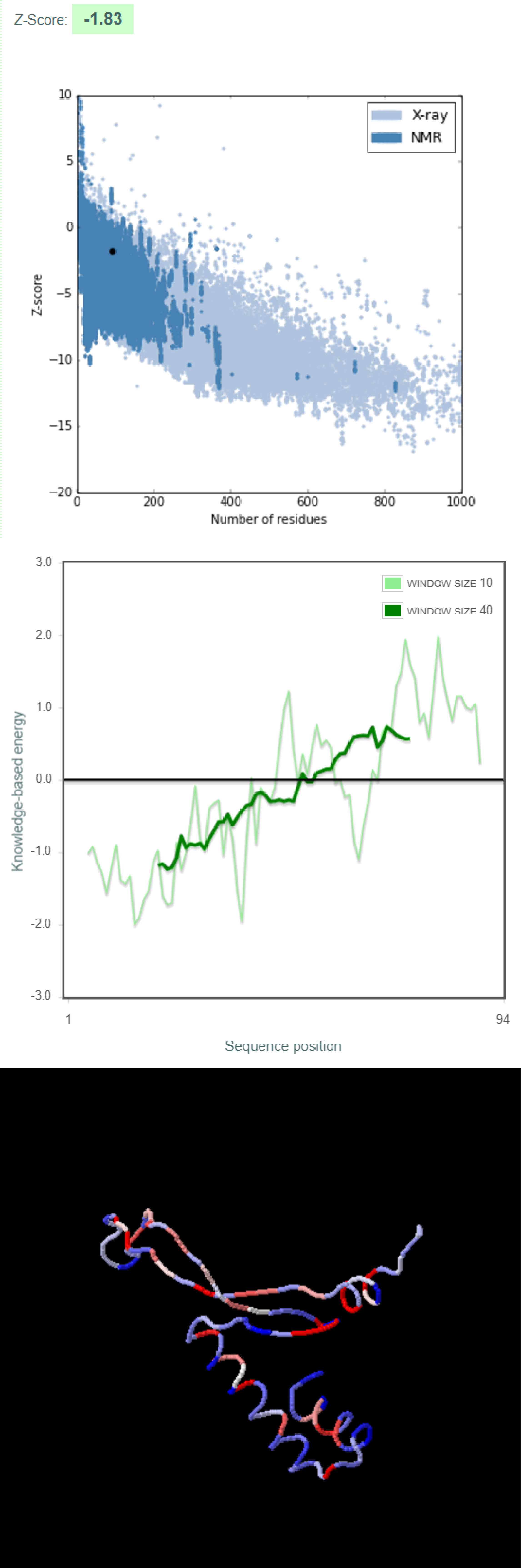

Supplement: S4 File — (ZIP) [file pone.0188037.s004.zip › D_3 v.jpg]

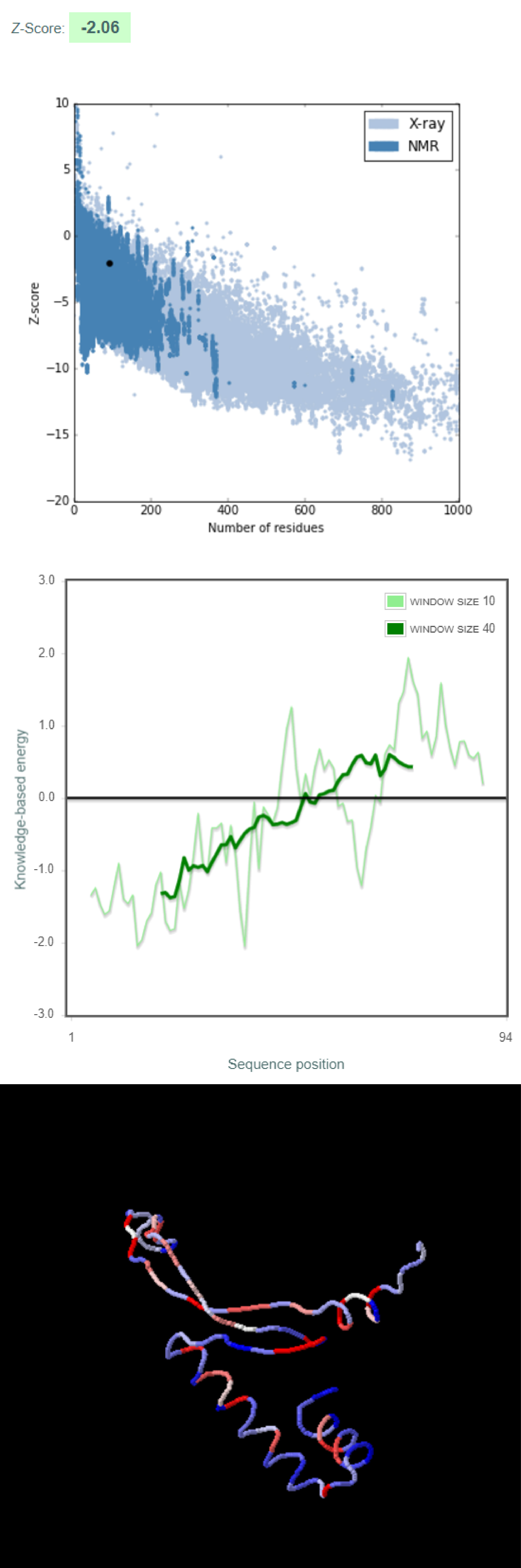

Supplement: S4 File — (ZIP) [file pone.0188037.s004.zip › D_4 v.jpg]

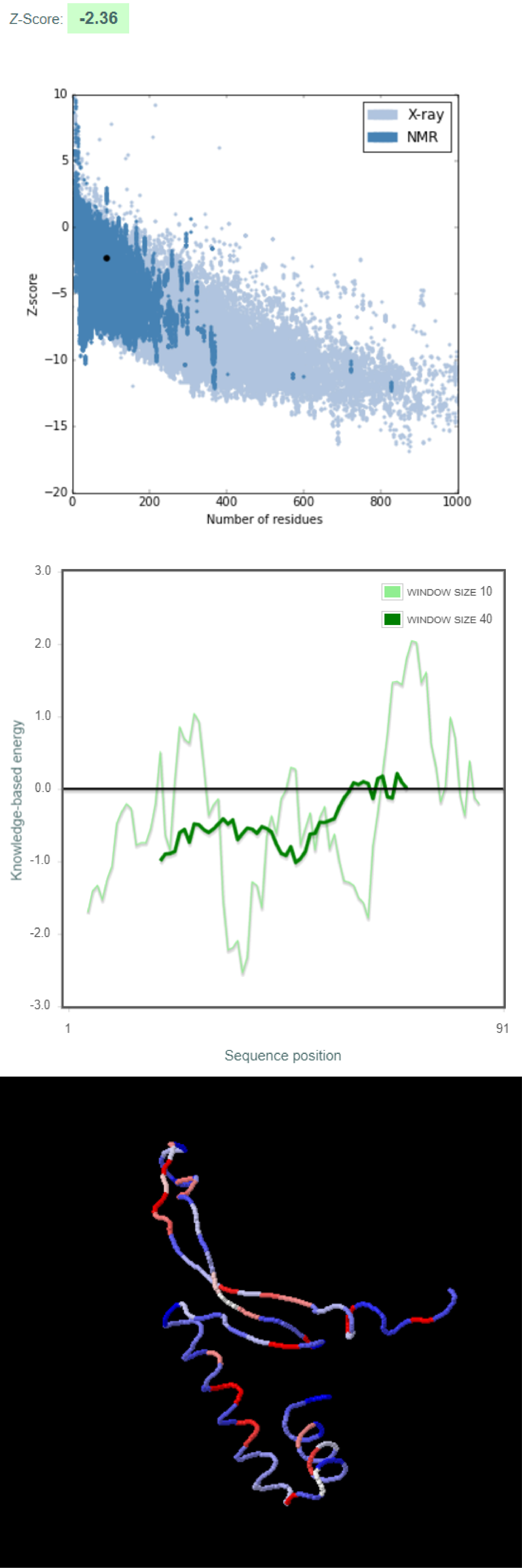

Supplement: S4 File — (ZIP) [file pone.0188037.s004.zip › E2_1 v.jpg]

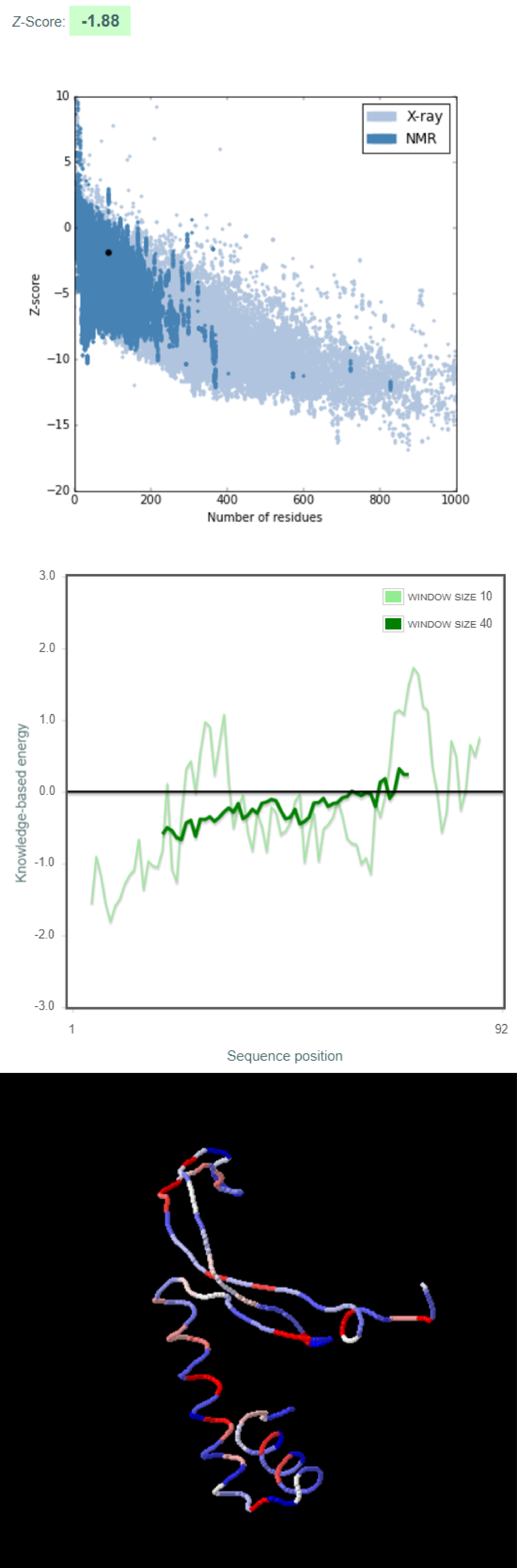

Supplement: S4 File — (ZIP) [file pone.0188037.s004.zip › E2_2 v.jpg]

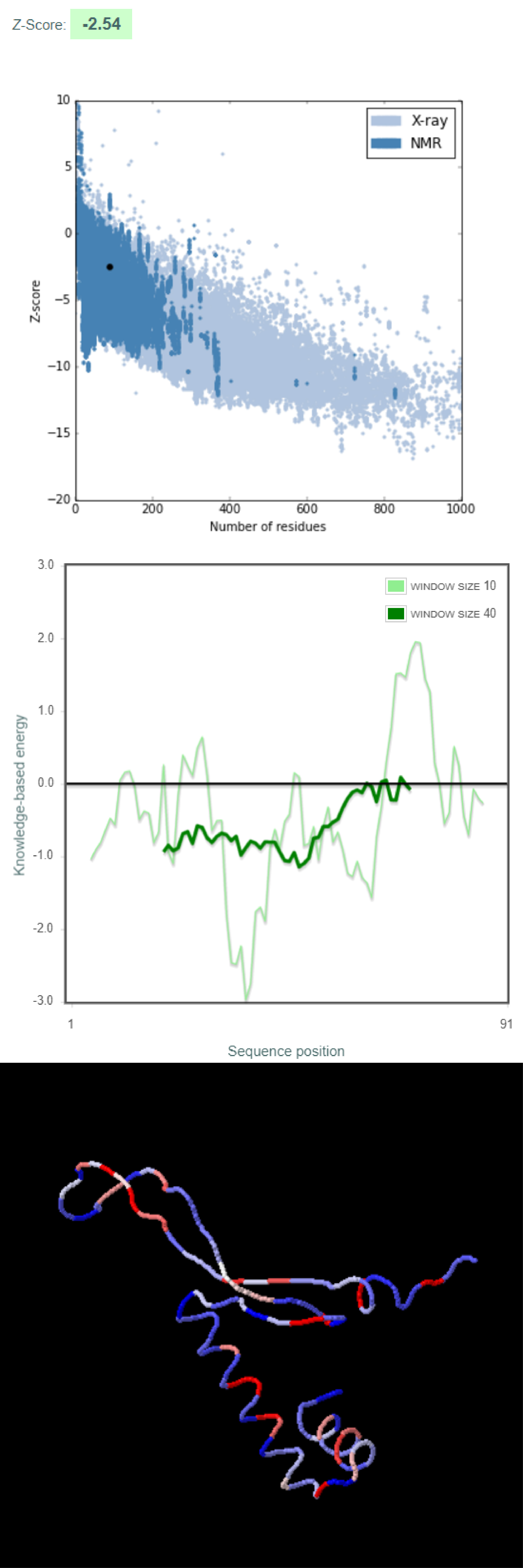

Supplement: S4 File — (ZIP) [file pone.0188037.s004.zip › E2_3 v.jpg]

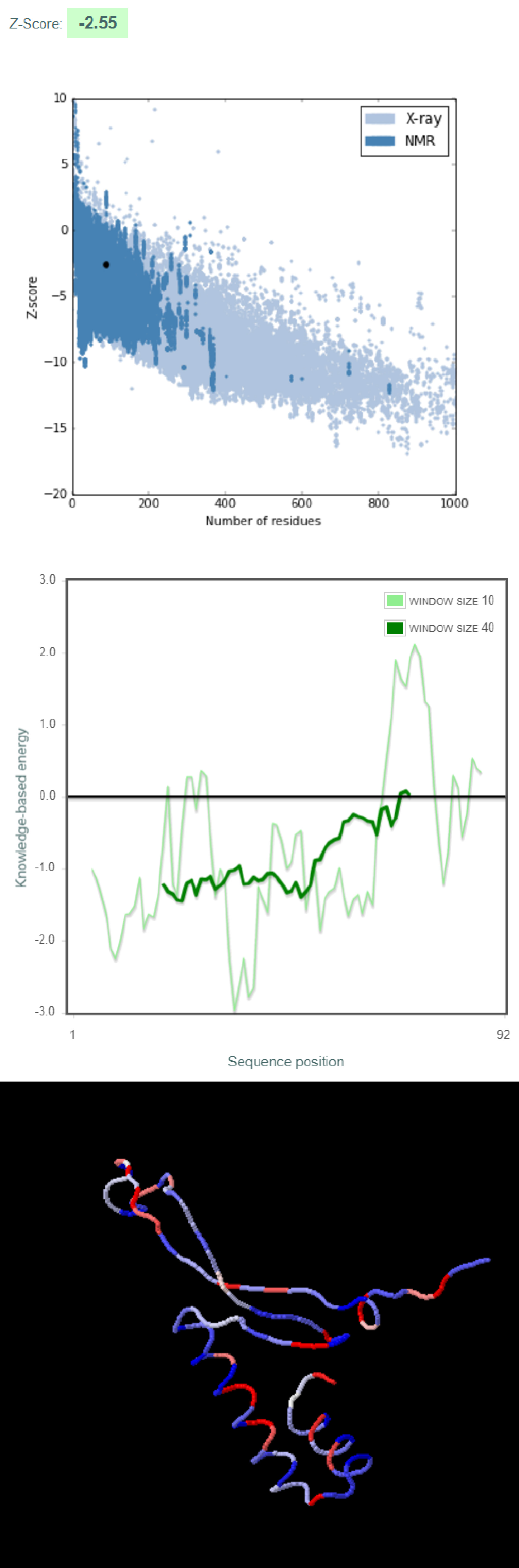

Supplement: S4 File — (ZIP) [file pone.0188037.s004.zip › E2_4 v.jpg]

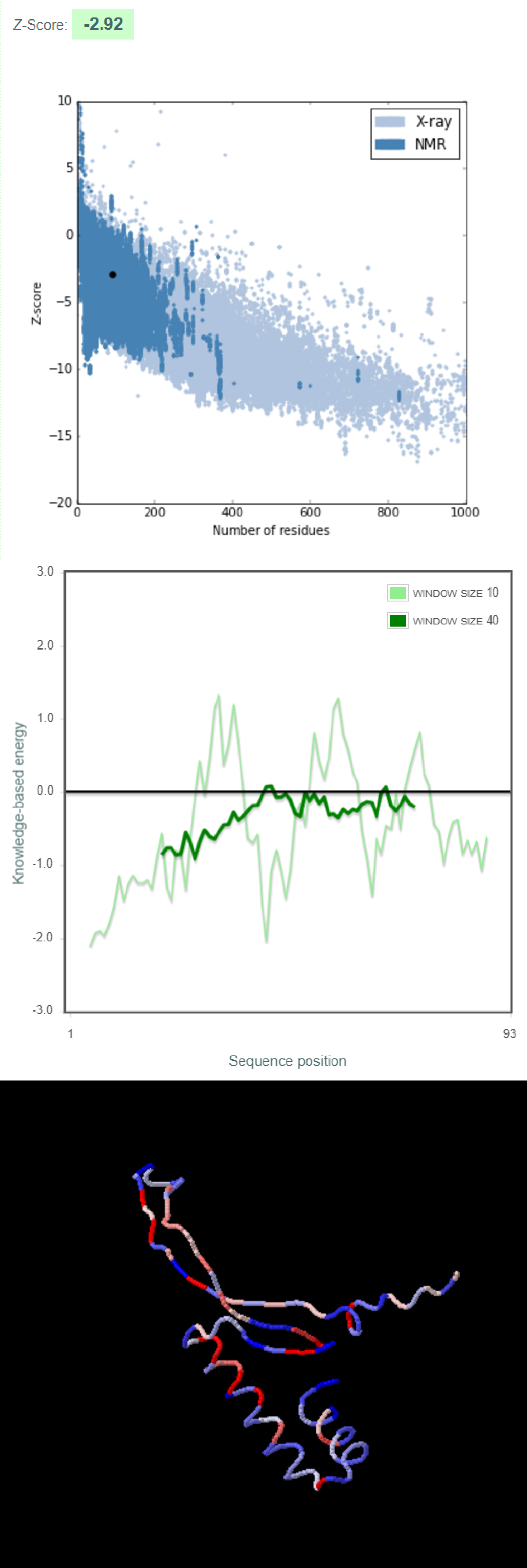

Supplement: S4 File — (ZIP) [file pone.0188037.s004.zip › E3_1 v.jpg]

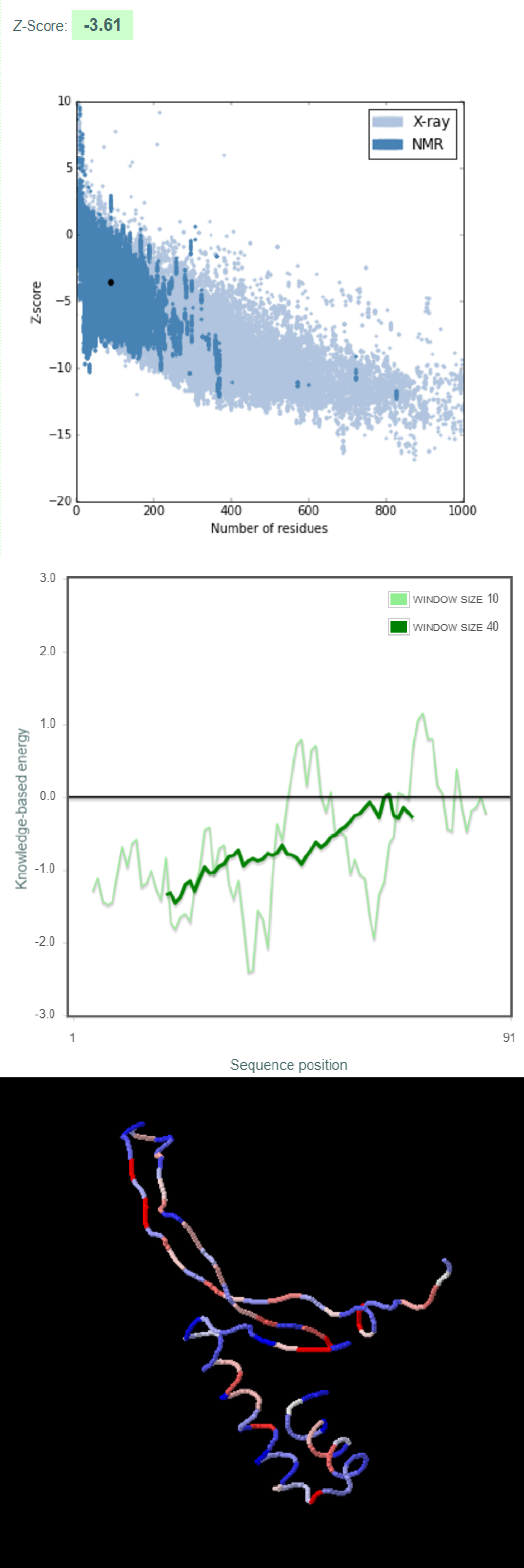

Supplement: S4 File — (ZIP) [file pone.0188037.s004.zip › E3_10 v.jpg]

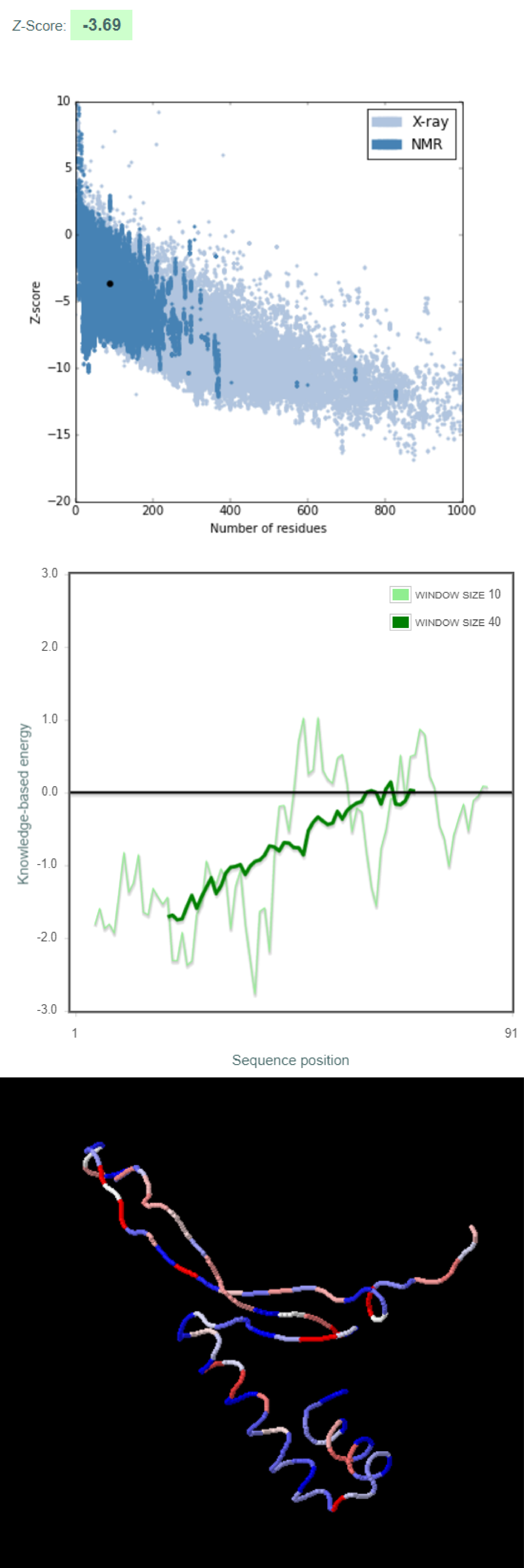

Supplement: S4 File — (ZIP) [file pone.0188037.s004.zip › E3_11 v.jpg]

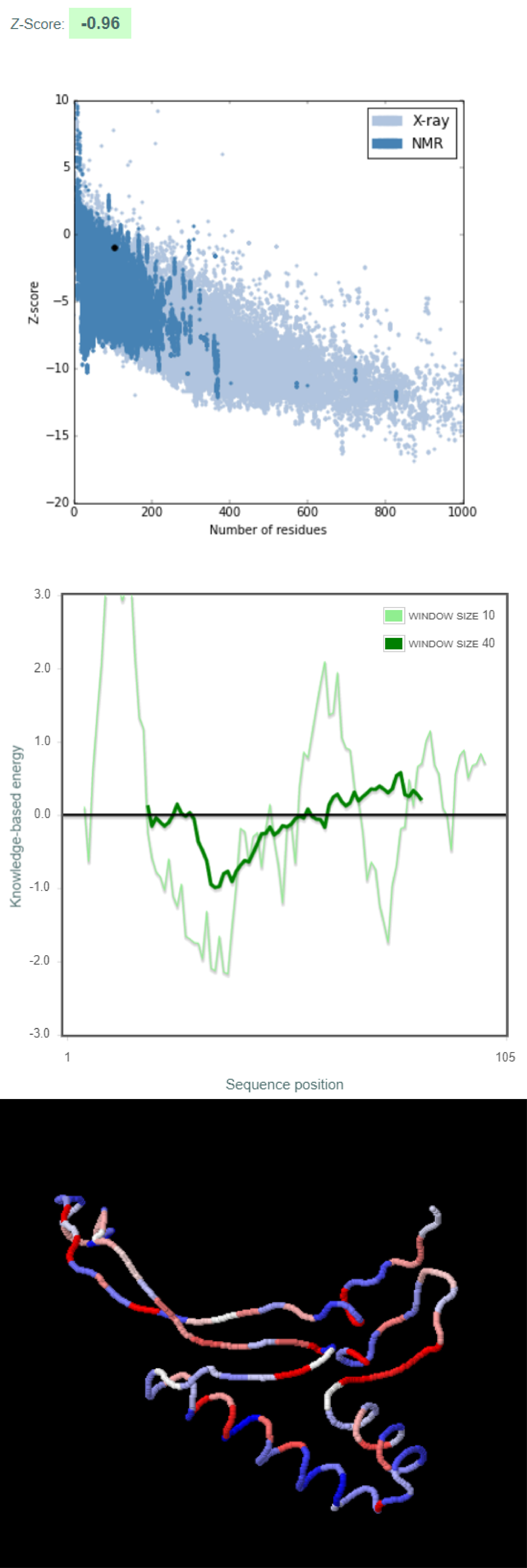

Supplement: S4 File — (ZIP) [file pone.0188037.s004.zip › E3_12 v.jpg]

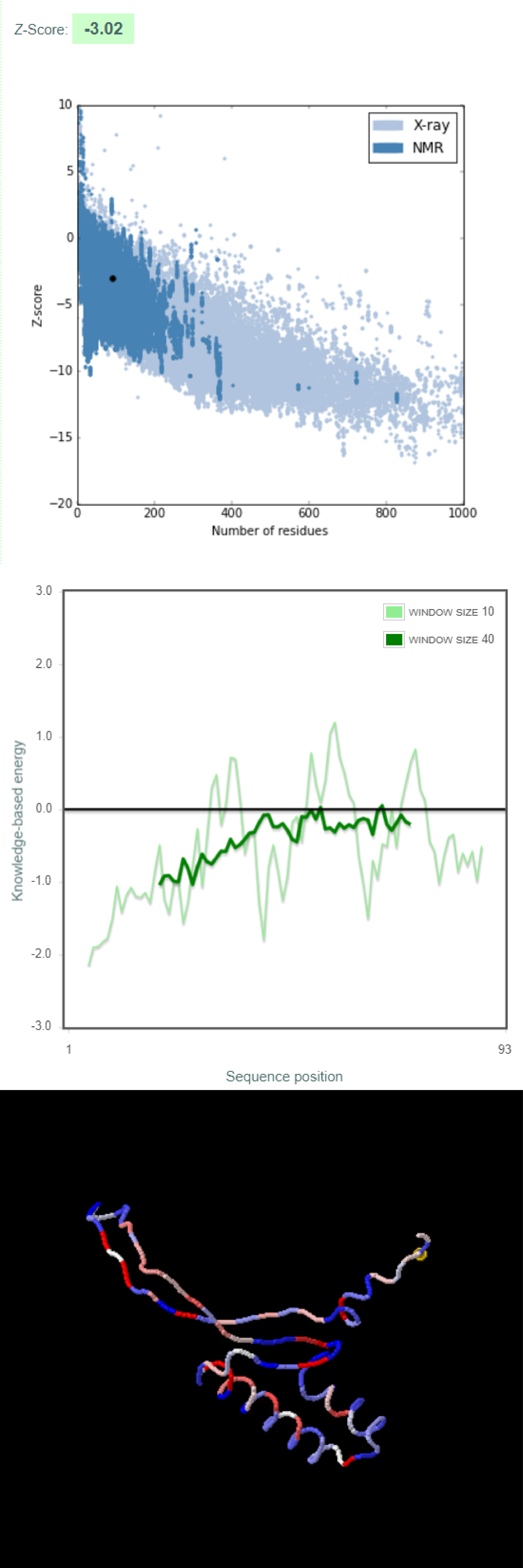

Supplement: S4 File — (ZIP) [file pone.0188037.s004.zip › E3_2 v.jpg]

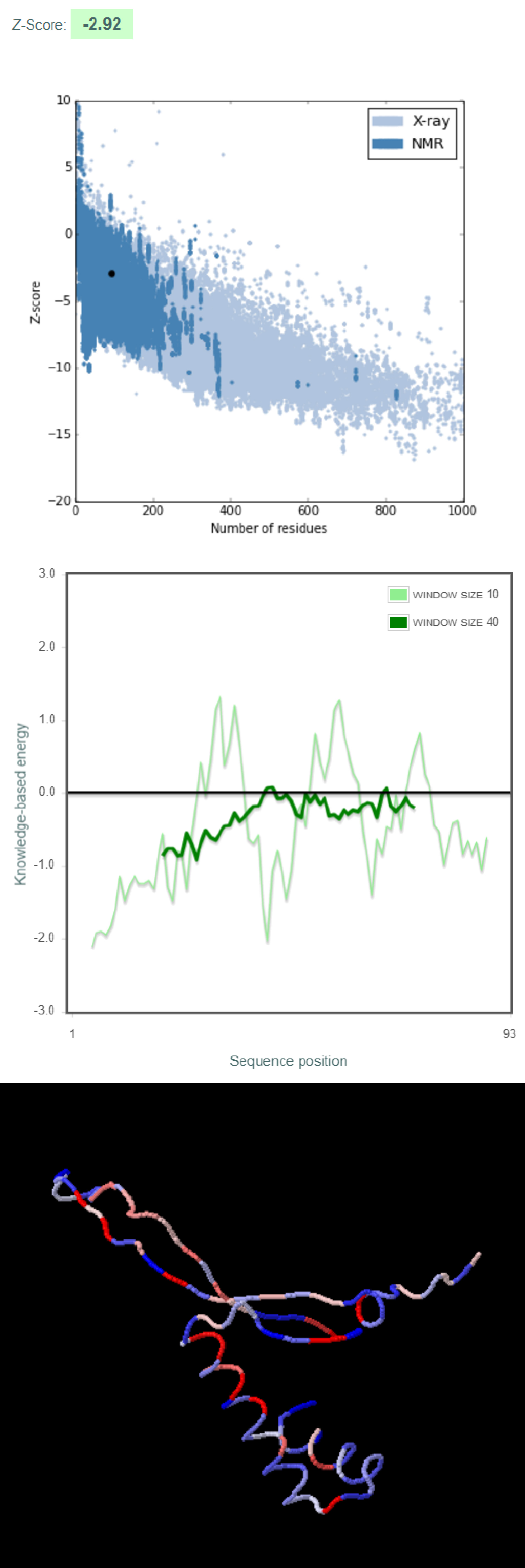

Supplement: S4 File — (ZIP) [file pone.0188037.s004.zip › E3_3 v.jpg]

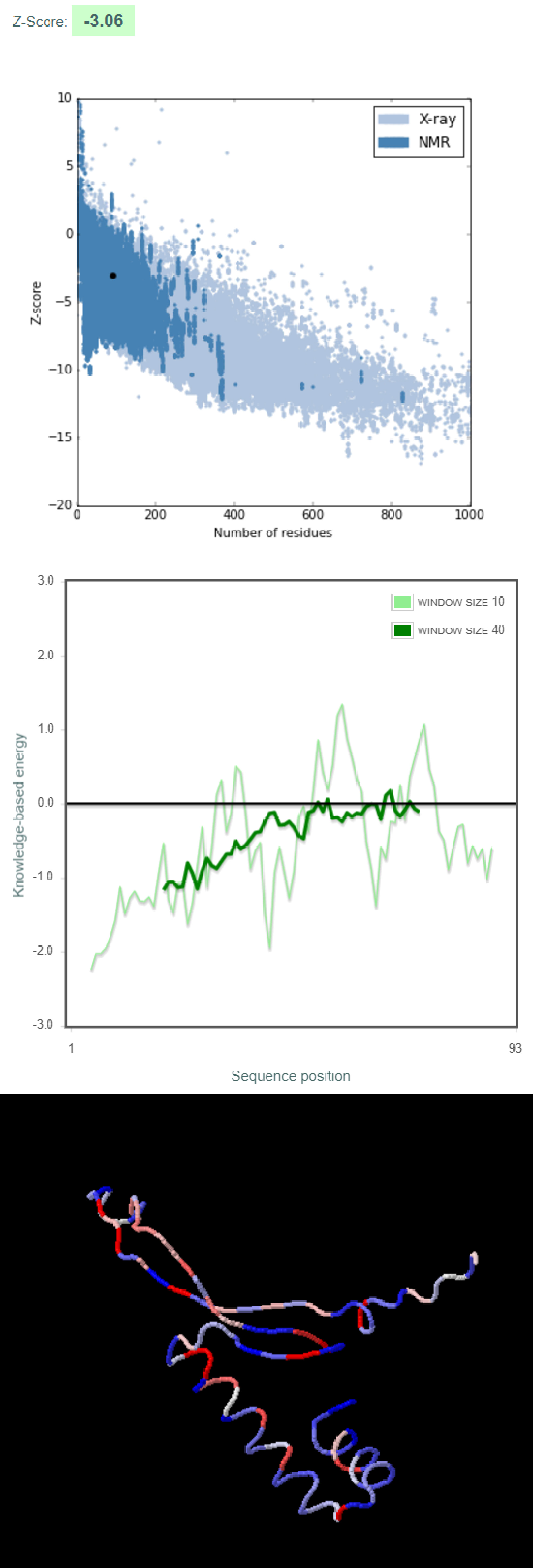

Supplement: S4 File — (ZIP) [file pone.0188037.s004.zip › E3_4 v.jpg]

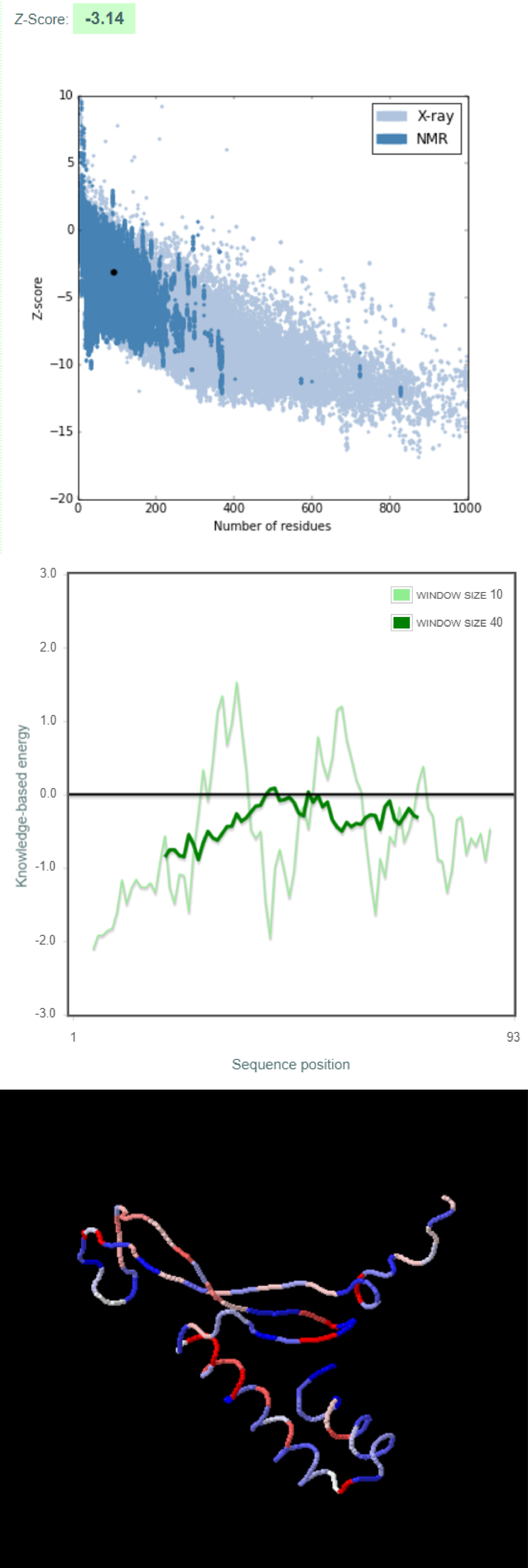

Supplement: S4 File — (ZIP) [file pone.0188037.s004.zip › E3_5 v.jpg]

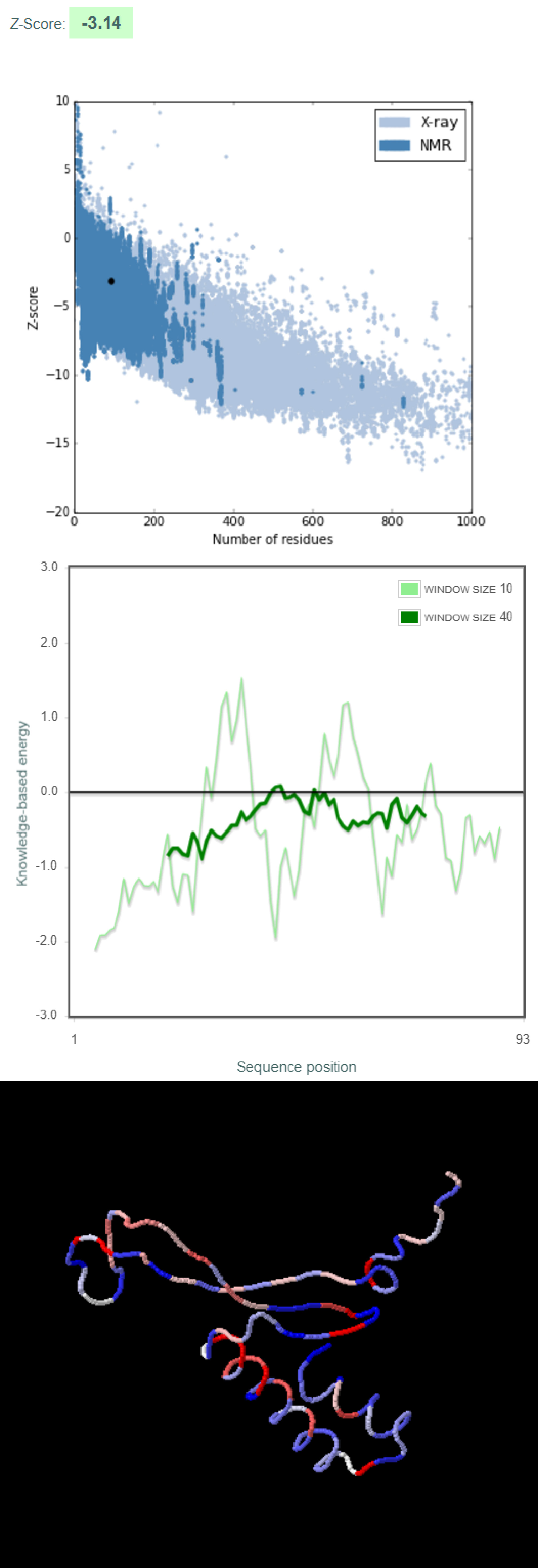

Supplement: S4 File — (ZIP) [file pone.0188037.s004.zip › E3_6 v.jpg]

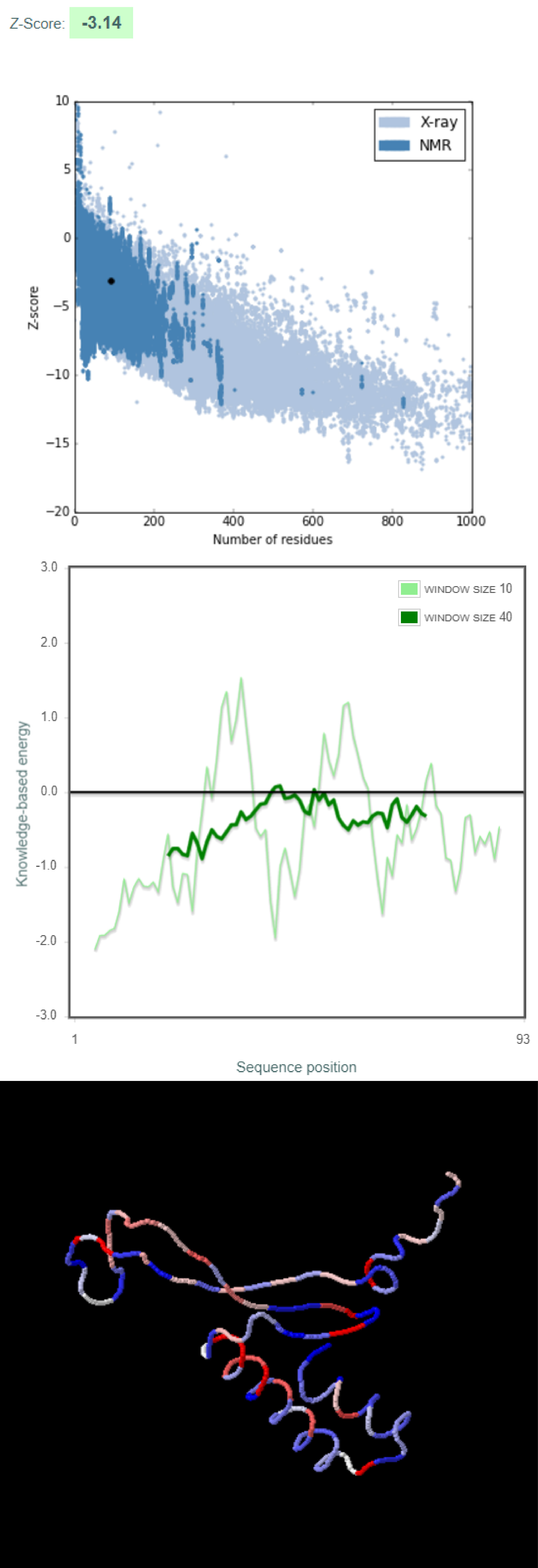

Supplement: S4 File — (ZIP) [file pone.0188037.s004.zip › E3_7 v.jpg]

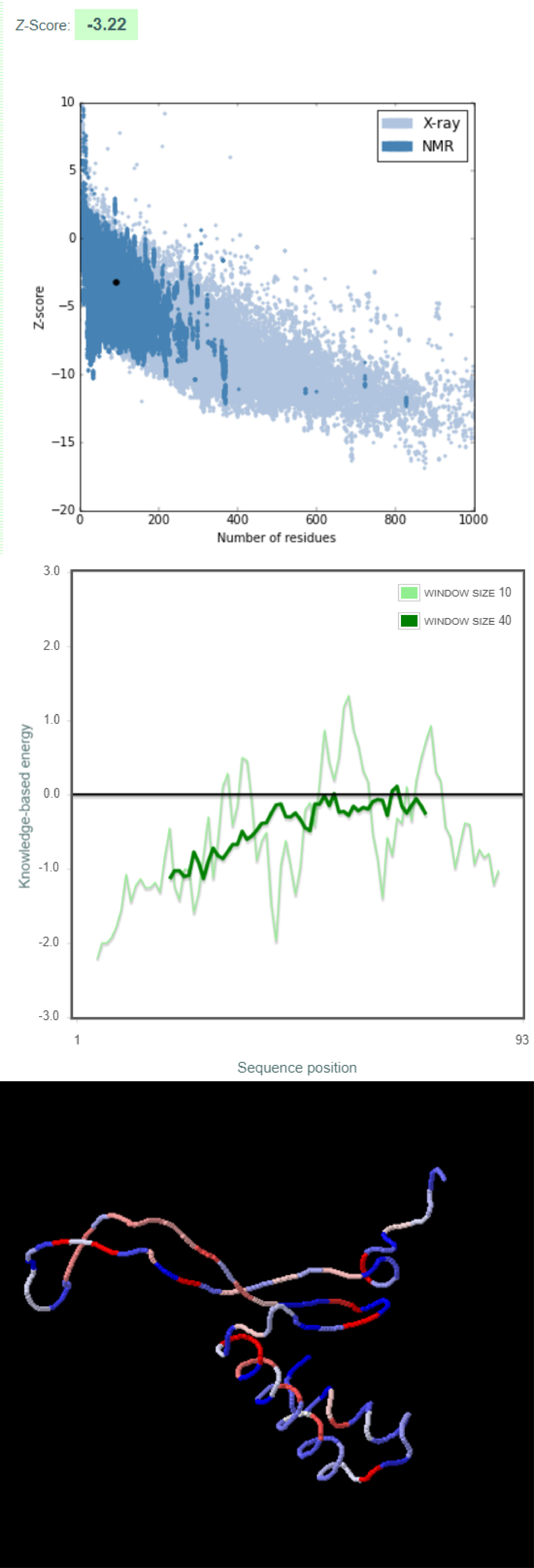

Supplement: S4 File — (ZIP) [file pone.0188037.s004.zip › E3_8 v.jpg]

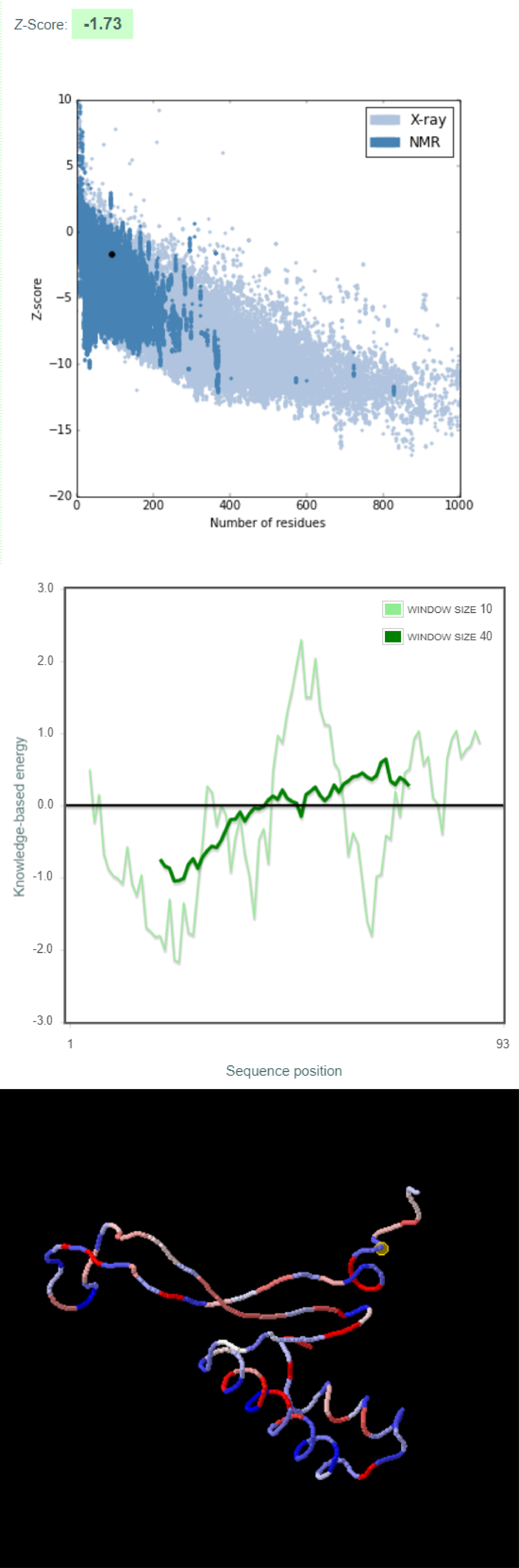

Supplement: S4 File — (ZIP) [file pone.0188037.s004.zip › E3_9 v.jpg]

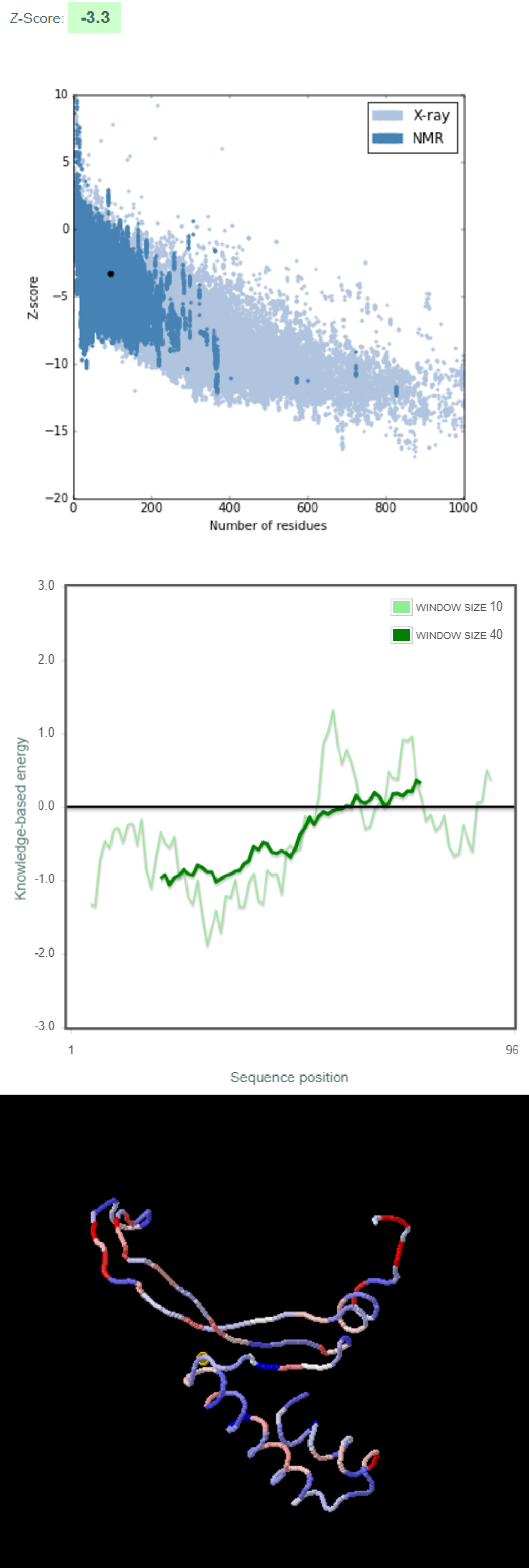

Supplement: S4 File — (ZIP) [file pone.0188037.s004.zip › E4_1 v.jpg]

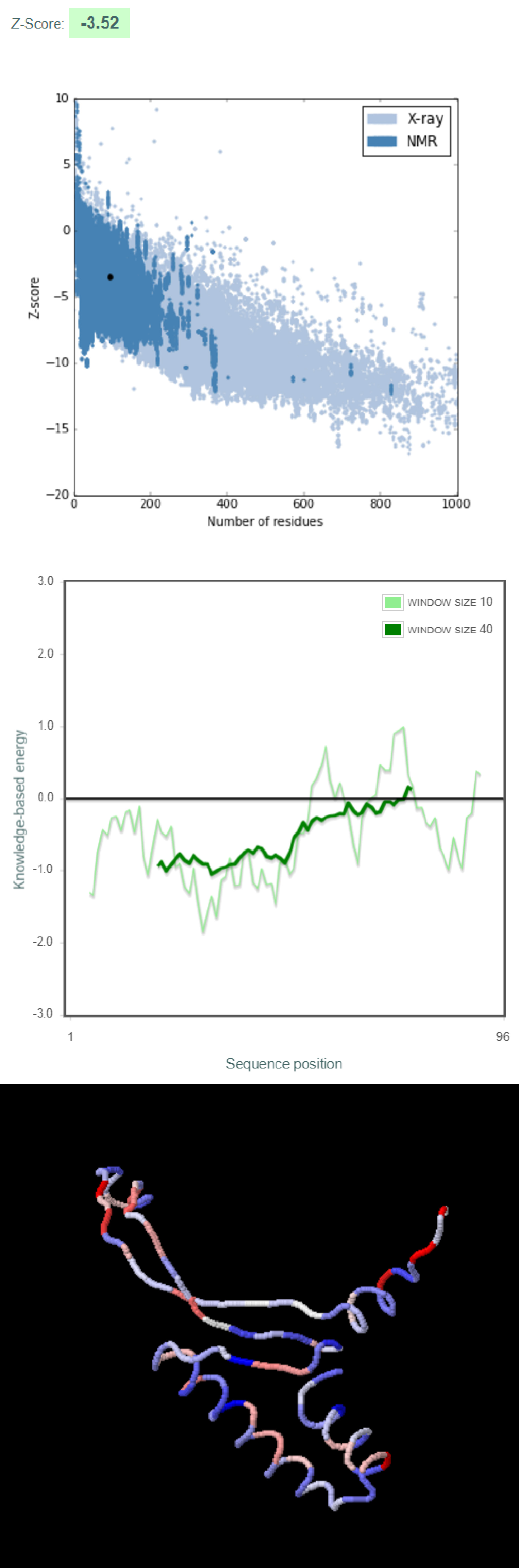

Supplement: S4 File — (ZIP) [file pone.0188037.s004.zip › E4_2 v.jpg]

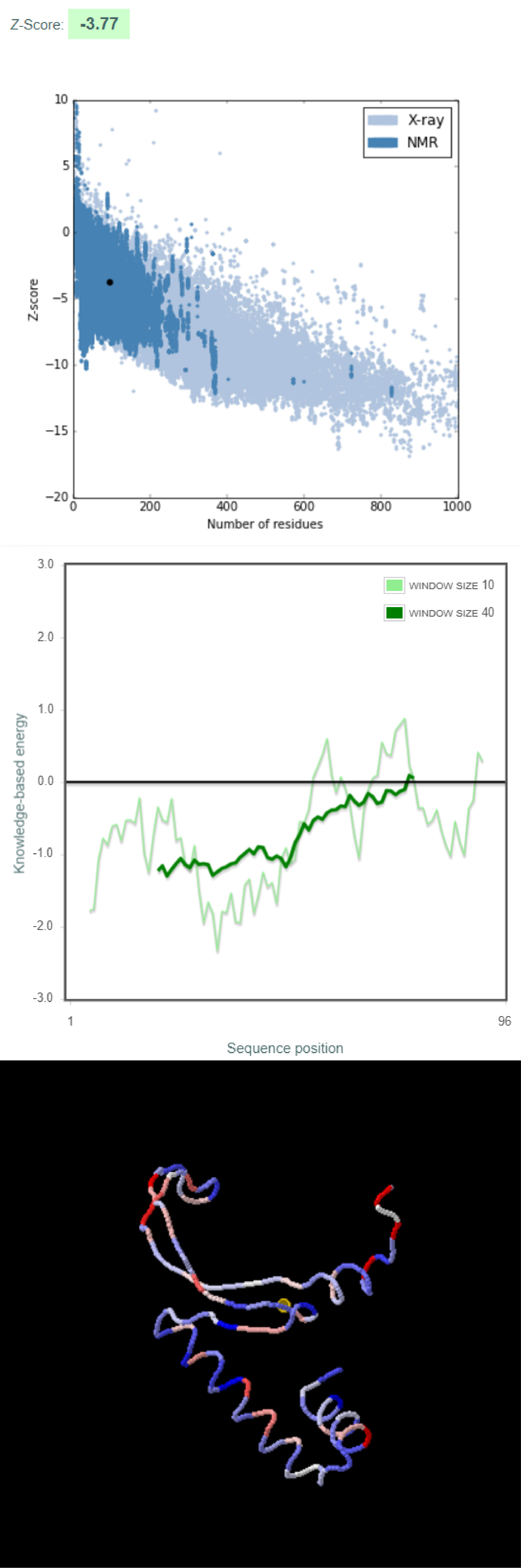

Supplement: S4 File — (ZIP) [file pone.0188037.s004.zip › E4_3 v.jpg]

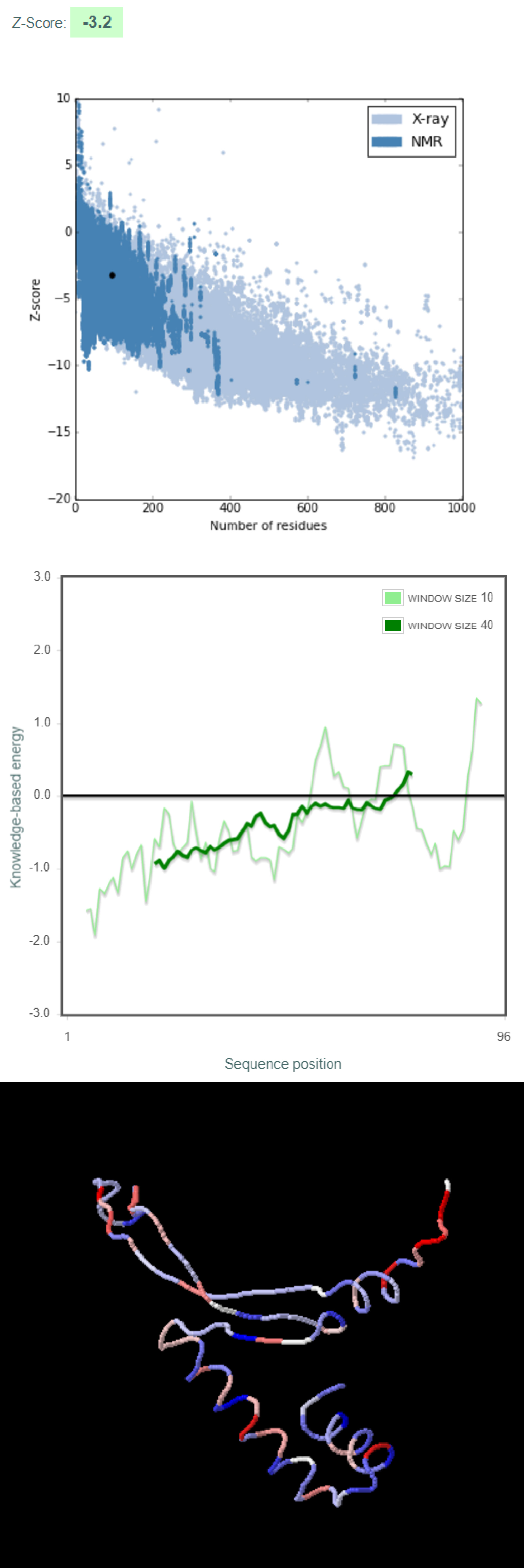

Supplement: S4 File — (ZIP) [file pone.0188037.s004.zip › E4_4 v.jpg]

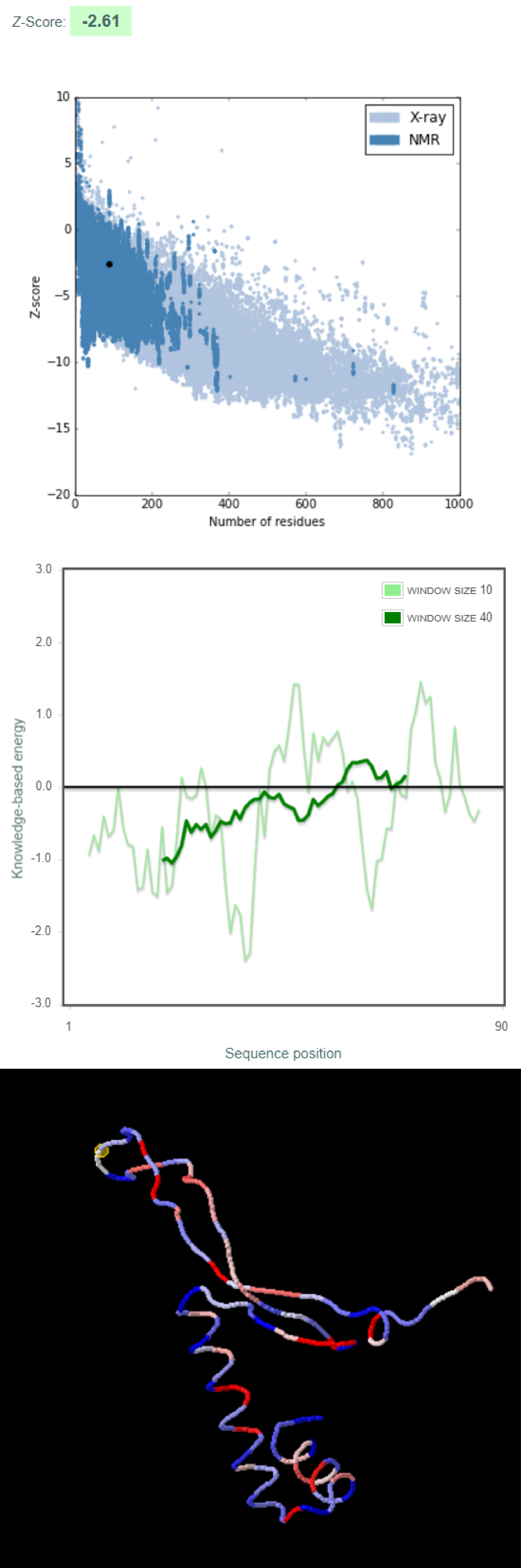

Supplement: S4 File — (ZIP) [file pone.0188037.s004.zip › E_1 v.jpg]

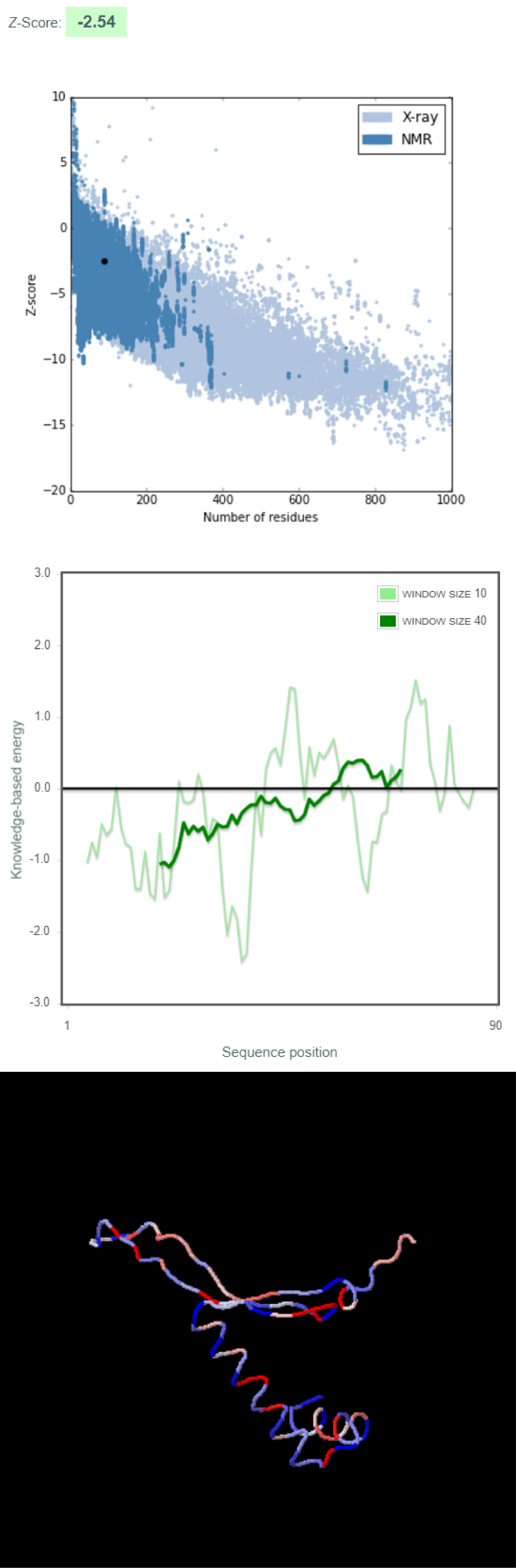

Supplement: S4 File — (ZIP) [file pone.0188037.s004.zip › E_2 v.jpg]

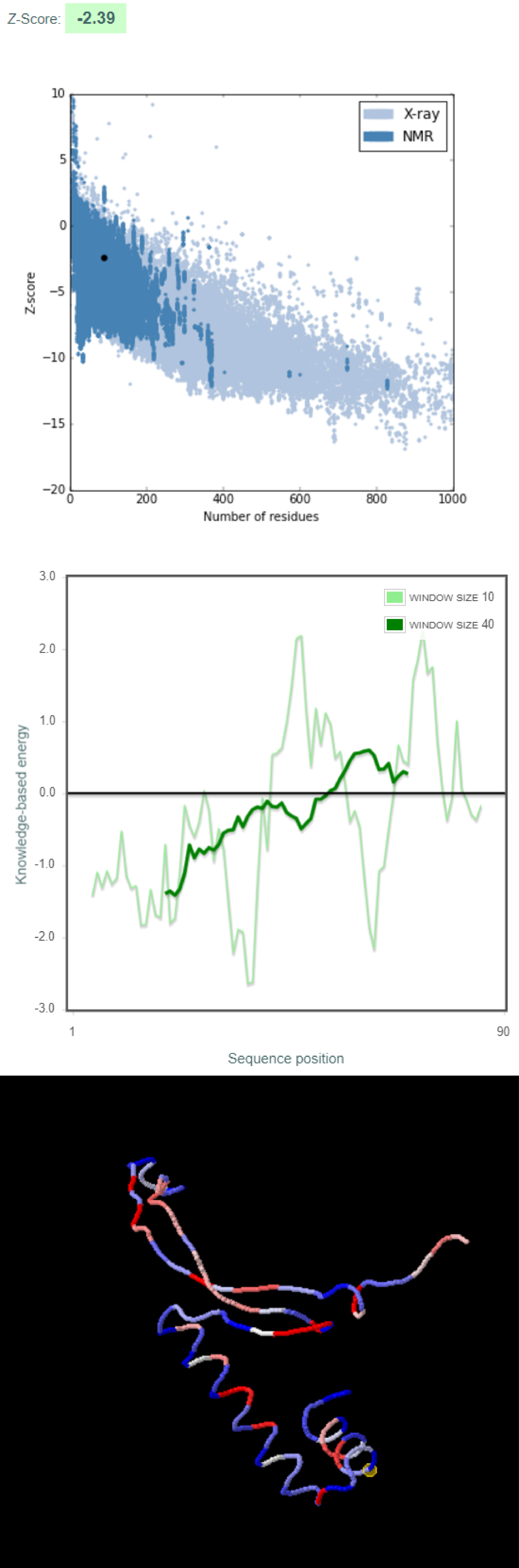

Supplement: S4 File — (ZIP) [file pone.0188037.s004.zip › E_3 v.jpg]
