# Supplementary material for: Comparison of histone-like HU protein DNA-binding properties and HU/IHF protein sequence alignment
Source: PLoS One. 2017 Nov 13;12(11):e0188037. doi: 10.1371/journal.pone.0188037 (PMC5683647; doi:10.1371/journal.pone.0188037)
Supplement: S5 File — (DOCX) [file pone.0188037.s007.docx]

**S5 File. Multiple sequence alignment and clusterization results description of S3 Table**

HU/IHF family protein sequences and annotation were acquired from InterPro ID IPR000119, which represents bacterial histone-like proteins. For each protein sequence results of multiple sequence alignment (MSA) are presented.

S3 Table includes all HU/IHF family protein sequences from InterPro ID IPR000119.

Columns 1-11 present information provided by InterPro: Entry, Entry name, Gene names, Organism, Length, InterPro, and Sequence.

Column 12 presents InterPro annotation of the protein.

Many protein sequences were obtained independently several times. Proteins of the same sequence can belong to the same genera, or the same family, or the same order.

Column 14 shows such case.

Column 13 indicates the row where the protein with the same sequence is placed.

Columns 17-26 show result of multiple sequence alignment performed in this work:

Aligned 90 residue long core sequence (column 18, “aligned sequence”), N- and C-terminal extensions of the protein sequence, their lengths, number of the first residue of the core sequence within the protein sequence (column 23 “start pos”). Protein sequence may contain amino acid residue insertions and deletions compared to the core sequence. Column 22 indicates if protein does not contain them (“zero”), contain 1 to 8 inserted residues (marked as “ins1” – “ins8”), or contain deletions of one (“min1”) , or two (“min2”) residues. Column 24 (“indel pos”) indicates position of residue of the core sequence followed the insertion/deletion. Column 18, “aligned sequence” does not contain insertion/deletion for uniformity. Deleted residue is replaced by symbol “O”, inserted sequences are presented in column 26 (“ins”).

Columns 30 – 35 represents information about taxonomy classification of the species (marked in column 6 “Organism “). Taxonomy attribution is performed in this work based on resource NCBI Taxonomy. https://www.ncbi.nlm.nih.gov/taxonomy.

Columns 45 – 146 provides another presentation of the aligned sequences helpful for further analysis. Symbol “X” stays for the absence of amino acid residues at position indicated in row 28.

Results of clusterization of aligned sequences:

Column 152 represents result of our protein annotation to one of three major groups, IHF_A, IHF_B, and HU.

Coumn 150 compares our result of such protein annotation to HU/IHF groups with InterPro protein annotation.

Column 153 presents results of the further subdivision of protein sequences and indicates attribution of the protein to the clades.

If clade name is “vide” the protein is not classified,

To estimate clusterization significance we compared sequence score for the same clade with the score of another clade which gives the best score, such clade of the second choice is presented in column 153; corresponding major group in column 154; score difference, expressed in score penalty for the incorrect amino acid substitution – in column 155.
